# *LINC02257* regulates malignant phenotypes of colorectal cancer via interacting with miR-1273g-3p and YB1

**DOI:** 10.1038/s41419-024-07259-4

**Published:** 2024-12-18

**Authors:** Mi-So Park, Seong Dong Jeong, Chang Hoon Shin, Soojin Cha, Ahran Yu, Eun Ju Kim, Myriam Gorospe, Yong Beom Cho, Hong-Hee Won, Hyeon Ho Kim

**Affiliations:** 1 Department of Digital Health, Samsung Advanced Institute for Health Sciences and Technology, Sungkyunkwan University, Seoul, 06351 Republic of Korea; 2 Department of Health Sciences and Technology, Samsung Advanced Institute for Health Sciences and Technology, Sungkyunkwan University, Seoul, 06351 Republic of Korea; 3 Laboratory of Genetics and Genomics, National Institute on Aging Intramural Research Program, National Institutes of Health, Baltimore, MD 21224 USA; 4 Department of Surgery, Samsung Medical Center, Sungkyunkwan University School of Medicine, Seoul, 06351 Republic of Korea; 5 Research Institute for Future Medicine, Samsung Medical Center, Seoul, 06351 Republic of Korea; 6 Department of Biopharmaceutical Convergence, Sungkyunkwan University, Gyeonggi-do, 16419 Republic of Korea; 7 Samsung Genome Institute, Samsung Medical Center, Seoul, 06351 Republic of Korea; 8 Department of MetaBioHealth, SKKU Institute for Convergence, Sungkyunkwan University, Gyeonggi-do, 16419 Republic of Korea

**Keywords:** Oncogenes, Long non-coding RNAs

## Abstract

Colorectal cancer (CRC) is the third most common cancer diagnosed and the second leading cause of cancer-related deaths. Emerging evidence has indicated that long non-coding RNAs (lncRNAs) are involved in the progression of various types of cancer. In this study, we aimed to identify potential causal lncRNAs in CRC through comprehensive multilevel bioinformatics analyses, coupled with functional validation. Our bioinformatics analyses identified *LINC02257* as being highly expressed in CRC, and associated with poor survival and advanced tumor stages among patients with CRC. Genome-wide association analysis revealed significant associations between variants near *LINC02257* and CRC, suggesting a causal role for *LINC02257* in CRC. Network analysis identified *LINC02257* as playing a key role in the epithelial-mesenchymal transition pathway. Single-cell RNA sequencing showed that elevated expression of *LINC02257* was associated with a reduced proportion of epithelial cells. In vitro experiments showed that *LINC02257* positively regulated the metastatic and proliferative potential of CRC cells. Mechanistically, *LINC02257* affected CRC malignancy by functioning as a competitive endogenous RNA of microRNAs and RNA-binding proteins. *LINC02257* upregulated SERPINE1 by sequestering tumor suppressive miR-1273g-3p, thereby increasing metastatic and proliferative abilities of CRC cells. Additionally, *LINC02257* directly interacted with YB1 and induced its phosphorylation, thereby facilitating YB1 nuclear translocation. The transcriptional activation of YB1 target genes was associated with the oncogenic functions of *LINC02257*. Taken together, our results demonstrate *LINC02257* as a promising therapeutic target for CRC treatment.

## Introduction

Colorectal cancer (CRC) is the most prevalent gastrointestinal cancer and the second leading cause of cancer-related mortality [1]. Advancements in diagnostics and therapeutics have dramatically increased the survival time of patients with early-stage CRC. However, the 5-year survival rate of patients with advanced CRC remains low [2]. Despite its high mortality rate, an effective treatment for patients with CRC has not yet been developed. Therefore, it is essential to elucidate the molecular mechanisms underlying colon carcinogenesis and explore diagnostic and therapeutic biomarkers [3].

Research on the discovery of therapeutic and diagnostic biomarkers for CRC has shifted from protein-coding genes to non-coding RNAs (ncRNAs). LncRNAs are a class of ncRNAs that are more than 200 nucleotides in length. They are typically transcribed by RNA polymerase II, spliced, 5’ capped, and polyadenylated [4]. Because they are recognized as powerful regulators of gene expression which are responsible for the formation and malignant progression of cancers, including CRC, lncRNAs have attracted the attention of many oncologists interested in the molecular mechanism of carcinogenesis and metastasis. In CRC, a series of lncRNAs have been identified as oncogenes or tumor suppressors, closely related to the malignant phenotypes of CRC by regulating cell proliferation, cell cycle, epithelial-mesenchymal transition (EMT), drug resistance, and metastasis [5].

LncRNAs regulate gene expression in various ways. The role of lncRNAs as a competitive endogenous RNA (ceRNA) has recently garnered significant research interest [6]. The regulatory function of ceRNA is largely dependent on the *cis*-acting elements present within its sequence, enabling it to competitively inhibit microRNAs (miRNAs) or RNA-binding proteins (RBPs) from binding to the target mRNA. Tens of lncRNAs function as ceRNAs and are closely associated with the malignant phenotypes and prognosis of patients with CRC [7–9].

In this study, using The Cancer Genome Atlas (TCGA) and Genotype-Tissue Expression (GTEx) Project data [10], we identified *LINC02257* by combining multiple bioinformatic approaches, including gene expression analysis, survival analysis, expression quantitative trait loci (eQTL) analysis, and co-expression network analysis. Based on the results that LINC02257 is present in both nucleus and cytosol, we identified two mechanisms by which *LINC02257* regulates metastatic and proliferative abilities in CRC. First, *LINC02257* sequesters tumor-suppressive miR-1273g-3p, thereby upregulates SERPINE1. Second, *LINC02257* transactivates Y-box protein 1 (YB1) by inducing its phosphorylation and thereby enabling its nuclear translocation. Taken together, our results demonstrate *LINC02257* as a promising target for the treatment of CRC.

## Materials and methods

Detailed analytical and experimental methods are described in Supplementary Methods.

### Transcriptome data collection

TCGA catalogs the genomic data of multiple cancers operated by the National Cancer Institute (NCI) and the National Human Genome Research Institute (NHGRI). We used the transcriptome and clinical data of CRC and normal-tissue samples, and excluded metastatic or recurrent tumor samples.

The initial number of transcriptome data points was 478 and 41 for colon tumors and normal tissues, respectively, while the total number of clinical data points was 461 owing to redundant tumor data from the same patients. To eliminate redundant data, we specifically selected samples with the highest lexicographical number of TCGA barcodes, which served as the biospecimen identifier, except for formalin-fixed paraffin-embedded samples. The filtering criteria were provided by the GDAC Broad Institute (https://gdac.broadinstitute.org/runs/gdc/report_2017_08_02/TCGA-COAD_Replicate_Samples.html). If there are multiple aliquots associated with the chosen RNA analyte, we selected the aliquots that were preserved by freezing and had the later plate number. After excluding samples lacking clinical information on follow up days or tumor stage, 482 samples (442 tumors and 40 control samples) remained (Supplementary Fig. S1).

### Normalization and differentially expressed gene (DEG) analysis

For our gene expression analysis, we used read-count data from samples of both CRC and normal tissue (total number of genes = 60,483). Genes with read counts <10 were excluded, and the gene expression level values of the remaining genes were normalized across the samples and grouped into tumor and normal tissues. We used the maximum a posteriori (MAP) function to estimate gene-specific dispersion across samples and the log_2_ fold change (LFC) of each gene between the two groups using the DESeq2 package (v 1.22.2). After fitting the LFC as a coefficient in the negative binomial generalized linear model, we tested the significance of these coefficients in each gene using the Wald test, as the default setting for DESeq2. For the identification of differentially expressed (DE) lncRNA, cases that met the criteria of |LFC | ≥ 1 and adjusted *P* < 0.05 were selected. In addition, an independent CRC data set, GSE146009 from Gene Expression Omnibus (GEO) that provides only the numeric data, was imported into ‘edgeR’ for DE analysis [11]. Differential expression was assessed using an exact negative binomial test and further adjusted using the Benjamini-Hochberg (BH) procedure.

### Prognostic analysis

To investigate the prognostic effects of the identified lncRNAs on each patients with cancer, we performed Cox proportional hazard regression analysis using the ‘Survival’ R package (v 3.1-8). For each lncRNA, samples were classified into groups with high- or low-expression based on the median expression value and the hazards between the groups were compared. Spearman’s correlation and Cochran-Armitage trend tests were used to assess whether the expression levels of candidate lncRNAs increased as the tumor stage advanced. For the Cochran-Armitage trend test, the data were divided into two groups according to the median expression value [12, 13]. To test the association between lncRNA expression and microsatellite instability (MSI), Spearman’s correlation was used after removal of samples whose MSI information was “not reported” or “indeterminate”. A significant association of lncRNAs with prognosis was examined using a BH-adjusted *P*-value of <0.05.

### Cell culture and transfection

CRC cell lines (DLD1, HCT116, RKO, and HT29) were cultured in Dulbecco’s modified Eagle’s medium (Gibco, Waltham, MA, USA). For transfection, Lipofectamine 2000 (Invitrogen, Carlsbad, CA, USA) was used. The siRNA sequences used in this study are shown in Supplementary Table S1.

### Western blot and RT-qPCR analyses

Protein and mRNA expression levels were determined using western blotting and RT-qPCR, respectively. Information regarding the antibodies and primer sequences used is provided in Supplementary Tables S2 and S3, respectively.

### Determination of malignant phenotypes

Metastatic potential including invasive and migratory abilities was determined using the BD Biocoat™ Matrigel invasion chamber (BD Bioscience, San Jose, CA, USA) and Transwell® permeable supports (Corning, Rockville, MD, USA), respectively [14]. Cell proliferation was assessed using a colony formation assay and cell counting.

### Cellular fractionation

The localization of *LINC02257* was examined using a cellular fractionation assay [15]. The levels of α-tubulin and lamin B were assessed to determine the composition of cytosolic and nuclear lysates, respectively.

### Ribonucleoprotein immunoprecipitation (RIP)

Argonaute 2 (AGO2) RIP assay was conducted to determine the binding of miR-1273g-3p to *LINC02257* and *SERPINE1* mRNA. The interaction between YB1 and *LINC02257* was verified using RIP assay [15].

### Antisense oligonucleotide (ASO) pulldown assay

ASOs that recognize *LINC02257* were designed and used to identify *LINC02257*-associated miRNAs and RBPs. A schematic of ASO pulldown experiment is shown in Supplementary Fig. S22.

### Luciferase reporter assay

The sequence-specific interaction of miR-1273g-3p was verified using luciferase vectors (pmirGLO dual-luciferase vectors; Promega, Madison, WI, USA) containing wild-type or mutated sequences of the miR-1273g-3p miRNA recognition element (MRE) (detail information in Supplementary Fig. S6).

## Results

### Identification of candidate lncRNAs for CRC

**Fig. 1:**
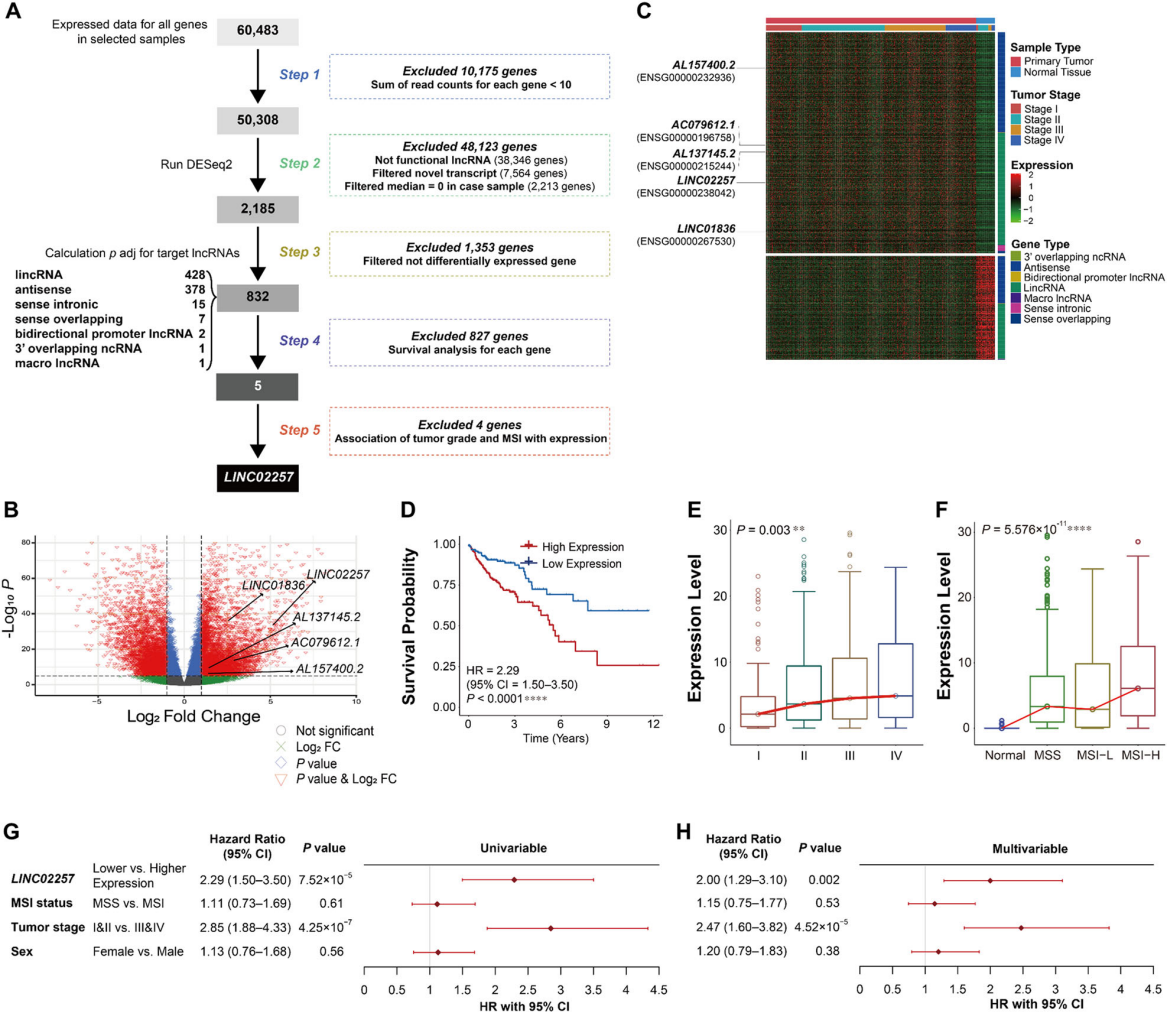
Overview of the process of identifying candidate causal long non-coding RNAs for colon cancer. **A** The systematic analyses process to identify differentially expressed (DE) lncRNAs between colon cancer and normal tissue samples is illustrated. **B** Filtered genes are represented using volcano plot. Genes exhibiting |log2 fold change | ≥ 1, and *p*-value < 10^−6^ are highlighted in red, those with *p*-value < 10^−6^ (blue), and |log2 fold change | ≥ 1 in green, and non-significant genes in grey. Significantly prognosis-associated 5 lncRNAs were annotated. **C** Clustered samples are represented using heatmap, revealing 832 DE lncRNAs in colon tumors compared with normal tissues. Sample types and tumor stages are indicated in the top bar, while gene types are denoted on the right. Five annotated lncRNAs (*AL157400.2*, *AC079612.1*, *AC137145.2*, *LINC02257*, and *LINC01836*) exhibit higher expression and are associated with poor prognosis in colon cancer (false discovery rate < 0.05). **D** Kaplan-Meier curves of overall survival in patients with colon cancer were generated based on high and low expression of *LINC02257*. For individual lncRNAs, patient samples of colon cancer were divided into groups with either high or low expression levels of each lncRNAs based on the median expression value. **E**, **F** Expression levels of *LINC02257* was increased according to (**E**) tumor stages and (**F**) MSI categories. (****P < 0.0001, ***P < 0.0005, **P < 0.005, *P < 0.05). Dots represent outlier samples whose expression levels are beyond the minimum and maximum values. The minimum value is the 25th percentile of the data minus 1.5 times the interquartile range (IQR) and the maximum value is the of 75th percentile of the data plus 1.5 times the IQR. **G**, **H** Forest plots describe the hazard ratio (HR) and 95% confidence interval (CI) of clinical and genetic factors. Univariate (**G**) and multivariate (**H**, removed 6 sample that have “not reported” in MSI status). Cox’s proportional hazard regression was used to assess association with overall survival.

To identify candidate causal lncRNAs, we systematically identified lncRNAs that were differentially expressed (DE) between normal and cancerous tissues and exhibited association with survival and tumor stages in CRC using RNA-seq data from TCGA cohort (Fig. 1A and Supplementary Fig. S1A). We initially identified 832 DE lncRNA ( | LFC | ≥ 1 and adjusted *P* < 0.05); 566 were over-expressed while 266 were downregulated in tumor tissues (Fig. 1B, C and Supplementary Table S4). Next, the association of these 832 lncRNAs with the survival of patients with CRC was investigated; the expression levels of five lncRNAs were associated with overall survival (OS) (Fig. 1D and Supplementary Fig. S1B). Increased expression levels of four lncRNAs (*AL137145.2*, *AL157400.2*, *LINC02257*, and *LINC01836*) were associated with a worse prognosis, whereas higher expression of *AC079612.1* was associated with a good OS. Third, we explored the association between lncRNA expression and CRC stages. High expression levels of *LINC02257* and *LINC01836* were positively correlated with advanced tumor stages (adjusted *P* = 0.0022 and adjusted *P* = 0.0023, respectively, Cochran-Armitage trend test) (Fig. 1E and Supplementary Fig. S1C). Finally, significant positive associations between expression of two lncRNAs (*AL137145.2* and *LINC02257*) and the MSI class were observed in patients with MSI data (adjusted *P* = 1.39 × 10^−10^ and adjusted *P* = 1.39 × 10^−10^, Spearman’s correlation) (Fig. 1F and Supplementary Fig. S1D). In Cox’s proportional hazard regression analysis adjusted for sex, tumor stage, and MSI, high *LINC02257* expression was associated with OS (hazard ratio [HR] = 2.00, 95% confidence interval [CI] = 1.29–3.10, *P* = 0.002) (Fig. 1G, H).

**Fig. 2:**
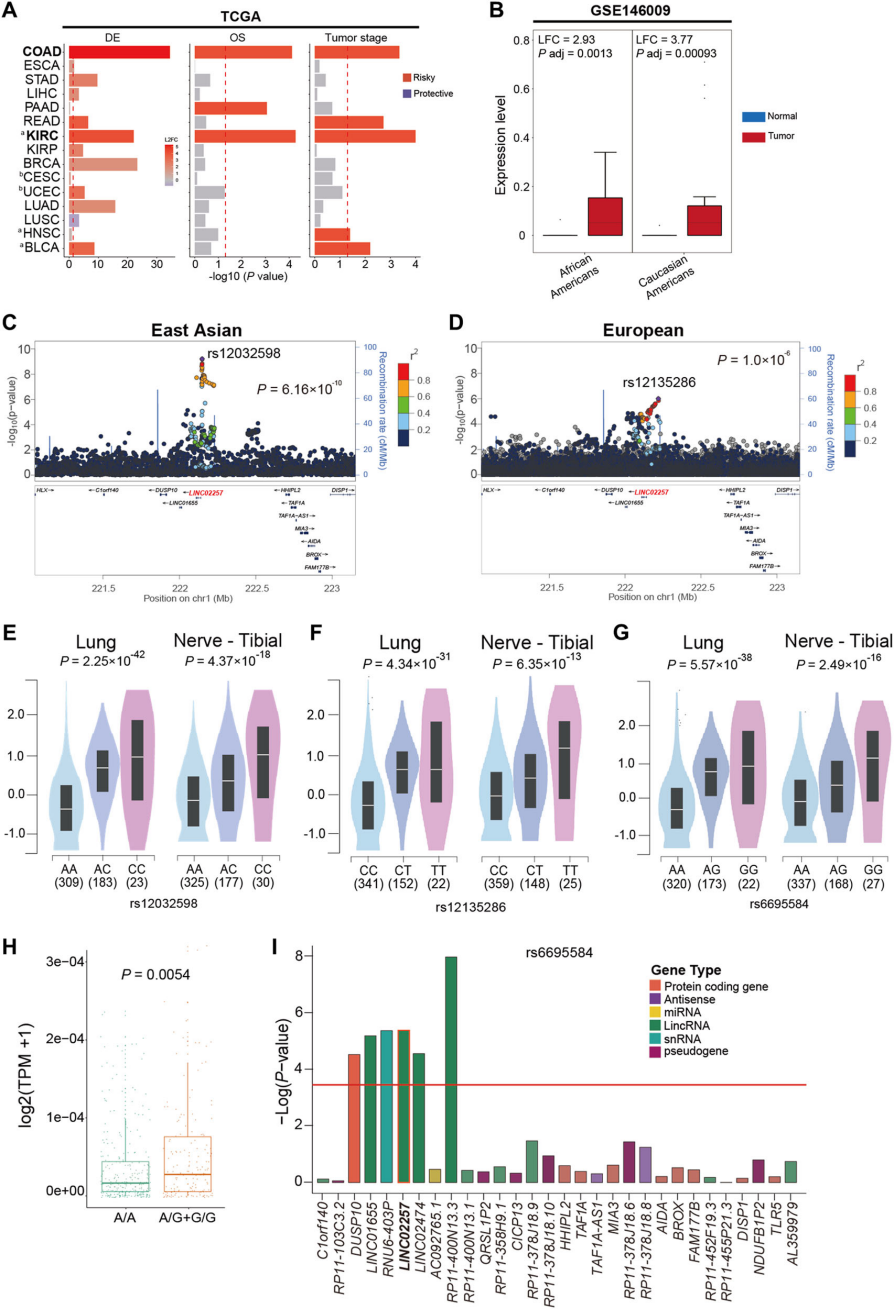
Multi-validated analysis of long non-coding RNAs associated with colon cancer. **A** Bar plot of *p*-values calculated from differentially expressed (DE), overall survival (OS), and tumor stage association analysis for *LINC02257* in pan-cancer data of the Cancer Genome Atlas (TCGA). The red dashed line indicates a *p*-value of 0.05. The log-rank test was used to calculate OS in survival analysis. Cochran-Armitage trend tests evaluated expression effects in relation to tumor stages. **B** *LINC02257* expression in colon cancer among African Americans (AA) and Caucasian Americans (CA) from the GEO146009 dataset. **C**, **D** Colorectal cancer risk variants (diamonds) were identified based on Genome-wide association studies (GWAS). rs12032598, within 1 Mb of *LINC02257*, was found to be significantly associated with colorectal cancer in the East Asian GWAS (**C**). rs12135286, also within 1 Mb of *LINC02257*, exhibited significant association with colorectal cancer in the European GWAS (**D**). **E**–**G** Violin plot of gene expression levels of *LINC02257* according to single nucleotide polymorphisms (SNPs). Increased *LINC02257* expression levels were consistently associated with the risk alleles of SNPs. rs12032598 associated with lung (*p* = 2.3 × 10^−42^) and nerve (*p* = 4.4 × 10^−18^) tissues (**E**), rs12135286 associated with lung (*P* = 4.3 × 10^−31^) and nerve (*P* = 6.4 × 10^−13^) tissues (**F**), and rs6695584 associated with lung (*P* = 5.6 × 10^−38^) and nerve (*P* = 2.5 × 10^−16^) tissues (**G**) from the GTEx database. **H** The analysis of *LINC02257* expression according to the genotypes of rs6695584 in TCGA. **I** *p*-value of eQTL associated with rs6695584 and genes within 1 Mb of *LINC02257*. Abbreviations: COAD colon adenocarcinoma, ESCA esophageal carcinoma, STAD stomach adenocarcinoma, LIHC Liver hepatocellular carcinoma, PAAD Pancreatic adenocarcinoma, READ Rectum adenocarcinoma, KIRC kidney renal clear cell carcinoma, KIRP kidney renal papillary cell carcinoma, BRCA breast invasive carcinoma, CESC cervical squamous cell carcinoma and endocervical adenocarcinoma, UCEC uterine corpus endometrial carcinoma, LUAD lung adenocarcinoma, LUSC lung squamous cell carcinoma, HNSC head and neck squamous cell carcinoma, BLCA bladder urothelial carcinoma. ^a^The American Joint Committee on Cancer (AJCC) stage was used (Kidney renal clear cell carcinoma, head and neck squamous cell carcinoma, and bladder urothelial carcinoma). ^b^For gynecological cancer, the International Federation of Gynecology and Obstetrics (FIGO) stage was used (Cervical squamous cell carcinoma and endocervical adenocarcinoma, and uterine corpus endometrial carcinoma).

Next, we examined the associations between dysregulated *LINC02257* expression and various types of cancer using two independent datasets: TCGA RNA-seq data for various cancers, and GEO data for colon cancer (Supplementary Table S5). *LINC02257* was upregulated in tumor tissues across most organs (Fig. 2A). Notably, upregulation of *LINC02257* was significantly associated with advanced tumor stages in rectum, kidney, head and neck, and bladder cancers, and correlated with poor OS in patients with pancreas and kidney cancers (*P* < 0.005, Spearman’s correlation; Cochran-Armitage trend test; survival analysis). In the association analyses of DE, OS, and tumor stages, *AC079612*, *AL137145*, and *AL157400* did not consistently exhibit associations with pan-cancer (Supplementary Fig. S2). Although *LINC01836* is upregulated in colon and rectal cancers, it was down-regulated in kidney, lung, and head and neck cancers. Additionally, high expression of *LINC01836* was associated with good OS but not with advanced tumor stages in other organs (Supplementary Fig. S2). Moreover, in an independent GSE146009 colon cancer RNA-seq data [16], compared with normal tissues, *LINC02257* exhibited significantly higher expression levels in colon cancer tissue sample in both African American and Caucasian American populations (Fig. 2B). These results suggest that *LINC02257* may be a key candidate lncRNA in pan-cancer carcinogenesis.

### Genetic variants associated with colon cancer and expression of *LINC02257*

Subsequently, we investigated whether genetic variants in the *LINC02257* region were associated with colon cancer using summary statistics data from genome-wide association studies (GWAS) of colorectal cancer in East Asian and European populations. In the East Asian GWAS, a lead single-nucleotide polymorphism (SNP), rs12032598, exhibited a significant association with the risk of CRC (*P* = 6.16 × 10^−10^, odds ratio [OR] = 1.155, 95% CI = 1.104–1.208) (Fig. 2C and Supplementary Fig. S3A) [17]. The European GWAS also demonstrated a significant association between rs12135286 and colon cancer in close proximity to *LINC02257* (*P* = 9.50 × 10^−7^, OR = 1.141, 95% CI = 1.082–1.203) (Fig. 2D and Supplementary Fig. S3B) [18]. All variants in high linkage disequilibrium with rs12032598 and rs12135286 were located near the start site of *LINC02257*, suggesting a potential association of these variants with *LINC02257* expression as a *cis*-expression quantitative trait (eQTL) (Fig. 2C, D). Additionally, we examined whether the expression of other genes within 1 mega-base pair (Mb) of *LINC02257* was associated with colon cancer. Among 25 genes located near *LINC02257* (Supplementary Table S6), eight genes, *C1orf140*, *DUSP10*, *LINC01655*, *RNU6-403P*, *LINC02474*, *LINC01705*, *TAF1A*, and *AL592148.1*, showed significantly higher DE levels in CRC than in normal tissues. Of these, only *LINC02474* demonstrated significant associations with poor survival (adjusted *P* = 0.018) and advanced tumor stage (adjusted *P* = 0.023, Cochran-Armitage trend test) (Supplementary Table S6). At this locus, only *LINC02257* was significant in all analyses, including the GWAS and DE analysis, and was associated with a poor prognosis.

To identify eQTL for the two GWAS variants (rs12032598 and rs12135286) located near *LINC02257*, we used genotype and transcriptome datasets from the GTEx database (v8). Expression levels of the three genes were associated with genotypes of variants in three tissues: rs12032598 was associated with *LINC02257* in lung (*P* = 2.2 × 10^−42^) and nerve (*P* = 4.4 × 10^−18^) tissues (Fig. 2E), and with LINC01705 in cell-cultured fibroblasts (*P* = 5.7 × 10^−7^). rs12135286 was associated with *LINC02257* in lung (*P* = 4.3 × 10^−31^) and nerve (*P* = 6.4 × 10^−13^) tissues (Fig. 2F), and with *LINC01705* in cell-cultured fibroblasts (*P* = 3.2 × 10^−7^) and *DUSP10* in nerve tissues (*P* = 1.2 × 10^−5^). The eQTL showed that the expression of *LINC02257* increased among carriers of the rs12032598 C (risk allele) and rs12135286 T (risk alleles), except in the esophageal mucosa (Supplementary Fig. S4–5).

We further examined the association of GWAS lead SNPs with *LINC02257* expression using the TCGA data. As the GWAS variants (rs12032598 and rs12135286) were not available in the TCGA genotype data, we assessed rs6695584, rs6691195, and rs17011200, which exhibit strong linkage disequilibrium (LD) with lead SNPs (r2 > 0.8, from the 1000 Genomes Project) (Fig. 2G and Supplementary Fig. S6). The expression of *LINC02257* was higher among carriers of rs6698857 GG or GA genotypes compared to AA carriers (*T*-test, *P* = 0.0054, Fig. 2H) and *cis*-eQTL analysis revealed an association (*P* = 8.03 × 10^−4^) between rs6695584 and the covariate-corrected expression of *LINC02257* (Fig. 2I).

### Functional annotation and cell-type inference from the co-expression network in colon cancer

**Fig. 3:**
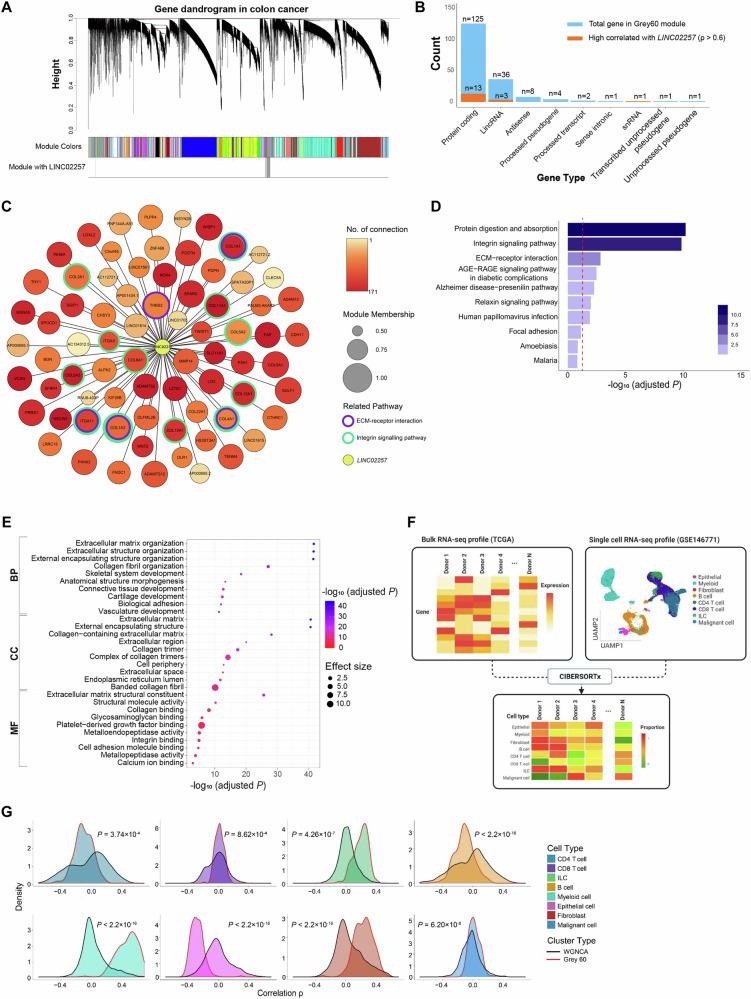
Gene clustering via WGCNA and functional analysis. **A** Clustering results were obtained from the colon cancer dataset using weight gene co-expression network analysis (WGCNA). Co-expressed genes are grouped into 69 modules, each denoted by a distinct color. The red line signifies the cutoff value used for gene clustering in the dendrogram. **B** Counts of genes per gene types in the ‘Grey60’ module, which includes *LINC02257*. Blue bars represent total genes, and orange bar indicate genes with p (rho) > 0.6 (Spearman’s correlation). **C** Network plot of the ‘Grey60’ module utilizing Cytoscape. Circles represent the nodes connected to *LINC02257*, and yellow circle represents *LINC02257*. **D** Pathway and Gene Ontology (GO) analysis for genes co-expressed with *LINC02257* using WebGestalt. Pathway enrichment analysis based on the Kyoto Encyclopedia of Genes and Genomes and Panther pathways was performed for genes within the ‘Grey60’ module. **E** GO analysis results for biological processes, cellular components, and molecular functions. **F** Workflow of CIBERSORTx to examine the cell proportion of each donor in the Cancer Genome Atlas. **G** Comparison between the distribution of Spearman’s correlation coefficients of two gene sets. Red board line; ‘Grey60’ module, and black board line; genes into WGCNA.

LncRNAs have emerged as key players in cancer pathophysiology, engaging in intricate interactions with various genetic elements such as DNA, mRNAs, miRNAs, and proteins [19]. To detect genes that interacted with *LINC02257* and understand their associated mechanisms, we performed network analysis based on the gene expression data (Fig. 3A and Supplementary Fig. S7) [20]. Of the total 69 identified co-expression modules, representing clusters of highly interconnected genes, we focused on the ‘Grey60’ that includes *LINC02257* which was shown to be a promising candidate causal gene. The ‘Grey60’ module contained 179 genes, and the module membership of *LINC02257* was 0.52 in the ‘Grey60’ module (Fig. 3B, C).

To elucidate the functions of genes interacting with *LINC02257*, we performed pathway enrichment analysis of the 179 genes in the ‘Grey60’ module [21]. The top five significantly enriched pathways included protein digestion and absorption (*P* = 1.45 × 10^−13^), integrin signaling pathway (*P* = 6.62 × 10^−13^), extracellular matrix (ECM) receptor interaction (*P* = 1.01 × 10^−5^), AGE-RAGE signaling pathway during diabetic complications (*P* = 2.99 × 10^−5^), and Alzheimer’s disease-presenilin pathway (*P* = 6.00 × 10^−5^) (Fig. 3D). Among the significantly enriched pathways, integrin signaling of transmembrane receptors promoting cell-ECM adhesion, and ECM receptor interactions were functionally related to the prognosis and progression of colon cancer [22, 23]. We also examined the gene ontology (GO) in three categories: biological processes (BP), cellular components (CC), and molecular functions (MF) [24]. The most significant GO term in each category was ECM (GO:0031012, *P* = 1.34 × 10^−41^) in CC, ECM organization (GO:0030198, *P* = 1.43 × 10^−42^) in BP, and ECM structural constituents in MF (GO:0005201, *P* = 1.89 × 10^−26^), respectively (Fig. 3E). Thirteen mRNAs within the ‘Grey60’ module exhibited strong correlation with *LINC02257* (rho > 0.6, Spearman’s Correlation) and were associated with ECM signaling. However, the GWAS did not reveal significant SNPs in the genomic regions of the 13 mRNAs (Supplementary Fig. S8–9).

As co-expression modules in bulk tissues may be determined by cell composition, we further investigated the estimated cell proportions in colorectal tissue to identify the cellular mechanism underlying the role of *LINC02257* (Fig. 3F) [25]. Our results indicated positive correlations between *LINC02257* expression and the proportions of innate lymphoid cells (ILCs), myeloid cells, and malignant cells, whereas a negative correlation was observed with epithelial cells (Spearman’s correlation, *P* < 0.05) (Supplementary Fig. S10). Considering that during EMT, epithelial cells lose stemness properties and reorganize their cytoskeleton for dynamic elongation and motility [26, 27], our findings indicates that decreased epithelial cells might be associated with cancer metastasis, and *LINC02257* might play an essential role in EMT in cancer progression. Additionally, a two-sided *t*-test was conducted to determine differences in the correlation coefficient distributions across each cell type between the 12,141 genes included in the Weighted Gene Co-expression Network Analysis (WGNCA) and the 179 genes in the ‘Grey 60’ module. All cell types exhibited significantly different distributions (Fig. 3G). While the average correlation of the total genes with each cell proportion was close to zero, the genes in the ‘Grey60’ module showed significant correlations with cell proportions (Supplementary Fig. S10).

### *LINC02257* positively regulates the malignant phenotypes of CRC cells

**Fig. 4:**
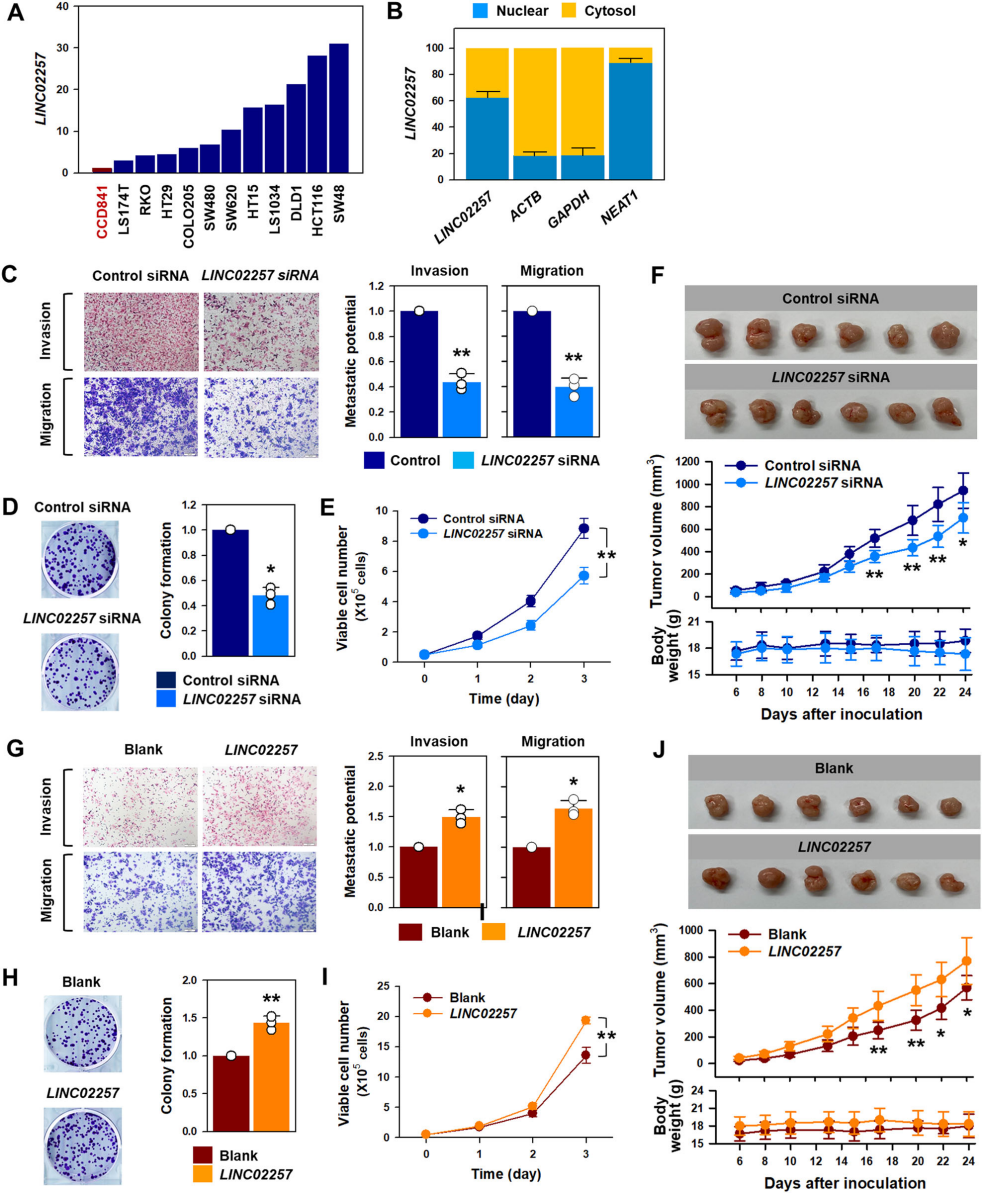
*LINC02257* is closely associated with malignant phenotypes in DLD1 cells. **A** The expression level of *LINC02257* in various colorectal cancer cells was examined using RT-qPCR. **B** Cellular fractionation assay was performed to determine the localization of *LINC02257* in DLD1 cells. **C**, **G** *LINC02257* knockdown (**C**) or overexpression (**G**) experiments followed by a Transwell assay was performed to examine its role in metastatic potential. Invasive and migratory abilities were determined by quantifying the number cells that invaded or migrated. **D**, **E**, **G**, **H** After transfection of DLD1 cells as performed above, cell proliferation was examined by colony forming assay (**D** for knockdown; **H** for overexpression) and cell counting (**E** for knockdown; **I** for overexpression). **F**, **J** To investigate the oncogenic role of LINC02257 in mouse model, the subcutaneous injection experiments were conducted. Tumor size was measured using a caliper, and tumor volume was calculated using the following formula: (short length × long length × width)/2. Data are expressed as mean ± standard deviation and the statistical significance was represented as follows: *p < 0.05, **p < 0.01.

The results of the bioinformatic analysis compelled us to investigate the function of *LINC02257* in the malignant phenotypes of CRC. Initially, the expression levels of *LINC02257* were assessed in normal colon epithelial cells (CCD841) and various CRC cells, revealing that *LINC02257* was highly expressed in CRC cells compared to normal colon epithelial cells (CCD841) according to RT-qPCR results (Fig. 4A and Supplementary Fig. S11A). The proliferation rate and invasive ability of CRC cells were measured to confirm the relationship between the expression of *LINC02257* and the degree of malignancy of colon cancer cells. HCT116 and DLD1 cells exhibiting high expression levels of *LINC02257* showed a faster growth rate and higher invasive ability than HT29 and RKO cells, where *LINC02257* was expressed at relatively low levels (Supplementary Fig. S11B). These results demonstrate that *LINC02257* is closely associated with the malignant phenotypes of CRC cells. Prior to exploring the functional role of *LINC02257* in CRC, we examined its cellular localization, which revealed that *LINC02257* was slightly more localized to the nucleus (Fig. 4B). Therefore, we focused on the cytosolic and nuclear functions of *LINC02257*.

To determine the effects of *LINC02257* knockdown on the metastatic and proliferative abilities of CRC cells, three independent siRNAs were designed (Supplementary Fig. S12A). All the designed siRNAs efficiently decreased the expression level of *LINC02257* in DLD1 cells (Supplementary Fig. S12B). Representative results using *LINC02257* siRNA #1 are shown in the main figure and the all siRNA results are provided in the Supplementary Figures. Knockdown of *LINC02257* weakened the metastatic potential, including the invasive and migratory abilities of the DLD1 cells (Fig. 4C). All three siRNAs induced a similar inhibitory effects on the invasive and migratory abilities of DLD1 cells (Supplementary Fig. S13A). In addition to the Transwell migration assay, the wound closure assay indicated that DLD1 cells with low expression of *LINC02257* exhibited decreased migratory activity. Knockdown of *LINC02257* also decreased the number of colonies (Fig. 4D and Supplementary Fig. S13B) and reduced the proliferation rate (Fig. 4E and Supplementary Fig. S13C). These results were verified by examining the effect of *LINC02257* knockdown in HCT116 cells. Consistent with the results observed in DLD1 cells, *LINC02257* knockdown inhibited the metastatic and proliferative abilities of the HCT116 cells (Supplementary Fig. S14). To investigate the long-term effect of *LINC02257*, we further conducted the subcutaneous injection experiment. It showed that the tumor volume of the group injected with *LINC02257*-silenced DLD1 cells was significantly reduced compared to negative control group (Fig. 4F). There was no difference of body weight between two groups.

Conversely, overexpression of *LINC02257* potentiated the invasive and migratory abilities of DLD1 cells in a dose-dependent manner (Fig. 4G and Supplementary Fig. S15). An enhanced metastatic potential was also observed in *LINC02257*-overexpressing HCT116 cells (Supplementary Fig. S16A). Overexpression of LINC02257 increased the colony-forming ability and accelerated the proliferation rate in DLD1 (Fig. 4H, I) and HCT116 cells (Supplementary Fig. S16B, C). Additionally, we also found that the invasive and migratory abilities of RKO cells expressing low levels of *LINC02257* were augmented by its overexpression (Supplementary Fig. S17). The effect of *LINC02257* overexpression on tumor formation was examined using the subcutaneous injection mouse model. The tumor volume of the group injected with *LINC02257*-overexpressing DLD1 cells was significantly increased compared to blank control without a change of body weight (Fig. 4J). These results demonstrate that *LINC02257* is responsible for malignant phenotypes such as high metastatic potential and rapid proliferation of CRC cells.

### *SERPINE1* is involved in the regulation of malignancy in CRC by *LINC02257*

**Fig. 5:**
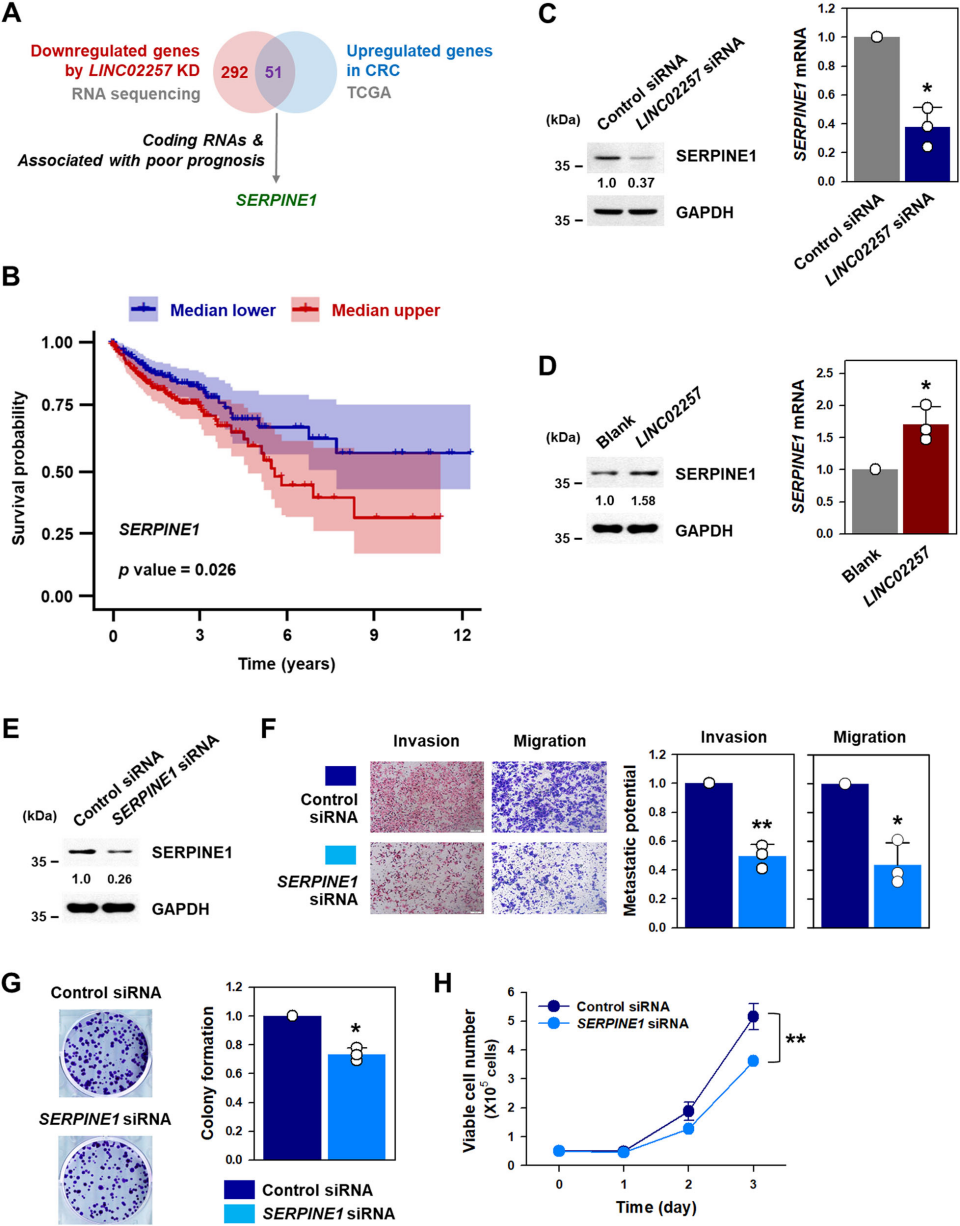
SERPINE1, a target of *LINC02257*, is involved in malignant phenotypes of DLD1 cells. **A** *SERPINE1* was selected as a target of *LINC02257* by comparing the list of genes downregulated by *LINC02257* knockdown and gene that are typically upregulated in colorectal cancer (CRC). **B** Kaplan-Meier overall survival (OS) plot of *SERPINE1* expression in patients with CRC from the Cancer Genome Atlas (TCGA). **C**, **D** Knockdown of *LINC02257* was performed by transfecting DLD1 cells with control or *LINC02257*-targeting siRNA (**C**). For overexpression of *LINC02257*, DLD1 cells were transfected with blank or overexpressing vector (**D**). Expression levels of SERPINE1 protein and mRNA were determined using western blot and RT-qPCR analyses, respectively. **E**–**H** DLD1 cells were transfected with control or *SERPINE1*-targeting siRNA. Expression levels of SERPINE1 protein were determined by western blot (**E**). Metastatic potential including invasive and migratory abilities was determined by the Transwell assay (**F**). Proliferative ability was assessed by a colony forming assay (**G**) and by counting the number of viable cells (**H**). Data are expressed as mean ± standard deviation and the statistical significance was represented as follows: *p < 0.05, **p < 0.01.

RNA-seq analysis revealed 343 genes were downregulated in *LINC02257*-silenced DLD1 cells, and 51 genes of them were generally upregulated in CRC (Fig. 5A and Supplementary Fig. S18A). *SERPINE1*, which encodes plasminogen activator inhibitor-1 (PAI-1), was identified as the target gene of *LINC02257* by comparing the expression levels of 47 coding genes (except for four non-coding genes) and analyzing their association with the prognosis of patients with CRC. Survival analysis indicated that the expression of *SERPINE1* was closely associated with the poor prognosis of patients with CRC (Fig. 5B and Supplementary Fig. S18B). To verify whether *SERPINE1* is a downstream target of *LINC02257*, protein and mRNA levels of SERPINE1 were determined using western blotting and RT-qPCR in *LINC02257*-silenced and overexpressed DLD1 cells. Knockdown of *LINC02257* decreased SERPINE1 protein and mRNA levels (Fig. 5C and Supplementary Fig. S18C). Conversely, *LINC02257*-overexpressed DLD1 cells exhibited increased SERPINE1 protein and mRNA levels (Fig. 5D). To examine whether SERPINE1 is involved in the control of the malignant phenotypes of CRC, the metastatic and proliferative abilities of SERPINE1-silenced DLD1 cells were assessed. The transfection of DLD1 cells with *SERPINE1*-targeting siRNA significantly decreased SERPINE1 expression (Fig. 5E). SERPINE1 knockdown inhibited the metastatic potential, including invasive and migratory abilities, of DLD1 cells (Fig. 5F), decreased the number of colonies (Fig. 5G), and slowed cell proliferation (Fig. 5H). These results indicate that *LINC02257* regulates metastatic and proliferative abilities by regulating SERPINE1.

### *LINC02257* upregulated SERPINE1 by sequestering miR-1273g-3p

**Fig. 6:**
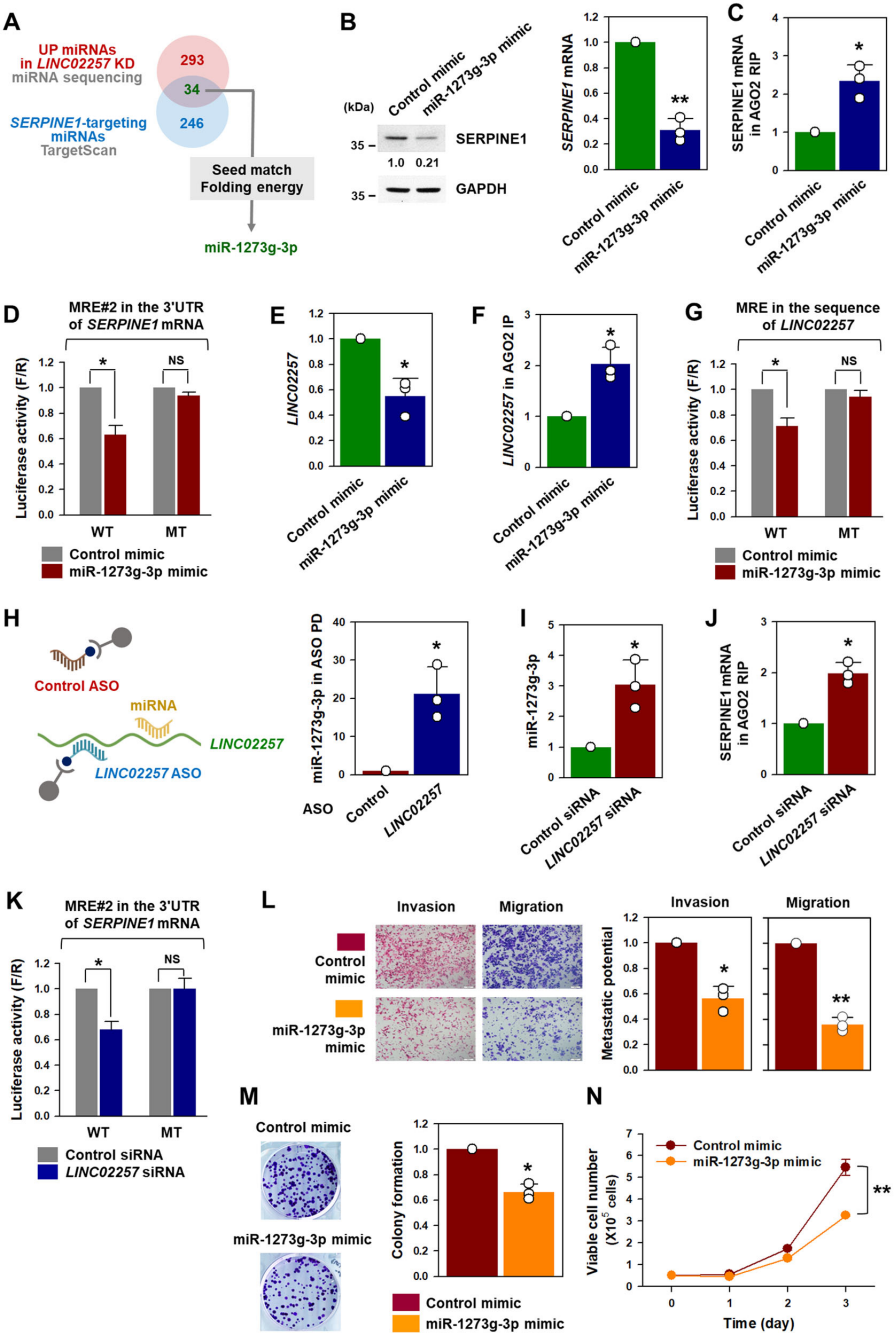
miR-1273g-3p, competitively interacting with *LINC02257*, suppresses malignant phenotypes in DLD1 cells. **A** miR-1273g-3p was identified as a *LINC02257*-associated miRNA by comparing the list of upregulated miRNAs by *LINC02257* knockdown and SERPINE1-targeting miRNAs. **B**, **C** DLD1 cells were transfected with a control or miR-1273g-3p mimic. Expression levels of SERPINE1 protein and mRNA were determined by western blot and RT-qPCR analyses, respectively (**B**). The enrichment of *SERPINE1* mRNA in miR-1273g-3p-loaded miRISC was determined using RT-qPCR (**C**). **D** The sequence-specific binding of miR-1273g-3p to the 3’UTR of *SERPINE1* mRNA was examined using luciferase vectors containing wild-type and mutated sequences of miR-1273g-3p MRE. **E**, **F** After DLD1 cells were transfected with a control or miR-1273g-3p mimic, the expression level of *LINC02257* was determined using RT-qPCR (**E**). Direct interaction between *LINC02257* and miR-1273g-3p was assessed by AGO2 RIP. The enrichment of *LINC02257* in miR-1273g-3p-loaded miRISC was determined using RT-qPCR (**F**). **G** Direct interaction between miR-1273g-3p and *LINC02257* was checked by luciferase assay using luciferase vectors containing wild-type and mutated sequences of miR-1273g-3p MRE in the sequence of *LINC02257*. **H** Antisense oligonucleotide (ASO) pulldown experiment was conducted to check the association of miR-1273g-3p with *LINC02257*. The level of miR-1273g-3p in the pulldown materials was determined using RT-qPCR. **I**–**K** To prove that *LINC02257* interacts with the miR-1273g-3p, DLD1 cells were transfected with control or *LINC02257*-targeting siRNA. The expression level of miR-1273g-3p was assessed by RT-qPCR (**I**). The enrichment was determined by measuring the level of *SERPINE1* mRNA in AGO2 IP (**J**). Luciferase assay was conducted using the vectors described in Fig. 6d (**K**). **L**–**N** DLD1 cells were transfected with a control or miR-1273g-3p mimic. Metastatic potential, including invasive and migratory abilities, was determined by the Transwell assay (**L**). Proliferative ability was assessed by colony forming assay (**M**) and by counting the number of viable cells (**N**). Data are expressed as mean ± standard deviation and the statistical significance was represented as follows: *p < 0.05, **p < 0.01.

Given that competitive endogenous RNA (ceRNA) is believed to be one of the main action mechanisms of lincRNAs, we searched for *LINC02257*-decoying miRNAs by comparing the lists of downregulated miRNAs obtained from miRNA sequencing using total RNA from *LINC02257*-silenced DLD1 cells and *SERPINE1*-targeting miRNAs using TargetScan (Fig. 6A and Supplementary Fig. S18D). Using RNA22 prediction algorithm analysis, 7 of the 34 miRNAs were predicted to bind to *LINC02257*. Because it exhibited the most optimal interaction (lowest folding energy), miR-1273g-3p was identified as the decoying miRNA of *LINC02257*. The sequences of *SERPINE1* mRNA and *LINC02257* contained two and one miRNA recognition element (MRE) for miR-1273g-3p, respectively (Supplementary Fig. S18E). To verify whether *SERPINE1* is a target of miR-1273g-3p, the protein and mRNA levels of SERPINE1 were assessed in DLD1 cells transfected with miRNA mimic. Western blot and RT-qPCR analyses revealed that miR-1273g-3p decreased SERPINE1 expression (Fig. 6B). In addition, the enrichment of *SERPINE1* mRNA in AGO2 IP was enhanced by miR-1273g-3p (Fig. 6C). We additionally examined the direct interaction between miR-1273g-3p and *SERPINE1* mRNA using a luciferase assay. Luciferase vectors containing wild-type or mutated sequences of miR-1273g-3p MREs in the 3’UTR of *SERPINE1* mRNA were constructed (Supplementary Fig. S19A, B). As previously observed, two miR-1273g-3p MREs in the 3’UTR of *SERPINE1* mRNA exist (Supplementary Fig. S18E). Of the two MREs, only MRE#2 exhibited an efficient binding of miR-1273g-3p to the 3’UTR of *SERPINE1* mRNA (Supplementary Fig. S19C). Overexpression of miR-1273g-3p inhibited the expression of luciferase containing only wild-type MRE (Fig. 6D), indicating that the decrease in SERPINE1 by miR-1273g-3p resulted from its direct binding to the 3’UTR of *SERPINE1* mRNA.

To examine whether *LINC02257* interacts with miR-1273g-3p, the level of *LINC02257* in DLD1 cells transfected with miRNA mimic was assessed using RT-qPCR. The overexpression of miR-1273g-3p decreased the level of *LINC02257* (Fig. 6E). Moreover, the RIP assay revealed that *LINC02257* was more enriched in AGO2 IP than in control IgG IP (Fig. 6F). Luciferase vectors containing the wild-type or mutated sequences of the miR-1273g-3p MREs in *LINC02257* were generated (Supplementary Fig. S19D). Overexpression of miR-1273g-3p inhibited the expression of luciferase (Fig. 6G) containing wild-type MRE, however, it did not affect luciferase activity in case of mutated vector. In addition, the direct interaction between *LINC02257* and miR-1273g-3p was verified using ASO pulldown assay (Fig. 6H). The level of *LINC02257* was higher in *LINC02257*-targeting ASO pulldown materials than in the control ASO. Next, we investigated whether *LINC02257* increases the level of miR-1273g-3p, thereby increasing the levels of *SERPINE1* mRNA in the AGO2 IP. Knockdown of *LINC02257* elevated the expression levels of miR-1273g-3p (Fig. 6I) and increased the interaction between *SERPINE1* mRNA and the miR-1273g-3p-mediated miRNA-induced silencing complex (miRISC) (Fig. 6J). Moreover, knockdown of *LINC02257* suppressed luciferase expression, indicating that *LINC02257* enhances the expression of SERPINE1 by interfering the interaction of miR-1273g-3p with the 3’UTR of *SERPINE1* mRNA (Fig. 6K). To verify the role of *LINC02257* as a ceRNA of miR-1273g-3p, we selected 138 genes by comparing the genes whose expression was downregulated upon *LINC02257* knockdown with the target mRNAs of miR-1273g-3p (Supplementary Fig. S20A). Among them, three genes (*SERPINE1*, *DGKI*, and *MEF2B*) closely related to the prognosis of patients with CRC were selected (Supplementary Fig. S20B, C) and the effects of *LINC02257* and miR-1273g-3p on their expression level was measured (Supplementary Fig. S20D). The expression levels of the target mRNAs (*DGKI* and *MEF2B*) were also downregulated by *LINC02257* knockdown and miR-1273g-3p overexpression. Conversely, an increase in *LINC02257* levels resulted in increased expression of *DGKI* and *MEF2B*.

Next, we examined whether miR-1273g-3p suppresses the malignant phenotypes of CRC. Overexpression of miR-1273g-3p reduced the number of invading and migrating DLD1 (Fig. 6L) and HCT116 cells (Supplementary Fig. S21A). It also inhibited the colony-forming ability and reduced the proliferation rate of both DLD1 (Fig. 6M, N) and HCT116 cells (Supplementary Fig. S21B, C). Based on these results, we concluded that *LINC02257* positively regulates malignant phenotypes by competitively mitigating *SERPINE1*-targeting miR-1273g-3p. Additionally, we tested the correlation of *LINC02257*, *SERPINE1*, and miR-1273g-3p. RT-qPCR results using the tissues of CRC patients and TCGA analyses revealed that there is a positive correlation between *LINC02257* and *SERPINE1* (*r* = 0.9035, *P* < 0.001) (Supplementary Fig. S22A, B). In stage 2 and 3 CRC tumor tissues, the expression levels of miR-1273g-3p were higher than those in normal tissues (Supplementary Fig. S22C) and negatively correlated with the expression levels of *LINC02257* (*r* = −0.8052, *P* < 0.001) (Supplementary Fig. S22D).

### *LINC02257* transactivated YB1 by inducing its phosphorylation

**Fig. 7:**
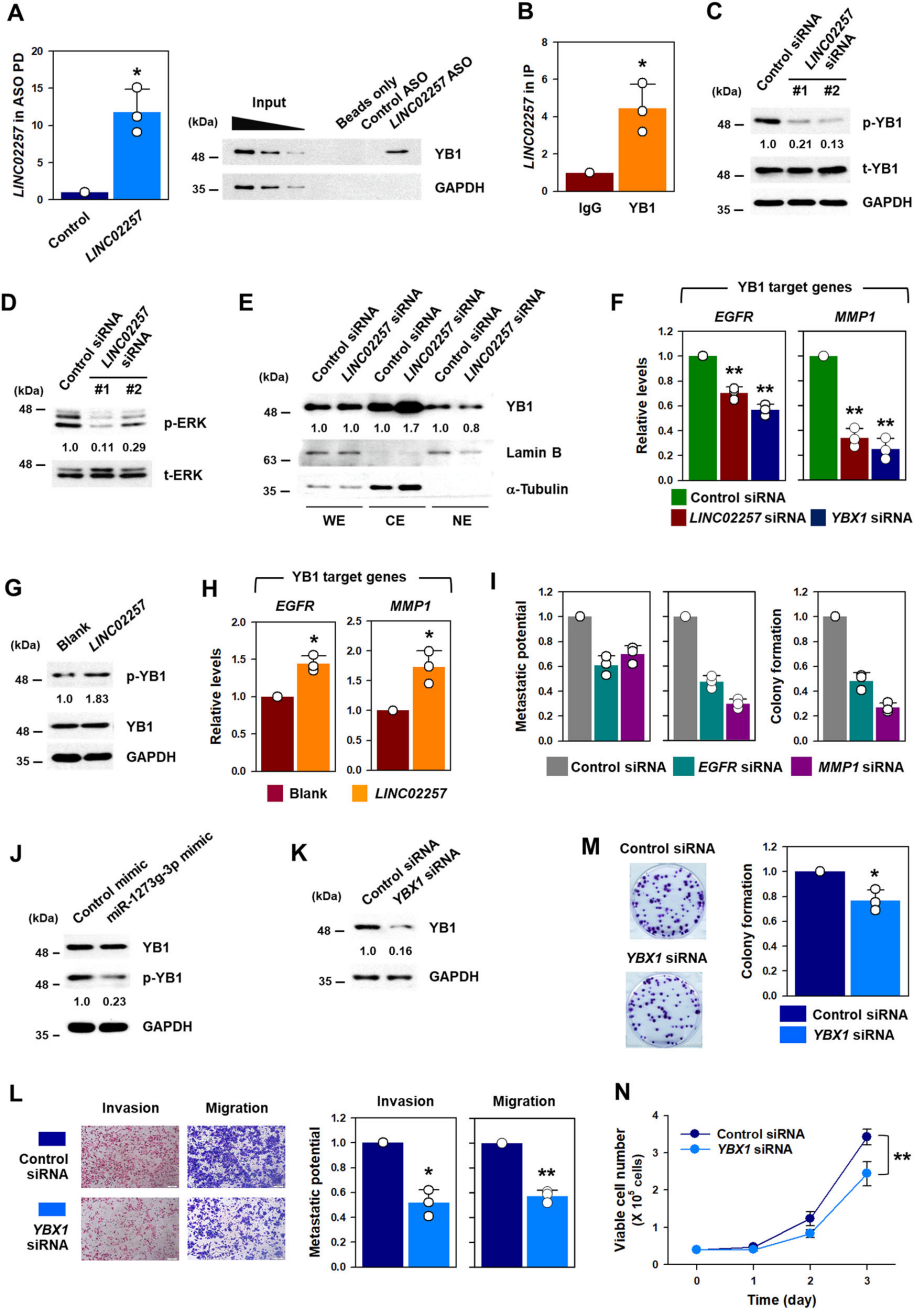
*LINC02257* is responsible for the phosphorylation of YB1 to regulate malignant phenotypes of CRC. **A** The efficiency of antisense oligonucleotide (ASO) pulldown was assessed by determining the level of *LINC02257* in the pulldown materials (left). The association of YB1 with *LINC02257* was determined by western blot (right). **B** RNA immunoprecipitation assay was conducted using a YB1-specific antibody and the level of *LINC02257* in IP materials was determined using RT-qPCR. **C**–**E** DLD1 cells were transfected with a control or *LINC02257*-targeting siRNA. The levels of phospho- and total YB1 were determined using western blot (**C**). The level of GAPDH was assessed as loading control. The activation of ERK was assessed by western blot using phospho-ERK antibody (**D**). The level of total ERK was checked for loading control. **E** Cellular localization of YB1 was examined by fractionation assay. Lamin B and GAPDH protein were determined using western blot to prove that fractionation was appropriately done. **F** The expression of YB1 target genes was determined by RT-qPCR in *LINC02257*- or *YBX1*-silenced DLD1 cells. **G**, **H** Following the overexpression of *LINC02257*, the level of phosphorylated and total YB1 was determined by Western blot (**G**) and the expression of YB1 target genes was determined by RT-qPCR (**H**). **I** The effects of knockdown of YB1 target genes on oncogenic phenotypes of DD1 cells were examined by Transwell and colony forming assays. **J** The effect of miR-1273g-3p on the phosphorylation of YB1 was examined by Western blot. **K**–**N** DLD1 cells were transfected with control or *YBX1*-targeting siRNA. Expression levels of the YB1 protein were determined using western blot (**K**). Metastatic potential including invasive and migratory abilities was determined by the Transwell assay (**L**). Proliferative ability was assessed by colony forming assay (**L**) and by counting the number of viable cells (**N**). Data are expressed as mean ± standard deviation and the statistical significance was represented as follows: *p < 0.05, **p < 0.01.

To search for *LINC02257*-associated RNA-binding proteins (RBPs), proteins bound to *LINC02257* were isolated by ASO pulldown, separated by SDS-PAGE, and analyzed by Nanospray LC/MS/MS (Supplementary Fig. S22). Three RBPs, Y-box binding protein 1 (YB1), YB3, and heterogeneous nuclear ribonucleoprotein C (HNRNPC), showed significant interactions with *LINC02257*. Among these, YB1, encoded by *YBX1*, was selected because it exhibited the highest binding score. To verify the interaction between *LINC02257* and YB1, an ASO pulldown assay was performed. Prior to examining the binding of YB1 to *LINC02257*, the pulldown efficiency was examined by measuring the level of *LINC02257* in ASO pulldown materials (Fig. 7A). RT-qPCR results showed that the ASO designed for *LINC02257* was efficient. An ASO pulldown performed followed by western blot analysis, verified the direct interaction between *LINC02257* and YB1. The association between YB1 and *LINC02257* was examined using YB1 RIP experiments (Fig. 7B). The level of *LINC02257* was higher in YB1 IP materials than in control IP. Based on these results, we concluded that YB1 is a *LINC02257*-associate RBP.

We assessed YB1 expression in *LINC02257*-silenced DLD1 cells. Notably, knockdown of *LINC02257* affected the level of phosphorylated YB1 without altering the total YB1 (Fig. 7C). In addition, the activation of ERK, a major cellular signal for YB1 phosphorylation, was inhibited by knockdown of *LINC02257* (Fig. 7D). These results indicated that *LINC02257* is responsible for YB1 phosphorylation via ERK. Given that the phosphorylation of YB1 is closely implicated in its function and localization, a cellular fractionation experiment was conducted (Fig. 7E). Compared to the control, the cytosolic level of YB1 was higher in *LINC02257*-silened cells. In contrast, YB1 expression in the nucleus was reduced by knockdown of *LINC02257*. These results were also observed by immunofluorescence (IF) staining experiments (Supplementary Fig. S24). To verify whether *LINC02257* regulates the localization of YB1, the expression levels of YB1 target genes were examined (Fig. 7F). All three YB1 target genes tested (*EGFR* and *MMP1*) were downregulated by knockdown of *LINC02257* or *YBX1*. In contrast, overexpression of *LINC02257* increased the level of phosphorylated YB1 (Fig. 7G) and the expression of its target genes (Fig. 7H). We tested whether YB1 targets including *EGFR* and *MMP1* are involved in the control of proliferative and metastatic abilities in DLD1 cells. DLD1 cells transfected with *EGFR*- or *MMP1*-specific siRNA exhibited weakened metastatic potential including migratory and invasive abilities (Fig. 7I and Supplementary Fig. S25A, B). In addition, knockdown of both YB1 targets induced retardation of cell growth and inhibited colony forming ability (Supplementary Fig. S25C). These results demonstrate that YB1-mediated gene regulation is responsible for the oncogenic function of *LINC02257*. In addition, miR-1273g-3p hindered the phosphorylation of YB1, which resulted from the decreased *LINC02257* expression (Fig. 7J).

To determine whether YB1 is involved in the malignant phenotypes of CRC, the metastatic and proliferative abilities of *YBX1*-silenced cells were measured. The *YBX1*-specific siRNA used in this study efficiently suppressed YB1 expression (Fig. 7K). Knockdown of YB1 suppressed the metastatic potential, including invasive and migratory abilities (Fig. 7L), reduced the number of colonies (Fig. 7M), and slowed the proliferation rate (Fig. 7N) in DLD1 cells. Similar results were observed in the *YBX1*-silenced HCT116 cells (Supplementary Fig. S26). These results indicate that *LINC02257* regulates the localization of YB1 through its phosphorylation and that the transactivation of YB1 plays a significant role in regulating the malignancy of CRC by *LINC02257*.

## Discussion

Although lncRNAs play an important role in cancer progression, only a few can be used to treat patients with CRC. It is difficult to identify the causal lncRNAs because their functions are governed by complex interactions with other genes. Therefore, we systematically investigated the TCGA transcriptome data to identify lncRNAs associated with CRC malignancy. Among the DE lncRNAs discovered here, dysregulation of *LINC02418* [6], AFAP1-AS1 [28], BLACAT1 [29], and MIR31HG [30] has been linked to the growth and progression of CRC (Supplementary Table S4). However, we prioritized *LINC02257* as a colon cancer-associated lncRNA because it is over-expressed in primary colon tumors compared to normal tissues, and its expression levels correlate with poor survival and MSI in patients with CRC. MSI is caused by an abnormality in mismatch repair that occurs during DNA replication, resulting in the accumulation of mutations. Recently, the mutation burden has been shown to substantially increase in CRC compared with normal cells [31], and was significantly associated with survival outcomes and response to immune therapy [25, 32].

Emerging evidence indicates that lncRNAs function as powerful regulators of gene expression. The mechanism by which lncRNAs regulate gene expression involves both transcriptional and post-transcriptional processes. Recently, the function of lncRNAs as ceRNAs has been revealed, attracting the attention of many scientists [33]. Generally, ceRNAs mitigate the inhibitory function of miRNAs by competitively binding to target mRNA. Imbalances in the ceRNA network have been identified in various cancers and are closely implicated in cancer malignancy [34]. To data, numerous ceRNAs associated with tumorigenesis and metastasis of CRC have been reported in CRC. *LINC00342* promotes the proliferation and metastasis of CRC by inhibiting the miR-19a-3p/NPEPL1 axis [35]. *LINC01296* enhances CRC metastasis by sponging miR-141-3p and accelerating EMT [36]. *LINC00473* facilitates CRC cell proliferation and invasion by targeting miR-195 [37]. *LINC01569* promotes the proliferation and metastasis of CRC via the miR-381-3p/RAP2A axis [38]. We also discovered that the tumor-suppressive miR-1273g-3p interacts with *LINC02257* as a decoying miRNA, thereby regulating SERPINE1 expression. The inhibition of CRC proliferation by miR-1273g-3p results from the activation of AMPK signaling by suppressing MAGEA3/6 [39]. Several lncRNAs function as ceRNAs for miR-1273g-3p and exhibit oncogenic effects in various cancers. In glioma and glioma-stem-like cells, lncRNA RP11-279C4.1 enhances malignant phenotypes by targeting the miR-1273g-3p/CBX3 axis [40]. *LINC02418* regulates MELK expression by absorbing miR-1273g-3p and serves as a diagnostic marker for CRC [41]. SNHG3 lncRNA is responsible for bone metastasis in breast cancer through regulation of the miR-1273g-3p/BMP3 axis [42].

In addition to acting as miRNA sponges, lncRNAs influence the function of RBP by acting as ceRNAs. In this study, we identified YB1 as a LINC02257-associated RBP. YB1 encoded by *YBX1*, is a family of RBP containing an evolutionally conserved cold-shock domain [43]. It is widely recognized as regulator of gene expression processes such as transcription, splicing, and translation. YB1 is primarily localized in the cytoplasm, but upon phosphorylation at S102, it can translocate to the nucleus [44]. High YB1 expression is associated with local recurrence and a poor prognosis of patients with CRC [45]. Moreover, the inhibition of YB1 suppresses the growth and motility of CRC [46]. Several lncRNAs serve as ceRNAs for YB1, which regulate its stability and localization. HIF1A-AS1 promotes the gemcitabine resistance of pancreatic cancer by translationally upregulating HIF1α and facilitating the interaction of YB1 to Akt, inducing the phosphorylation of YB1 [47]. Myc-activated lncRNA MNX1-AS1 promotes the CRC progression by directly binding to and stabilizing YB1 [48]. By interacting with YB1, MIR200HG enhances proliferation, invasion, and drug resistance [49]. In breast cancer, the lncRNA AC073352.1 increases metastasis and angiogenesis by interacting with YB1 [50]. The lncRNA HULC modulates the phosphorylation of YB1 by serving as a ERK scaffold protein, thereby enabling the release of translational suppression of its bound mRNAs such as cyclin D1, cyclin E1, and matrix metalloproteinase 3 [51]. Our results revealed that *LINC02257* facilitates the phosphorylation of YB1 via ERK and induces the nuclear translocation of YB1, where it transcriptionally upregulates its target genes such as *EGFR*, *CCNA1*, and *MMP1*.

Molecular analysis revealed that *LINC02257* controls CRC malignancy via miR-1273g-3p/*SERPINE1* and YB1. Furthermore, bioinformatic analyses indicated that *LINC02257* plays an essential role in EMT during cancer progression. EMT is a process by which epithelial cells lose their polarity and adhesion, resulting in the acquisition of migratory and invasive abilities [52]. The ceRNA network is a well-known regulator of the EMT in CRC [53]. *SERPINE1*, a target gene of *LINC02257*/miR-1273g-3p, is associated with EMT and facilitates cell proliferation, migration, and invasion [6, 52, 54]. It is also involved in remodeling of the tumor microenvironment during CRC progression [55]. In addition to *SERPINE1*, YB1 is a downstream molecule responsible for the oncogenic function of *LINC02257*. The expression and phosphorylation of YB1 impact the EMT process through various mechanisms [56, 57]. At the post-transcriptional level, YB1 regulates the EMT process by triggering cap-independent translation of EMT-promoting genes and suppressing cap-dependent translation of growth-promoting genes [58]. Additionally, YB1 upregulates ZEB1 expression by inhibiting the maturation of *ZEB1*-targeting miR-205/200b, which in turn facilitates EMT [59]. Natural low-molecular compounds exhibiting anti-proliferative and anti-metastatic effects are also being studied as promising targets for the treatments for colon cancer [60–62]. In addition, the gut microbiome has recently been discovered to play an important role in the carcinogenesis and malignancy of gastrointestinal cancer, emerging as a new paradigm for the treatment of colon cancer [63].

**Fig. 8:**
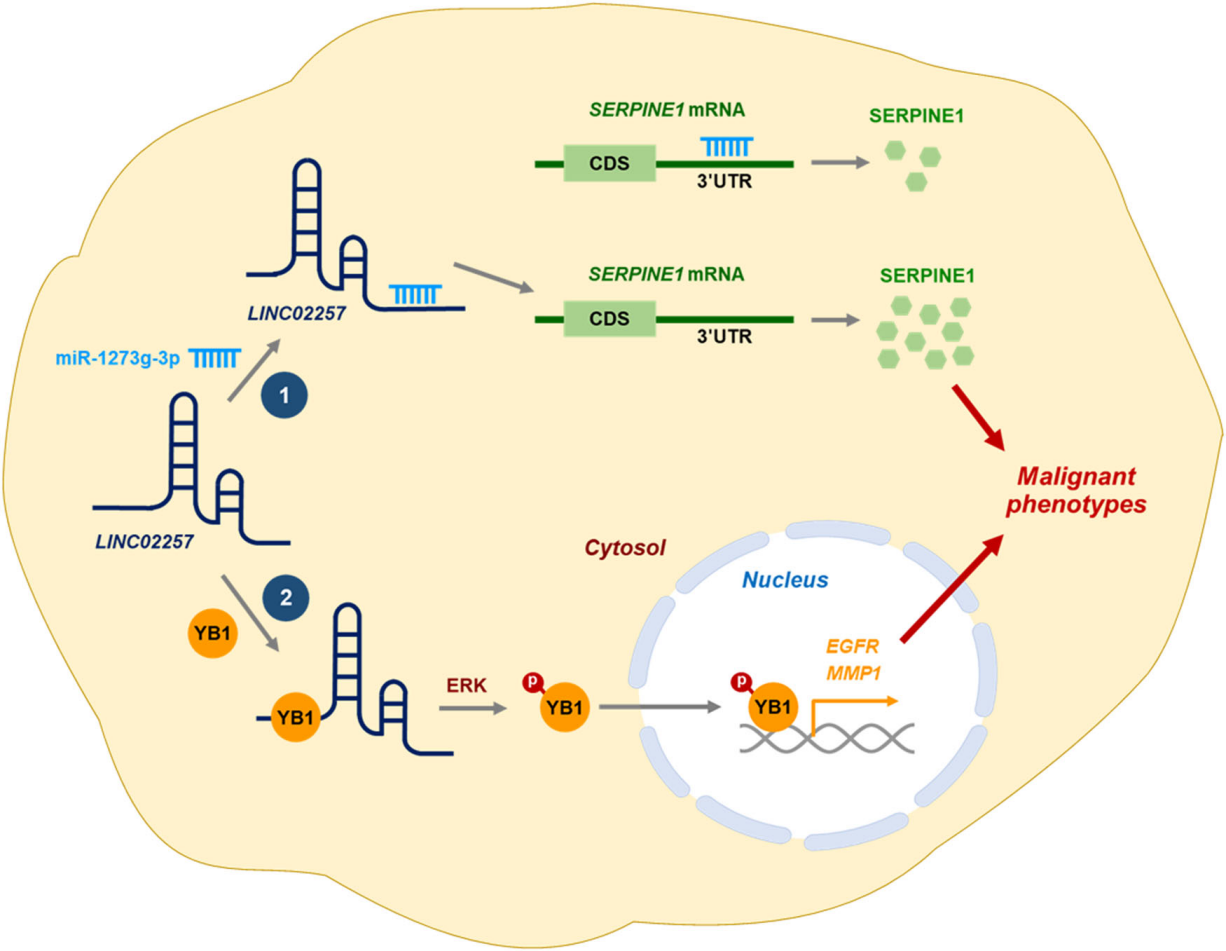
Schematic illustration demonstrating the role of *LINC02257* in controlling malignant phenotypes of CRC by interacting with miR-1273g-3p and YB1. Details in the main text.

Our findings are summarized in Fig. 8. The comprehensive multilevel bioinformatics indicates *LINC02257* is highly expressed in CRC and its higher expression is closely associated with a poor prognosis in patients with CRC, suggesting that *LINC02257* is a potential causal lncRNA in CRC. Interestingly, the clinical significance of *LINC02257* can be found in MSI (approximately 15% of all CRC) rather than MSS, which accounts for the majority of colon cancer. Compared to the expression level of *LINC02257* in MSS CRC, it is specifically high in MSI CRC and is more closely related to poor prognosis in patients with MSI CRC. Because MSI CRC exhibits a good responsiveness to immunotherapy, *LINC02257* as an MSI-specific lncRNA, might be a promising biomarker for the development of treatments and diagnostics for CRC immunotherapy. Knockdown of *LINC02257* suppressed invasive and migratory abilities and delayed the proliferation of CRC cells. Conversely, its overexpression strengthens the metastatic potential and enhances proliferation. SERPINE1 was identified as a target of *LINC02257*. Mechanistically, *LINC02257* upregulates SERPINE1 by sequestering miR-1273g-3p. In addition, *LINC02257* regulates the nuclear translocation of YB1 by direct interacting and phosphorylating YB1, controlling the metastatic and proliferative abilities by *LINC02257*. Taken together, we demonstrate that *LINC02257* is a promising therapeutic target for CRC treatment.

## Supplementary information

Supplementary Methods

Supplementary Figures

Supplementary Tables

Uncropped WB images

## Data Availability

Bulk and single RNA data used in this study have been published previously and are available in the Gene Expression Omnibus (GSE146771 and GSE146009). Transcriptome and clinical data from TCGA have been previously published and are available in the Genome Data Commons Data Portal, however, genotype data have been approved by the Database of Genotypes and Phenotypes (dbGaP). The Genotype-Tissue Expression (GTEx) Project was supported by the Common Fund of the Office of the Director of the National Institutes of Health and by the NCI, NHGRI, NHLBI, NIDA, NIMH, and NINDS.

## Code availability

The source codes for this study are available from the corresponding author upon reasonable request.
