# Supplementary material for: LINC02257 regulates malignant phenotypes of colorectal cancer via interacting with miR-1273g-3p and YB1
Source: Cell Death Dis. 2024 Dec 18;15(12):895. doi: 10.1038/s41419-024-07259-4 (PMC11655847; doi:10.1038/s41419-024-07259-4)
Supplement: Supplementary file 1 — Supplementary Methods [file 41419_2024_7259_MOESM1_ESM.docx]

**Supplementary Methods**

**1. Bioinformatic approaches**

**Replication study for the results from the analyses of TCGA colon cancer**

To compare the expression patterns of lncRNA, we collected fifteen cancer datasets from TCGA including digestive organs. We obtained 149 (140 case and 9 control samples) esophageal carcinoma, 375 (345 case and 30 control samples) stomach adenocarcinoma, 388 (346 case and 42 control samples) liver hepatocellular carcinoma, 178 (174 case and 4 control samples) pancreatic adenocarcinoma, 163 (153 case and 10 control samples) rectum adenocarcinoma, 599 (527 case and 72 control samples) kidney renal clear cell carcinoma, 290 (258 case and 32 control samples) kidney renal papillary cell carcinoma, 1188 (1076 case and 112 control samples) breast invasive carcinoma, 300 (297 case and 3 control samples) cervical squamous cell carcinoma and endocervical adenocarcinoma, 564 (541 case and 23 control samples) uterine corpus endometrial carcinoma, 554 (496 case and 58 control samples) lung adenocarcinoma, 540 (491 case and 49 control samples) lung squamous cell carcinoma, 474 (431 case and 43 control samples) head and neck squamous cell carcinoma ,and 424 (405 case and 19 control samples) bladder urothelial carcinoma (Supplementary Table 2).

Additionally, we acquired GEO dataset, GSE146009 that has 30 (12 case and 15 control samples) African Americans and 35 (18 case and 17 control samples) Caucasian Americans in colon cancer (Supplementary Table 2). This dataset was stratified with 33 tumor and 32 non-tumor adjacent tissues for different populations, African Americans and Caucasian Americans colon cancer patients. We separated population and analyzed tumor and normal tissues, respectively, and present the expression pattern.

**Association study between SNPs nearby identified two lncRNAs and colorectal cancer**

We obtained publicly available GWAS summary statistics data of colorectal cancer to examine whether SNPs nearby LINC02257 and LINC01836 were associated with colorectal cancer risk. Data was from the GWAS of colorectal cancer in East Asian population which was conducted in 6,692 cases and 27,178 controls, followed by a replication analysis using more than 11,000 case-control samples ^1^. All DNA were extracted from peripheral blood leukocytes and normal tissues of CRC patients and healthy controls. GWAS of colorectal cancer with 4,562 cases and 382,756 controls used SAIGE method to solve case-control imbalance from European population ^2^.

We selected SNPs nearby the two identified lncRNAs (±1 mega base pairs) to identify SNPs associated with colon cancer. The association results were visualized by LocusZoom ^3^. To confirm SNPs as candidate cis-eQTL in colon tissue, we examined data from the GTEx database (v8). For each locus, we identified with reported eQTLs in all tissues (Supplementary Fig. 4-5).

**eQTL mapping and predicted prognosis using genotype**

We download the TCGA genotype data to verified association with LINC02257. Genotype data need to request access permission from database of Genotypes and Phenotypes (dbGaP) (phs000178.v11). We used 460 genotype data and matched 456 transcripts per million (TPM) normalized expression data of colon cancer from TCGA. To removed confounding factor, PEER was performed using sex and age as covariates after excluding genes with read counts < 10. Subsequent analysis was performed with the corrected expression data from PEER. We used a multiple linear regression model in FastQTL to detect cis-eQTL as a variant within 1 Mb of the genes ^4^.

**WGCNA (weighted gene co-expression network analysis)**

To identify co-expressed genes with LINC02257, we performed the WGCNA (v1.68) R package using TCGA expression data which is the analysis assumed that genes with similar functions have similar expression patterns. For network construction, we used 48,557 genes which passing the quality control as removed sum of read counts for each gene less than 10 and gene types was uncorrected. DE genes were selected when the genes were |LFC| ≥ 1 and adjusted P < 0.05. A total of 12,141 genes remained for the network construction (Supplementary Fig. 7).

Nodes in the network correspond to gene expression profiles, and edges were calculated by the pairwise correlations between expressions of genes. For co-expression network construction, Pearson’s correlation was used to create adjacency matrix, and power value was set at 5 using the pickSoftThreshold function (Supplementary Fig. 7). The power value was based on the scale-free topology standard ^5^, resulting in a scale-free topology index (R²) was 0.92. The cutreeDynamic was used with the following parameters: cutHeight = 0.998, deepSplit = 4, pamStage = T, and pamRespectsDendro = F. We converted an adjacency matrix to a topological overlap matrix (TOM), that measures the network connectivity of genes defined as the sum of adjacent genes. Average linkage hierarchical clustering was performed based on TOM-based dissimilarity measurements, and we calculated dissimilarity among modules and constructed module dendrograms. The genes connected to *LINC02257* by edges were visualized network plot was using Cytoscape (v3.9.1) ^6^.

**Functional analysis**

For the annotation of putative risk genes in colon cancer, we conducted pathway analysis using WebGestalt (Web-based GEne SeT AnaLysis Toolkit) ^7^. An enrichment pathway analysis was used with the following parameters: specific organism = H. sapiens, enrichment categories = KEGG and Panther pathway, minimum number = 5, and maximum number of ID in each category = 2000. A reference list was used for all mapped gene symbols from the selected platform ‘genome’. GO (Gene ontology) analysis conducted via g:Profiler ^8^, and we was follow default parameters.

**Cell type definition**

CIBERSORTx was applied to infer cell-type specific gene expression profiles without physical cell isolation ^9^. We prepared the signature matrix and mixture datasets of tumor and normal groups obtained from GSE1467711 ^10^ and TCGA, according to the instructions provided by CIBERSORTx. The GSE146771 dataset comprised tumor, adjacent normal tissue, and blood from 18 colorectal cancer patients, and is globally clustered into the following 8 cell types: CD4+ T cells, CD8+ T cells, B cells, ILC, myeloid cells, epithelial, fibroblast, and malignant cells. We aligned the signature matrix and mixture file to the same normalization space. The scRNA data were derived from 10x and Smart-seq2, and “S-mode” was chosen for batch correction due to the high proportion of 10x data. We set parameters retained the default. After running CIBERSORTx, we acquired the relative proportions of the 8 global cell types in each sample with adjusted P < 0.01 for the deconvolution. Then, within each TCGA samples, we executed a Spearman’s correlation test between gene expression and each cell types estimated proportion.

**Abbreviations**

BLCA, bladder urothelial carcinoma; BP, biological process; BRCA, breast invasive carcinoma; CC, cellular component; CESC, cervical squamous cell carcinoma and endocervical adenocarcinoma; CI, confidence interval; COAD, colon adenocarcinoma; dbGaP, database of genotypes and phenotypes; DE, differentially expressed; ECM, extracellular matrix; EMT, epithelial–mesenchymal transition; eQTL, expression quantitative trait; ESCA, esophageal carcinoma; GEO, gene expression omnibus; GO, gene ontology; GTEx, genotype-tissue expression; GWAS, genome-wide association study; HNSC, head and neck squamous cell carcinoma; HR, hazard ratio; ILCs, innate lymphoid cells; KIRC, kidney renal clear cell carcinoma; KIRP, kidney renal papillary cell carcinoma; LD, linkage disequilibrium; LFC, log2 fold change; LIHC, Liver hepatocellular carcinoma; lncRNA, long non coding ribonucleic acid; LUAD, lung adenocarcinoma; LUSC, lung squamous cell carcinoma; Mb, mega-base pairs; MF, molecular function; MSI, microsatellite instability; OS, overall survival; PAAD, Pancreatic adenocarcinoma; READ, Rectum adenocarcinoma; RNA-seq, ribonucleic acid sequencing; SNP, single nucleotide polymorphism; STAD, stomach adenocarcinoma; TCGA, the cancer genome atlas; TPM, transcripts per million; UCEC, uterine corpus endometrial carcinoma; WebGestalt, web-based gene set analysis toolkit; WGCNA, weighted gene co-expression network analysis

**Web reference**

Bioconductor, https://www.bioconductor.org/packages/release/bioc/html/rhdf5.html

CIBERSORTx, https://cibersortx.stanford.edu/

Cytoscape, https://cytoscape.org/

Ensembl, https://asia.ensembl.org/index.html

GEO database, https://www.ncbi.nlm.nih.gov/geo/

g:Profiler, https://biit.cs.ut.ee/gprofiler/

GTEx, https://gtexportal.org/home/

TCGA Portal, https://cancergenome.nih.gov

TCGA Data Portal, https://portal.gdc.cancer.gov

WebGestalt, http://www.webgestalt.org/

**2. Molecular biological approaches**

**Cell culture and transfection**

CRC cells (DLD1, HCT116, RKO, and HT29) were obtained from the Korean Cell Line Bank and cultured with Dulbecco’s modified Eagle’s medium (Gibco, Waltham, MA, USA) containing 10% fetal bovine serum. All cell lines were free of mycoplasma contamination and verified by short tandem repeat analysis. Lipofectamine 2000 (Invitrogen, Carlsbad, CA, USA) was used to introduce siRNA, miRNA, or plasmids into CRC cells according to the manufacturer’s protocol. Sequences of siRNA used in this report were in Supplementary Table 3.

**Western blot analysis**

Whole cell lysates (WCL) were prepared using radioimmunoprecipitation (RIPA) buffer containing protease and phosphatase inhibitors (Roche, Basel, Switzerland). The equal amounts of WCL were fractionated by sodium dodecyl sulfate-polyacrylamide gel electrophoresis (SDS-PAGE) and transferred to polyvinylidene difluoride membranes (Millipore, Billerica, MA, USA). The membranes were blocked, incubated with indicated primary antibodies (Supplementary Table 4) overnight, washed, and incubated with appropriate secondary antibodies. The bands were visualized using an enhanced chemiluminescence reagent. The intensity of the bands was measured using the ImageJ program, and GAPDH was used as a loading control.

**Reverse transcription-quantitative polymerase chain reaction (RT-qPCR) analysis**

Using RNA as a template, cDNA was synthesized using the SuperScript III First-Strand Synthesis System (Invitrogen). The mRNA levels were assessed by RT-qPCR with appropriate primers (Supplementary Table 5) using the Power SYBR Green PCR Master Mix (Applied Biosystems, Foster City, CA, USA). The level of miR-1273g-3p was assessed using a specific TaqMan primer (Applied Biosystems).

**Determination of malignant phenotypes**

Metastatic potential including invasive and migratory abilities was determined using the BD Biocoat™ Matrigel invasion chamber (BD Bioscience, San Jose, CA, USA) and Transwell® permeable supports (Corning, Rockville, MD, USA), respectively. Equal number of cells in serum-free media was added into the upper chamber. Both invasion and migration were initiated by adding the same media containing 10% serum (as a chemoattractant) into the lower chamber. Twenty-four h after cell seeding, the invaded/migrated cells on the lower surface of the membrane were fixed with 95% MeOH and stained with 0.1% hematoxylin and eosin. Invasive and migratory abilities were calculated by counting the number of cells in randomly selected eight fields.

Cell proliferation was assessed by colony formation assay and cell counting. For colony formation assay, cells were plated into 6-well plates and incubated for 2 weeks. The number of colonies was counted after cells were fixed and stained with 0.2% crystal violet. To compare proliferation rate, the equal number of cells were pated into 6-well plates and the number of viable cells was counted every 24 h under a microscope.

**Subcutaneous injection mouse model**

Long-term effect of *LINC02257* in vivo was examined in the subcutaneous injection model. The equal number of transfected DLD1 cells (1 × 10^6^ cells) were resuspended in 50 μl of Hanks' Balanced Salt Solution), mixed with 50 μl of Matrigel, and then injected subcutaneously into the flanks of 6- to 7-week-old female BALB/c nude mice (6 mice per group, which is required to measure the statistical significance) (Orient Bio Group, Seoul, Korea). Tumor size was measured using a caliper, and tumor volume was calculated using the following formula: (short length × long length × width) / 2. The measurement of tumor size and volume was performed by three expertized researchers not through a randomization method. No animals were excluded from the analysis. The mice were euthanized 3–4 weeks after inoculation and tumors were excised. All animal experiments were conducted at a specific pathogen-free animal experiment center at the Samsung Medical Center. The Samsung Medical Center on Laboratory Animals Committee approved the experiments (approval number: 2017-07-131-202).

**Cellular fractionation**

To examine the localization of *LINC02257*, cellular fractionation assay was performed described in a previous report ^11^. The cytosolic lysates were prepared using RSB buffer (10 mM Tris-HCl, pH 7.4, 2.5 mM MgCl_2_, 100 mM NaCl) containing 4 mg/ml digitonin (Thermo Fisher Scientific, Waltham, MA USA). After centrifugation, the pellets were lysed with RIPA buffer to collect nuclear lysates. The levels of α-tubulin and lamin B were assessed to verify the cytosolic and nuclear lysates, respectively.

**Ribonucleoprotein immunoprecipitation (RIP)**

Argonaute 2 (AGO2) RIP was performed to check whether miR-1273g-3p binds to *LINC02257* and *SERPINE1* mRNA. After overexpression of miR-1273g-3p in DLD1 cells, immunoprecipitation (IP) was performed using AGO2 antibodies. The levels of *LINC02257* and *SERPINE1* mRNA in AGO2 IP were measured using RT-qPCR.

To examine the interaction between YB1 and *LINC02257*, RIP assay was performed as described in a previous report ^11^. Briefly, the Dynabeads® Protein G (Thermo Fisher Scientific) was coated with IgG or YB1 antibody, and incubated with the equal amounts of cytoplasmic lysate for 4 h. After washing several times with NT2 buffer, the beads were treated with DNase I (Ambion, Austin, TX) and protease K (Bioneer, Daejeon, Republic of Korea), and RNA was isolated by precipitation with absolute ethanol. The level of *LINC02257* in YB1 IP was determined by RT-qPCR.

**Antisense oligonucleotide (ASO) pulldown assay**

ASOs recognizing *LINC02257* were designed and used to identify *LINC02257*-associated miRNAs and RNA-binding proteins (RBPs). Briefly, the cytoplasmic lysates were prepared using polysome extraction buffer and incubated with biotinylated ASOs at 4°C for 2 h. LacZ ASO was used as a negative pulldown control. After following incubation with pre-washed streptavidin-coupled Dynabeads^TM^ (Invitrogen), the RNA was isolated from the pulldown materials using Trizol and RT-qPCR was conducted to measure the level of miR-1273g-3p. To validate the interaction of *LINC02257* with YB1, the proteins were separated by SDS-PAGE, digested, and analyzed by Nanospray LC/MS/MS analysis.

**Luciferase reporter assay**

The luciferase vectors (pmirGLO dual-luciferase vectors; Promega, Madison, WI) containing wild-type or mutated sequences of miR-1273g-3p MRE in *LINC02257* or the 3'UTR of *SERPINE1* mRNA were constructed (detail information in Supplemental Fig. 6). Briefly, after DLD1 cells were transfected with control or miR-1273g-3p mimic, the equal number of cells were resuspended into 24-well plates. Following transfection with either wild-type or mutant luciferase vectors, luciferase activity was determined using a Dual-GLO™ Luciferase Assay System (Promega).

**Clinical specimens**

All tissues were collected from CRC patients who had undergone surgery at Samsung Medical Center (Seoul, Korea). All primary normal and CRC tumor tissues (six pairs of stage 2 CRC, four pairs of stage 3 CRC, and two stage 4 CRC) and were obtained from surgical resections of CRC patients without any radiotherapy or chemotherapy before surgery. The tissues were pathologically validated and stored in liquid nitrogen until use. All human specimens were approved by the Institutional Review Board of the Samsung Medical Center (IRB approval No. 2021-09-112) with the written informed consent from all patients.

**Immunofluorescence staining**

DLD1 cells were transfected with *LINC02257* siRNA or overexpression vector, and the equal number of transfected cells was resuspended on cover glass. Cells were fixed with 4% paraformaldehyde for 10 min and permeabilized with 0.1% Triton X-100 for 5 min. After blocking with 1% BSA for 20 min, cells were incubated with anti-YB1 antibody (1:100), washed, and incubated with secondary antibody (Alexa Flour™ 488 Goat anti-rabbit IgG), Nuclei were stained with DAPI.

**REFERENCES**

1. Tanikawa, C.*, et al.* GWAS identifies two novel colorectal cancer loci at 16q24. 1 and 20q13. 12. *Carcinogenesis* **39**, 652-660 (2018).

2. Zhou, W.*, et al.* Efficiently controlling for case-control imbalance and sample relatedness in large-scale genetic association studies. *Nature genetics* **50**, 1335-1341 (2018).

3. Pruim, R.J.*, et al.* LocusZoom: regional visualization of genome-wide association scan results. *Bioinformatics* **26**, 2336-2337 (2010).

4. Ongen, H., Buil, A., Brown, A.A., Dermitzakis, E.T. & Delaneau, O. Fast and efficient QTL mapper for thousands of molecular phenotypes. *Bioinformatics* **32**, 1479-1485 (2016).

5. Zhang, B. & Horvath, S. A general framework for weighted gene co-expression network analysis. *Statistical applications in genetics and molecular biology* **4**(2005).

6. Shannon, P.*, et al.* Cytoscape: a software environment for integrated models of biomolecular interaction networks. *Genome research* **13**, 2498-2504 (2003).

7. Liao, Y., Wang, J., Jaehnig, E.J., Shi, Z. & Zhang, B. WebGestalt 2019: gene set analysis toolkit with revamped UIs and APIs. *Nucleic acids research* **47**, W199-W205 (2019).

8. Raudvere, U.*, et al.* g: Profiler: a web server for functional enrichment analysis and conversions of gene lists (2019 update). *Nucleic acids research* **47**, W191-W198 (2019).

9. Newman, A.M.*, et al.* Determining cell type abundance and expression from bulk tissues with digital cytometry. *Nature biotechnology* **37**, 773-782 (2019).

10. Zhang, L.*, et al.* Single-cell analyses inform mechanisms of myeloid-targeted therapies in colon cancer. *Cell* **181**, 442-459. e429 (2020).

11. Ji, H.*, et al.* Two circPPFIA1s negatively regulate liver metastasis of colon cancer via miR-155-5p/CDX1 and HuR/RAB36. *Mol Cancer* **21**, 197 (2022).
