# Supplementary material for: LINC02257 regulates malignant phenotypes of colorectal cancer via interacting with miR-1273g-3p and YB1
Source: Cell Death Dis. 2024 Dec 18;15(12):895. doi: 10.1038/s41419-024-07259-4 (PMC11655847; doi:10.1038/s41419-024-07259-4)
Supplement: Supplementary file 2 — Supplementary Figures [file 41419_2024_7259_MOESM2_ESM.pptx]

## Slide 1
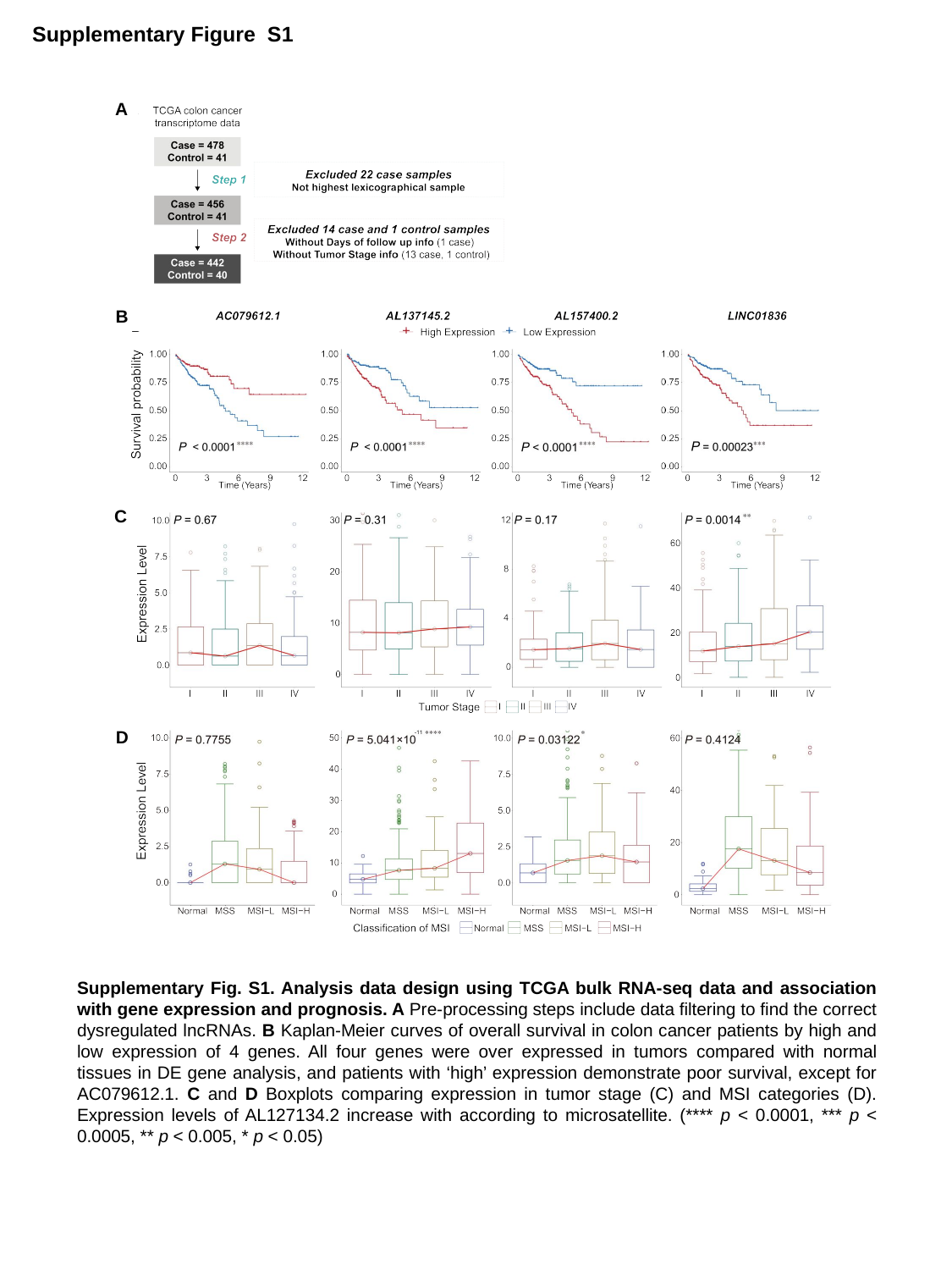

Supplementary Figure S1
A
B
C
D
Supplementary Fig. S1. Analysis data design using TCGA bulk RNA-seq data and association with gene expression and prognosis. A Pre-processing steps include data filtering to find the correct dysregulated lncRNAs. B Kaplan-Meier curves of overall survival in colon cancer patients by high and low expression of 4 genes. All four genes were over expressed in tumors compared with normal tissues in DE gene analysis, and patients with ‘high’ expression demonstrate poor survival, except for AC079612.1. C and D Boxplots comparing expression in tumor stage (C) and MSI categories (D). Expression levels of AL127134.2 increase with according to microsatellite. (**** p < 0.0001, *** p < 0.0005, ** p < 0.005, * p < 0.05)

## Slide 2
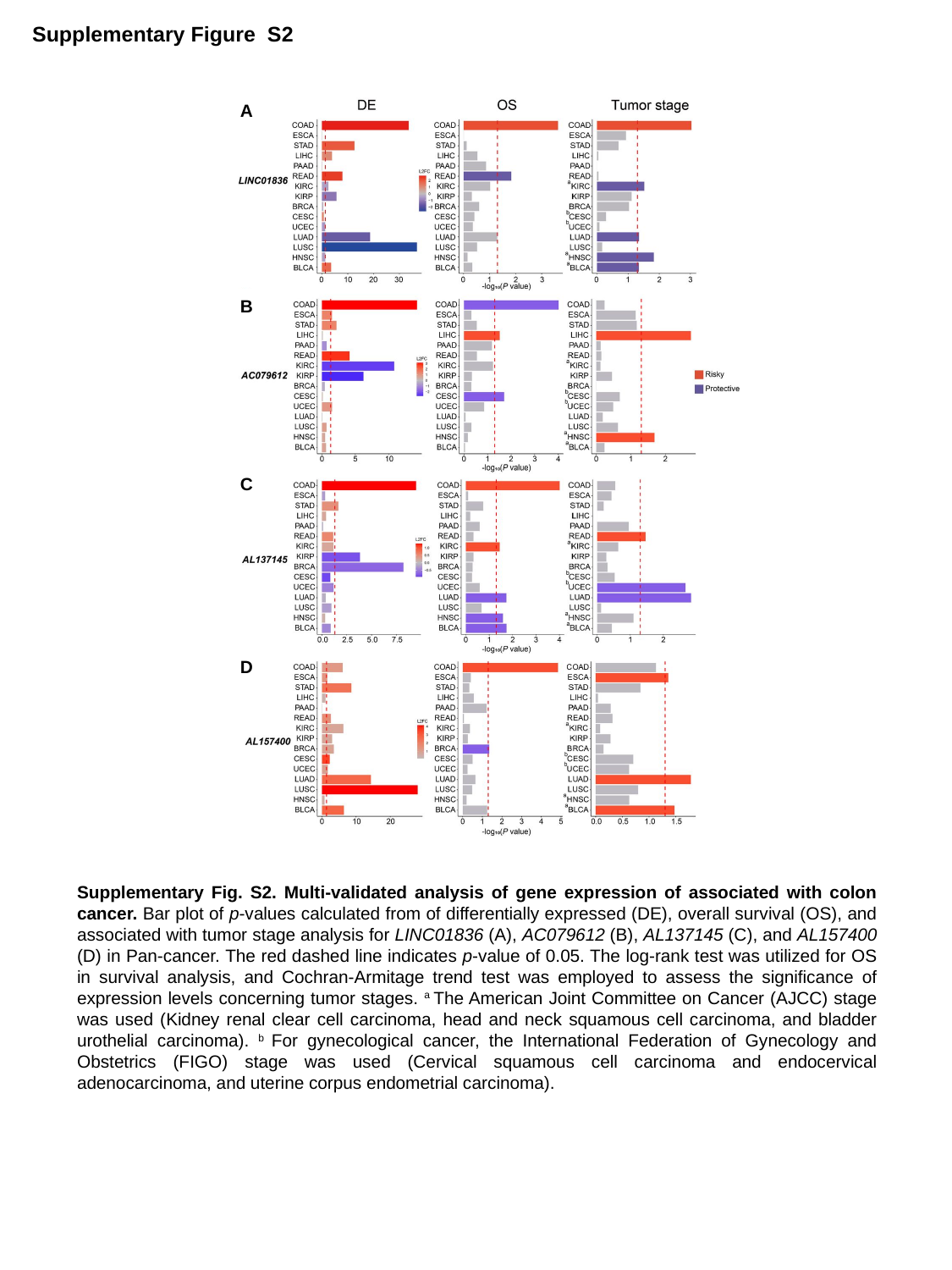

Supplementary Figure S2
A
B
C
D
Supplementary Fig. S2. Multi-validated analysis of gene expression of associated with colon cancer. Bar plot of p-values calculated from of differentially expressed (DE), overall survival (OS), and associated with tumor stage analysis for LINC01836 (A), AC079612 (B), AL137145 (C), and AL157400 (D) in Pan-cancer. The red dashed line indicates p-value of 0.05. The log-rank test was utilized for OS in survival analysis, and Cochran-Armitage trend test was employed to assess the significance of expression levels concerning tumor stages. a The American Joint Committee on Cancer (AJCC) stage was used (Kidney renal clear cell carcinoma, head and neck squamous cell carcinoma, and bladder urothelial carcinoma). b For gynecological cancer, the International Federation of Gynecology and Obstetrics (FIGO) stage was used (Cervical squamous cell carcinoma and endocervical adenocarcinoma, and uterine corpus endometrial carcinoma).

## Slide 3
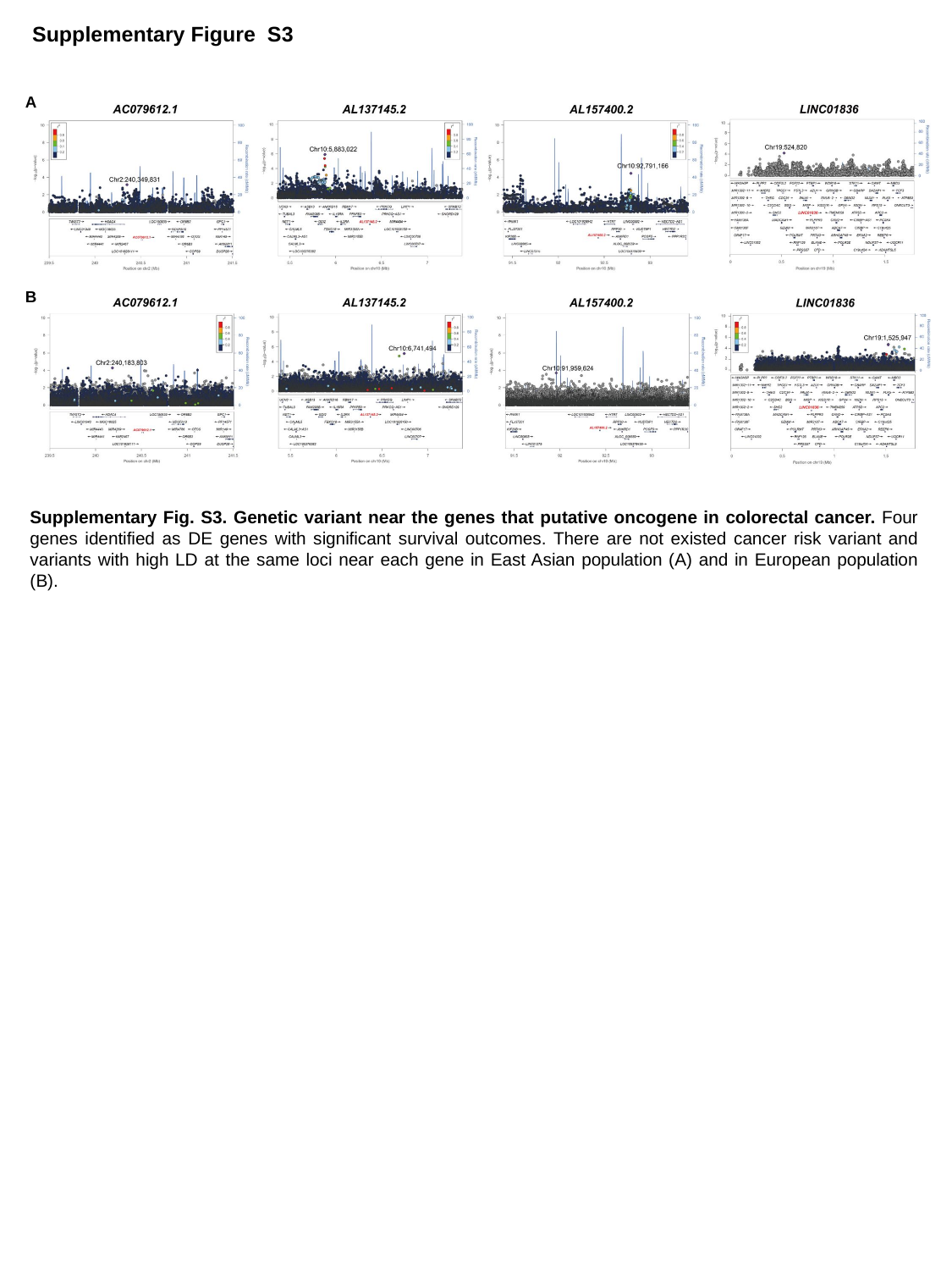

Supplementary Figure S3
A
B
Supplementary Fig. S3. Genetic variant near the genes that putative oncogene in colorectal cancer. Four genes identified as DE genes with significant survival outcomes. There are not existed cancer risk variant and variants with high LD at the same loci near each gene in East Asian population (A) and in European population (B).

## Slide 4
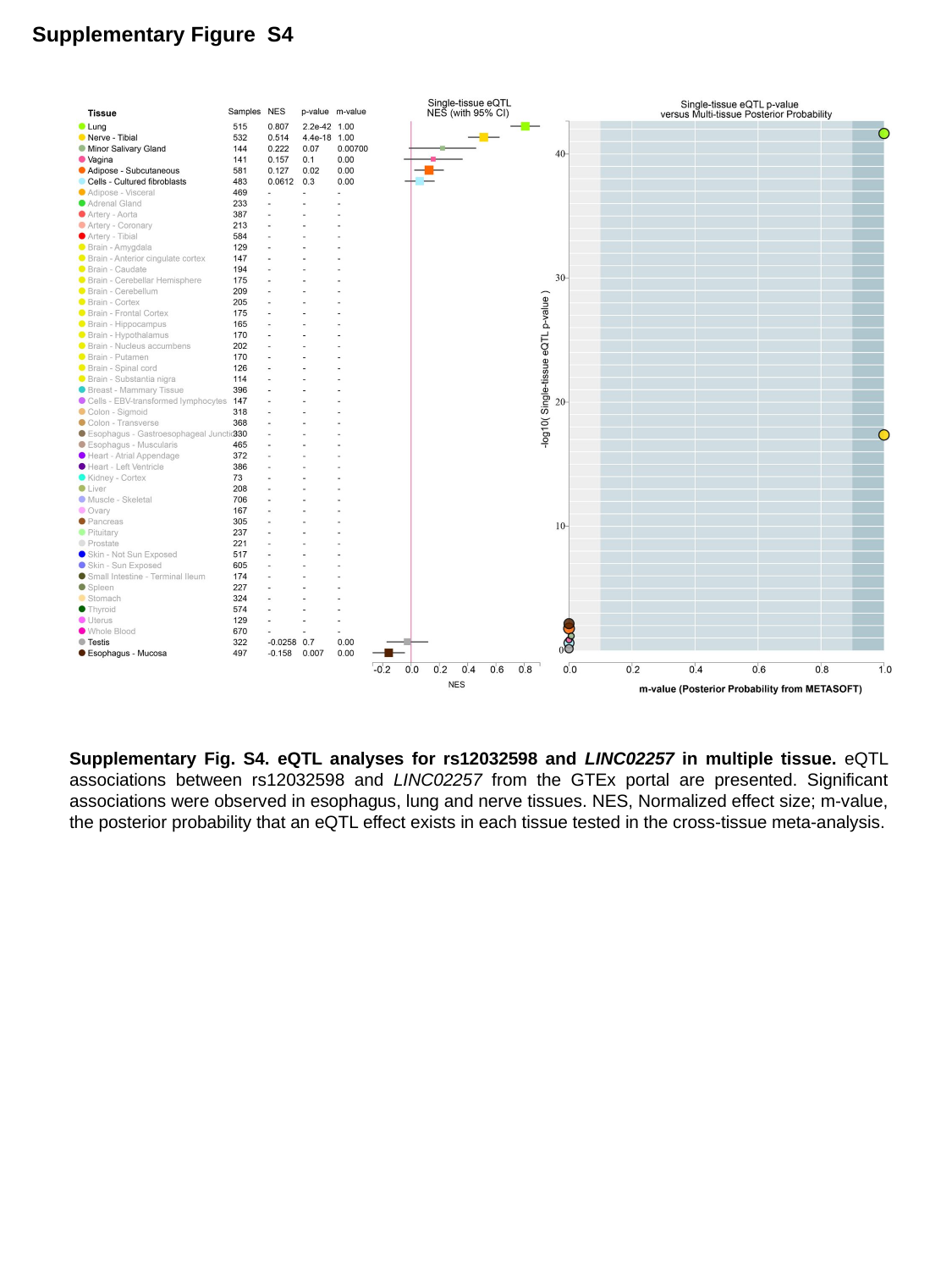

Supplementary Figure S4
Supplementary Fig. S4. eQTL analyses for rs12032598 and LINC02257 in multiple tissue. eQTL associations between rs12032598 and LINC02257 from the GTEx portal are presented. Significant associations were observed in esophagus, lung and nerve tissues. NES, Normalized effect size; m-value, the posterior probability that an eQTL effect exists in each tissue tested in the cross-tissue meta-analysis.

## Slide 5
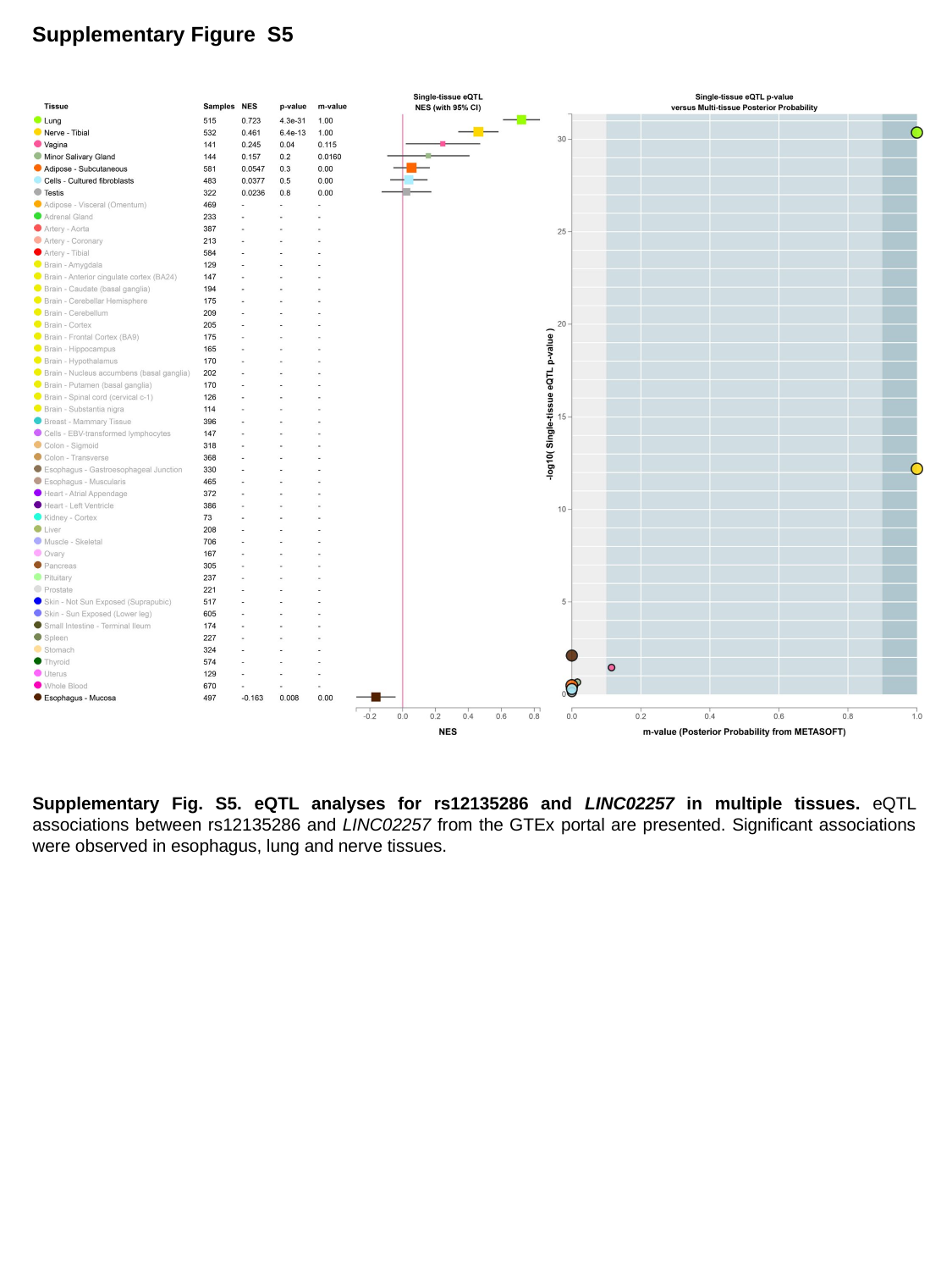

Supplementary Figure S5
Supplementary Fig. S5. eQTL analyses for rs12135286 and LINC02257 in multiple tissues. eQTL associations between rs12135286 and LINC02257 from the GTEx portal are presented. Significant associations were observed in esophagus, lung and nerve tissues.

## Slide 6
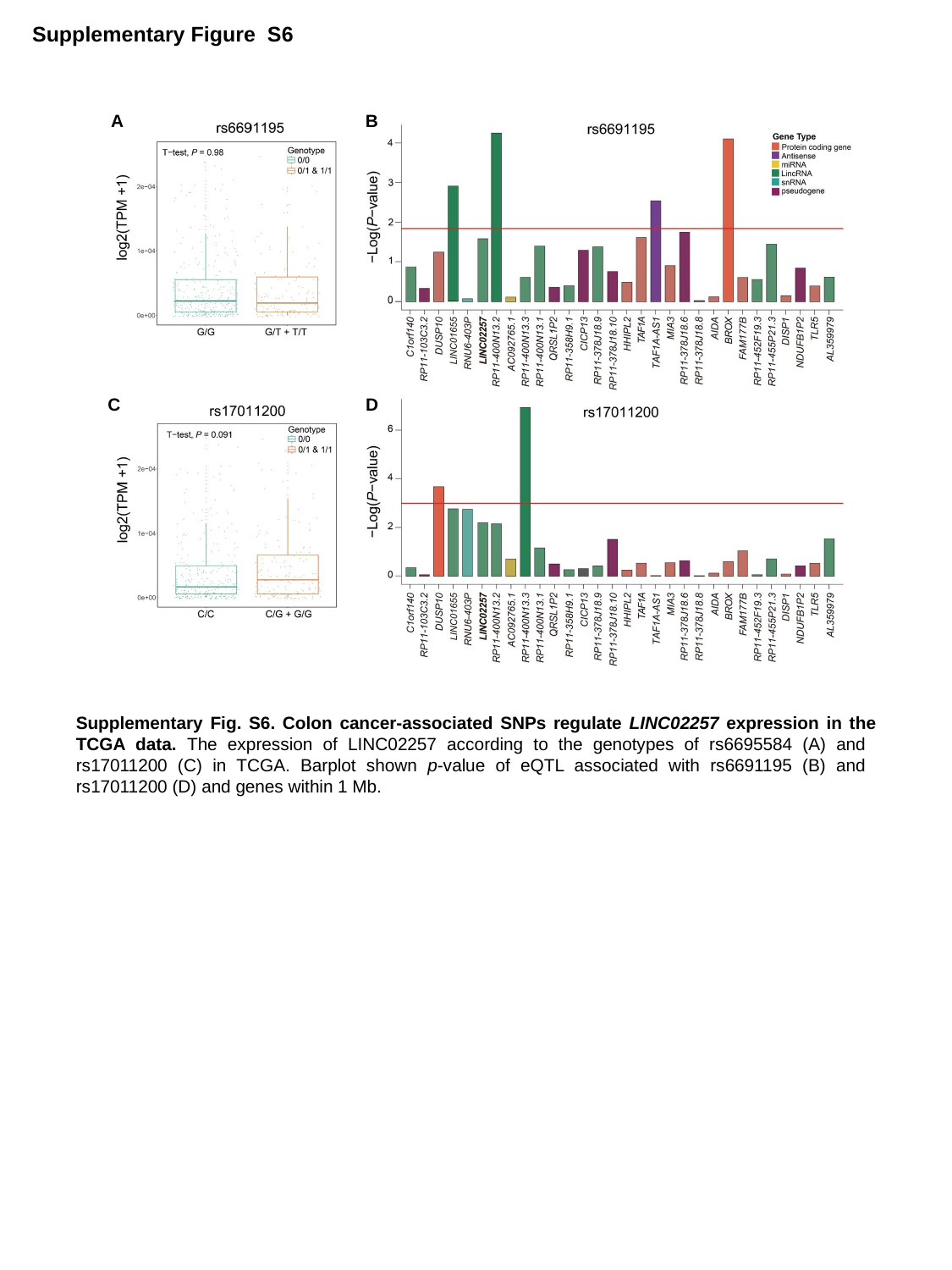

Supplementary Figure S6
A
B
C
D
Supplementary Fig. S6. Colon cancer-associated SNPs regulate LINC02257 expression in the TCGA data. The expression of LINC02257 according to the genotypes of rs6695584 (A) and rs17011200 (C) in TCGA. Barplot shown p-value of eQTL associated with rs6691195 (B) and rs17011200 (D) and genes within 1 Mb.

## Slide 7
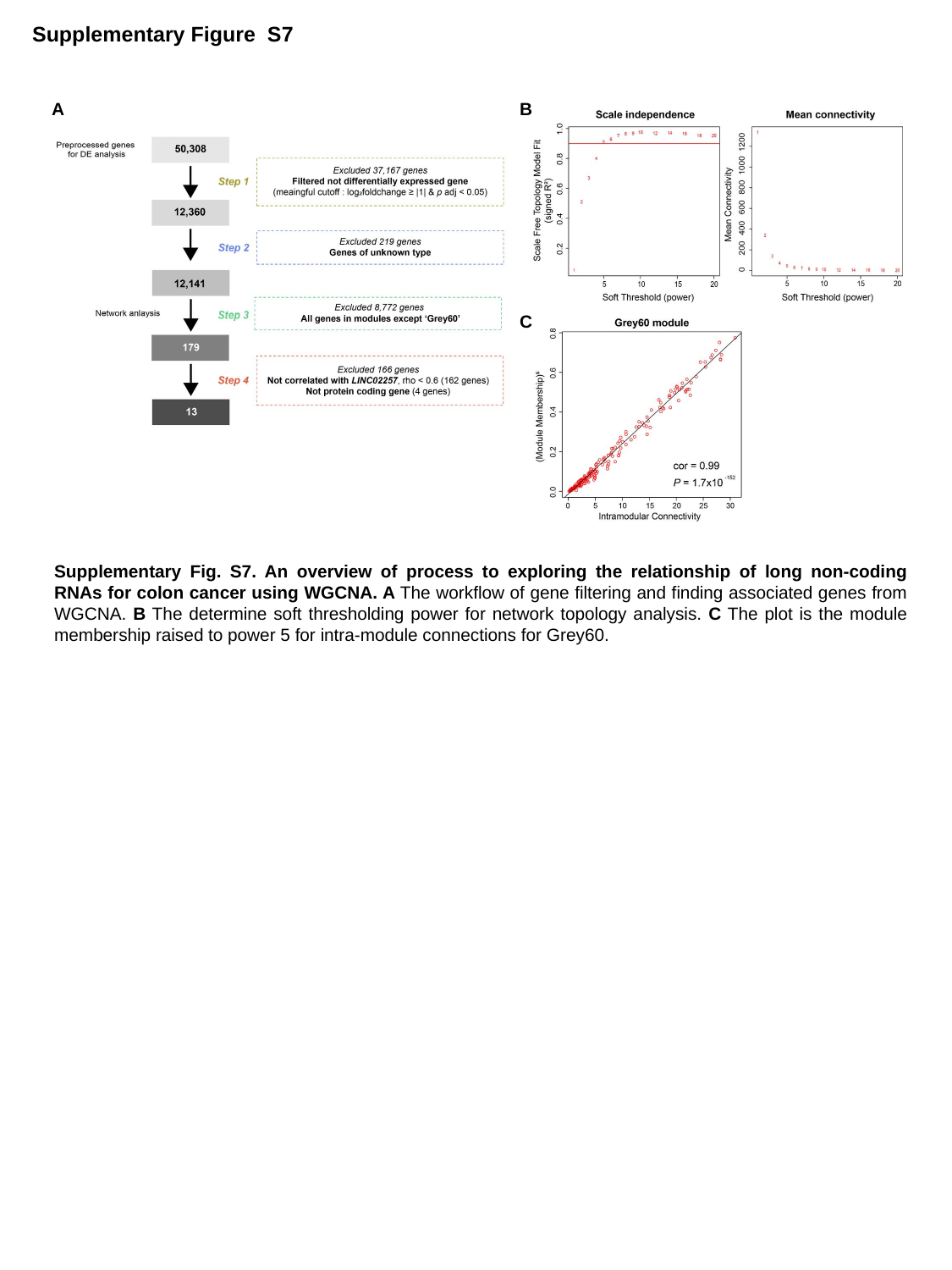

Supplementary Figure S7
A
B
C
Supplementary Fig. S7. An overview of process to exploring the relationship of long non-coding RNAs for colon cancer using WGCNA. A The workflow of gene filtering and finding associated genes from WGCNA. B The determine soft thresholding power for network topology analysis. C The plot is the module membership raised to power 5 for intra-module connections for Grey60.

## Slide 8
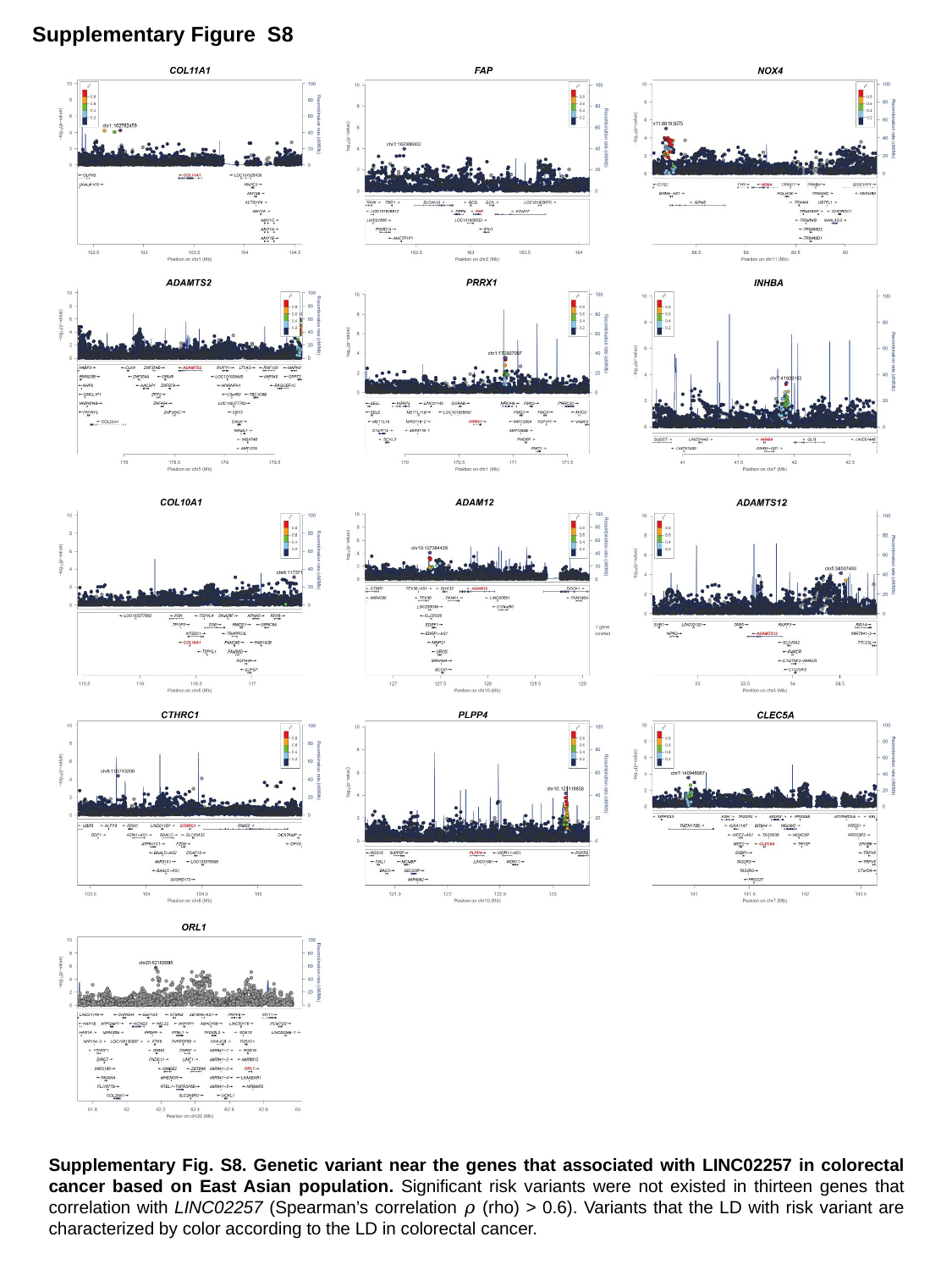

Supplementary Figure S8
Supplementary Fig. S8. Genetic variant near the genes that associated with LINC02257 in colorectal cancer based on East Asian population. Significant risk variants were not existed in thirteen genes that correlation with LINC02257 (Spearman’s correlation 𝜌 (rho) > 0.6). Variants that the LD with risk variant are characterized by color according to the LD in colorectal cancer.

## Slide 9
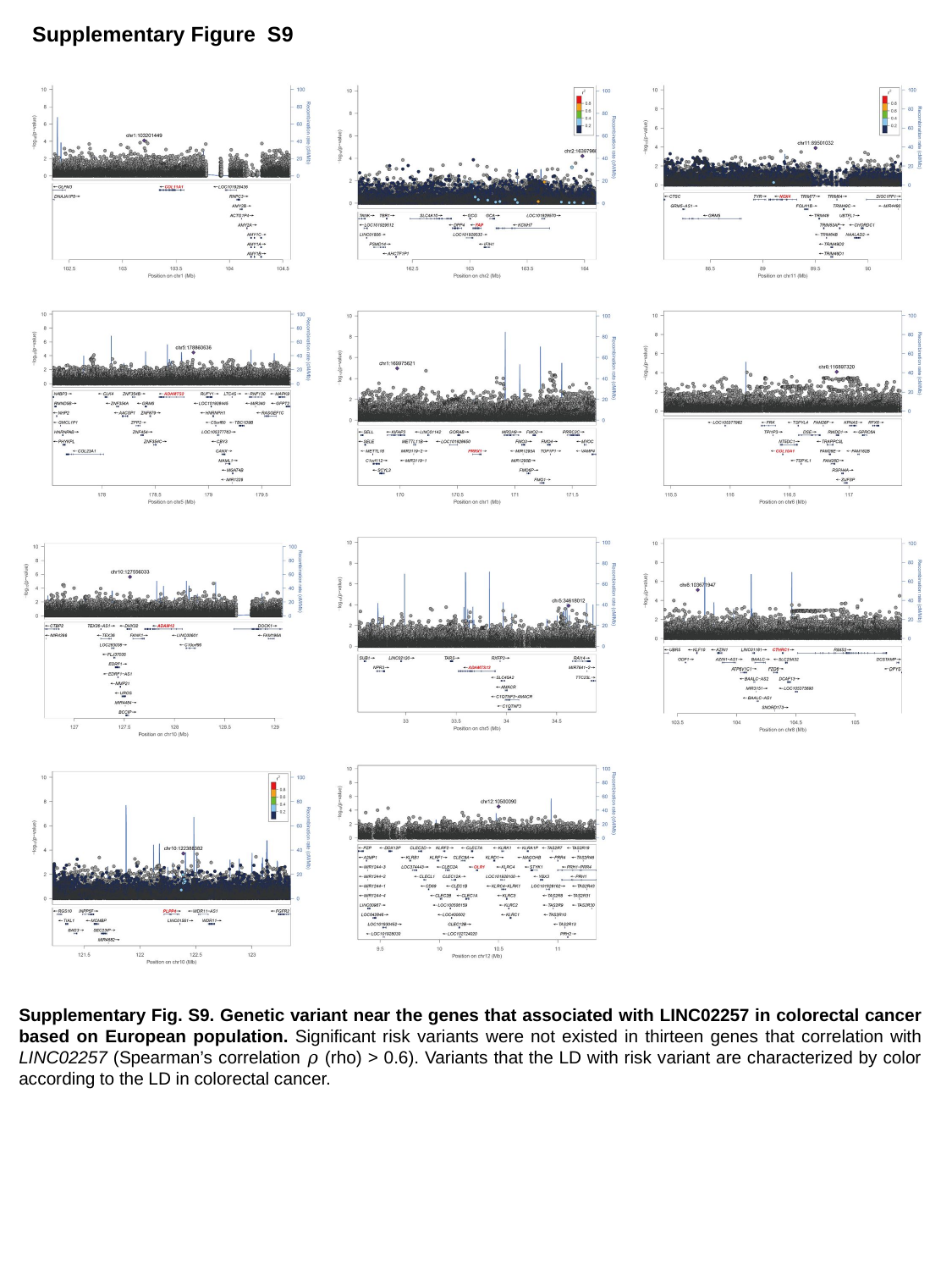

Supplementary Figure S9
Supplementary Fig. S9. Genetic variant near the genes that associated with LINC02257 in colorectal cancer based on European population. Significant risk variants were not existed in thirteen genes that correlation with LINC02257 (Spearman’s correlation 𝜌 (rho) > 0.6). Variants that the LD with risk variant are characterized by color according to the LD in colorectal cancer.

## Slide 10
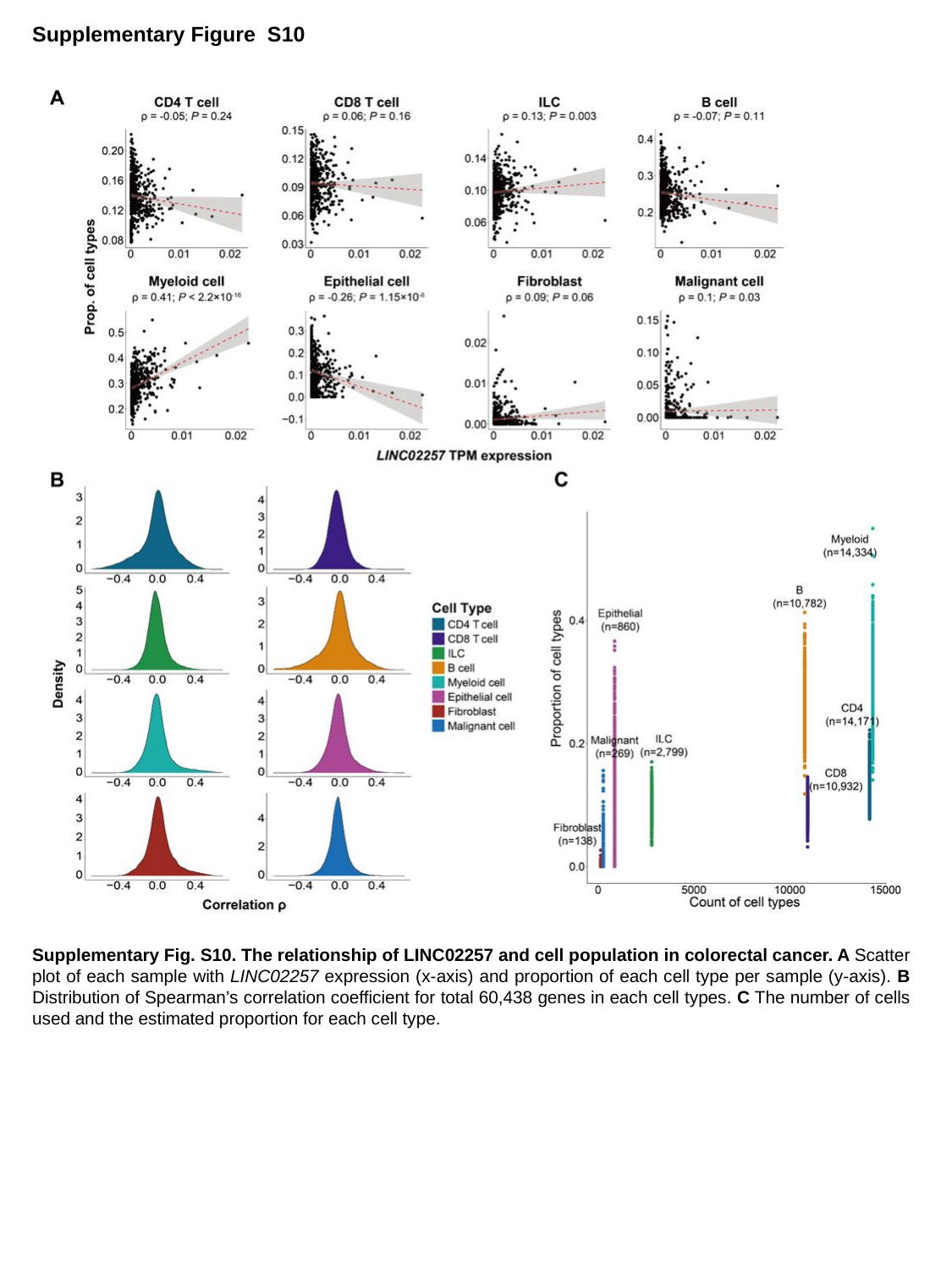

Supplementary Figure S10
Supplementary Fig. S10. The relationship of LINC02257 and cell population in colorectal cancer. A Scatter plot of each sample with LINC02257 expression (x-axis) and proportion of each cell type per sample (y-axis). B Distribution of Spearman’s correlation coefficient for total 60,438 genes in each cell types. C The number of cells used and the estimated proportion for each cell type.

## Slide 11
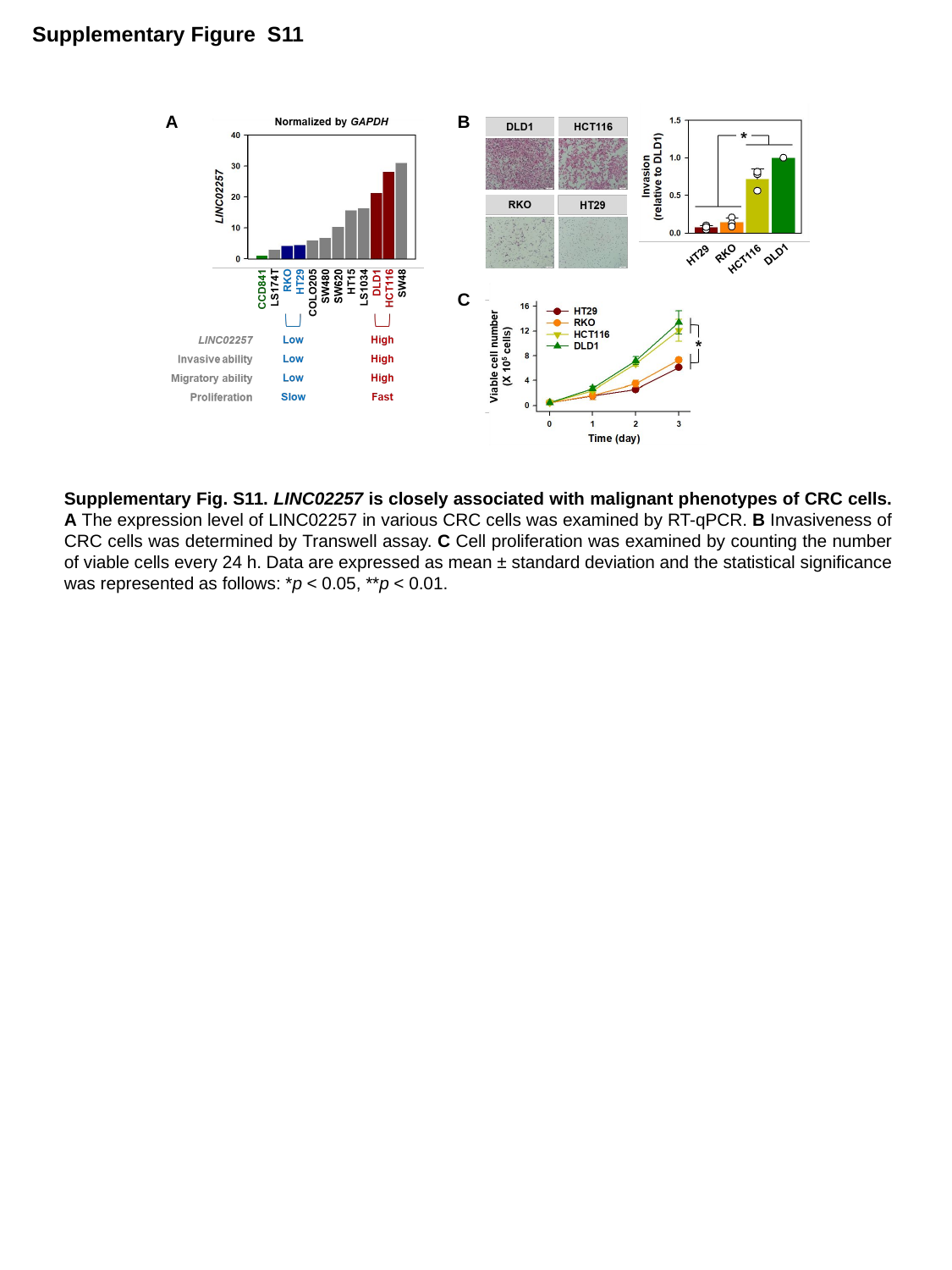

Supplementary Figure S11
A
B
C
Supplementary Fig. S11. LINC02257 is closely associated with malignant phenotypes of CRC cells. A The expression level of LINC02257 in various CRC cells was examined by RT-qPCR. B Invasiveness of CRC cells was determined by Transwell assay. C Cell proliferation was examined by counting the number of viable cells every 24 h. Data are expressed as mean ± standard deviation and the statistical significance was represented as follows: *p < 0.05, **p < 0.01.

## Slide 12
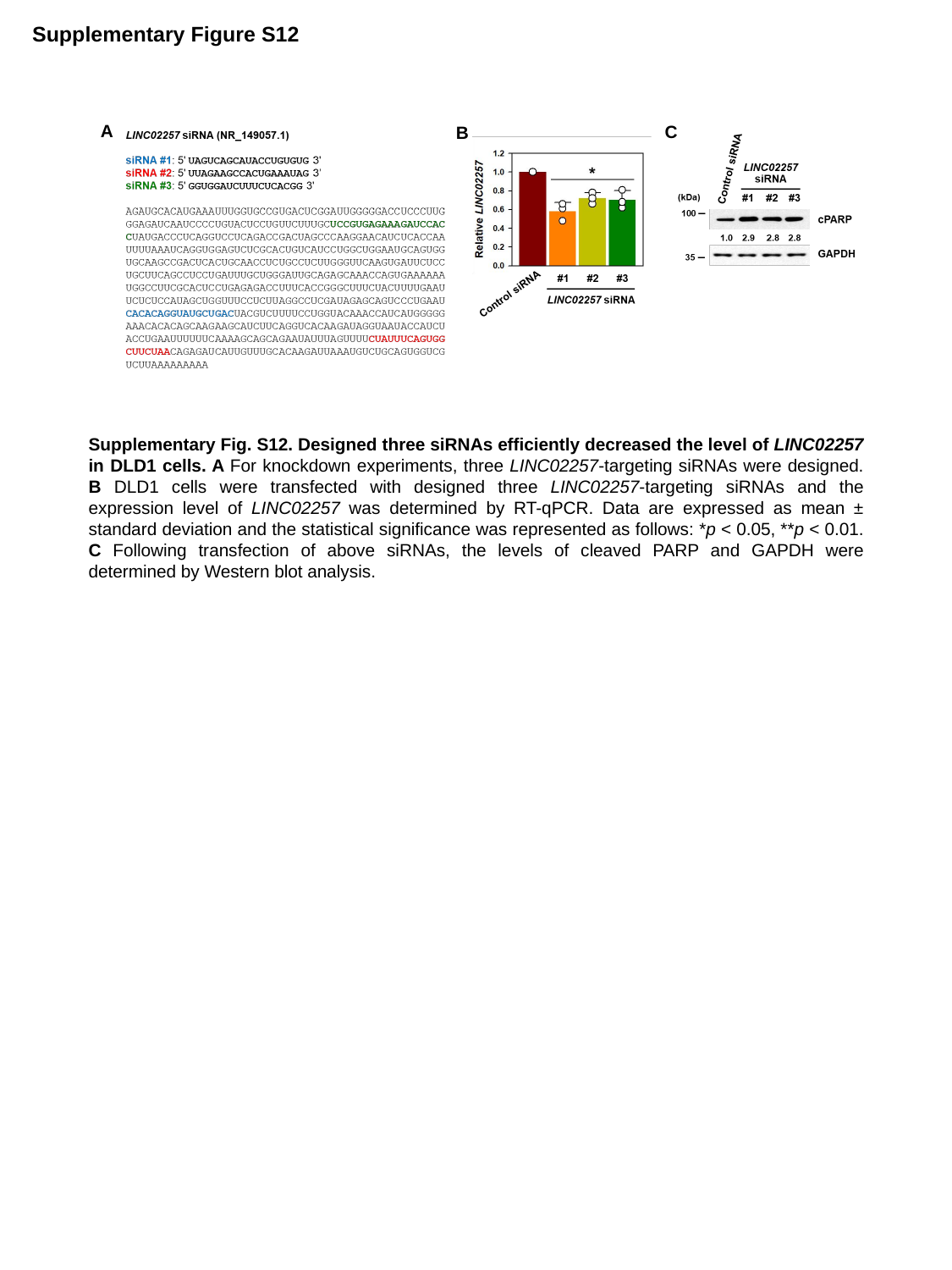

Supplementary Figure S12
A
C
B
Supplementary Fig. S12. Designed three siRNAs efficiently decreased the level of LINC02257 in DLD1 cells. A For knockdown experiments, three LINC02257-targeting siRNAs were designed. B DLD1 cells were transfected with designed three LINC02257-targeting siRNAs and the expression level of LINC02257 was determined by RT-qPCR. Data are expressed as mean ± standard deviation and the statistical significance was represented as follows: *p < 0.05, **p < 0.01. C Following transfection of above siRNAs, the levels of cleaved PARP and GAPDH were determined by Western blot analysis.

## Slide 13
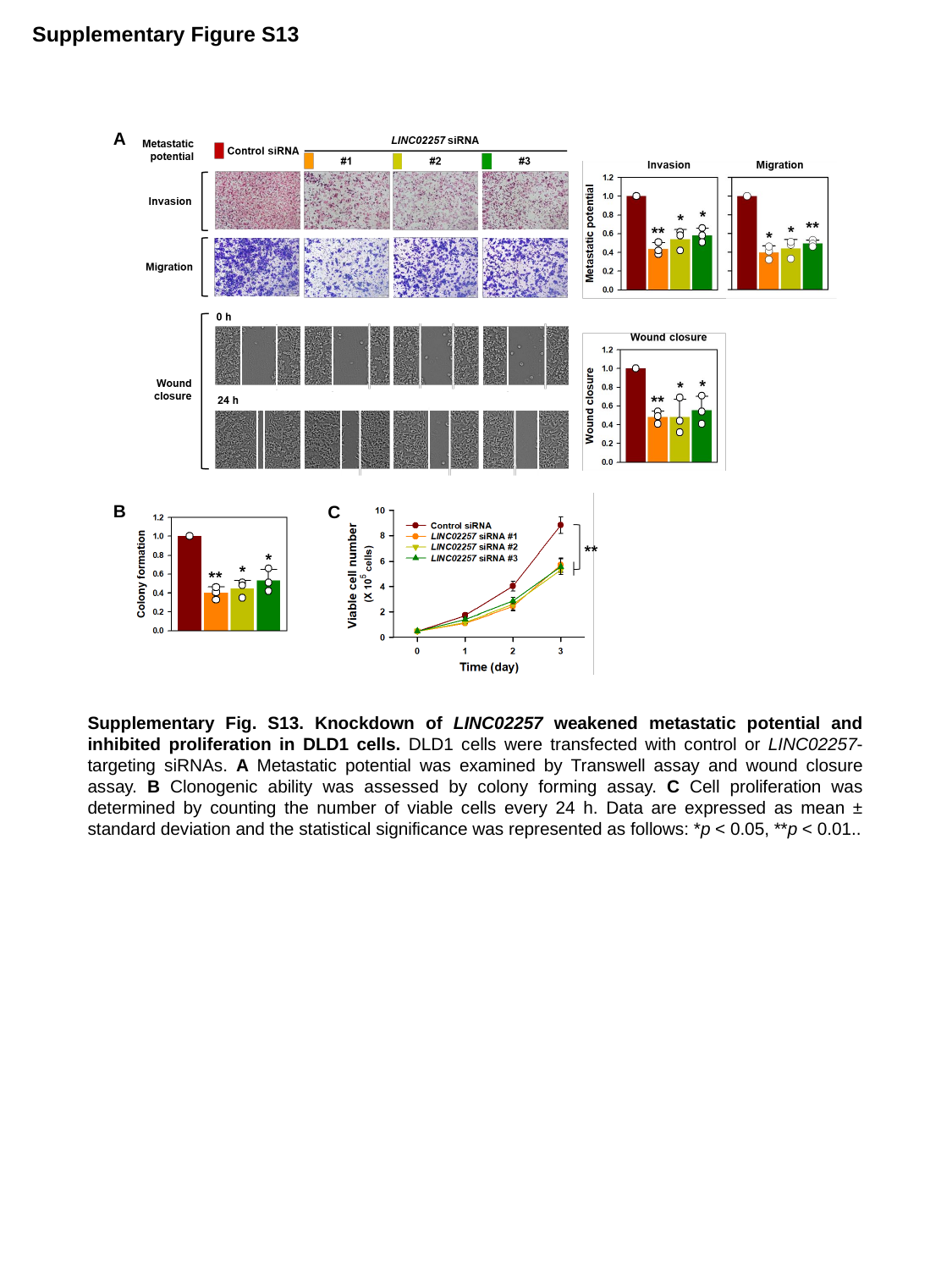

Supplementary Figure S13
A
B
C
Supplementary Fig. S13. Knockdown of LINC02257 weakened metastatic potential and inhibited proliferation in DLD1 cells. DLD1 cells were transfected with control or LINC02257-targeting siRNAs. A Metastatic potential was examined by Transwell assay and wound closure assay. B Clonogenic ability was assessed by colony forming assay. C Cell proliferation was determined by counting the number of viable cells every 24 h. Data are expressed as mean ± standard deviation and the statistical significance was represented as follows: *p < 0.05, **p < 0.01..

## Slide 14
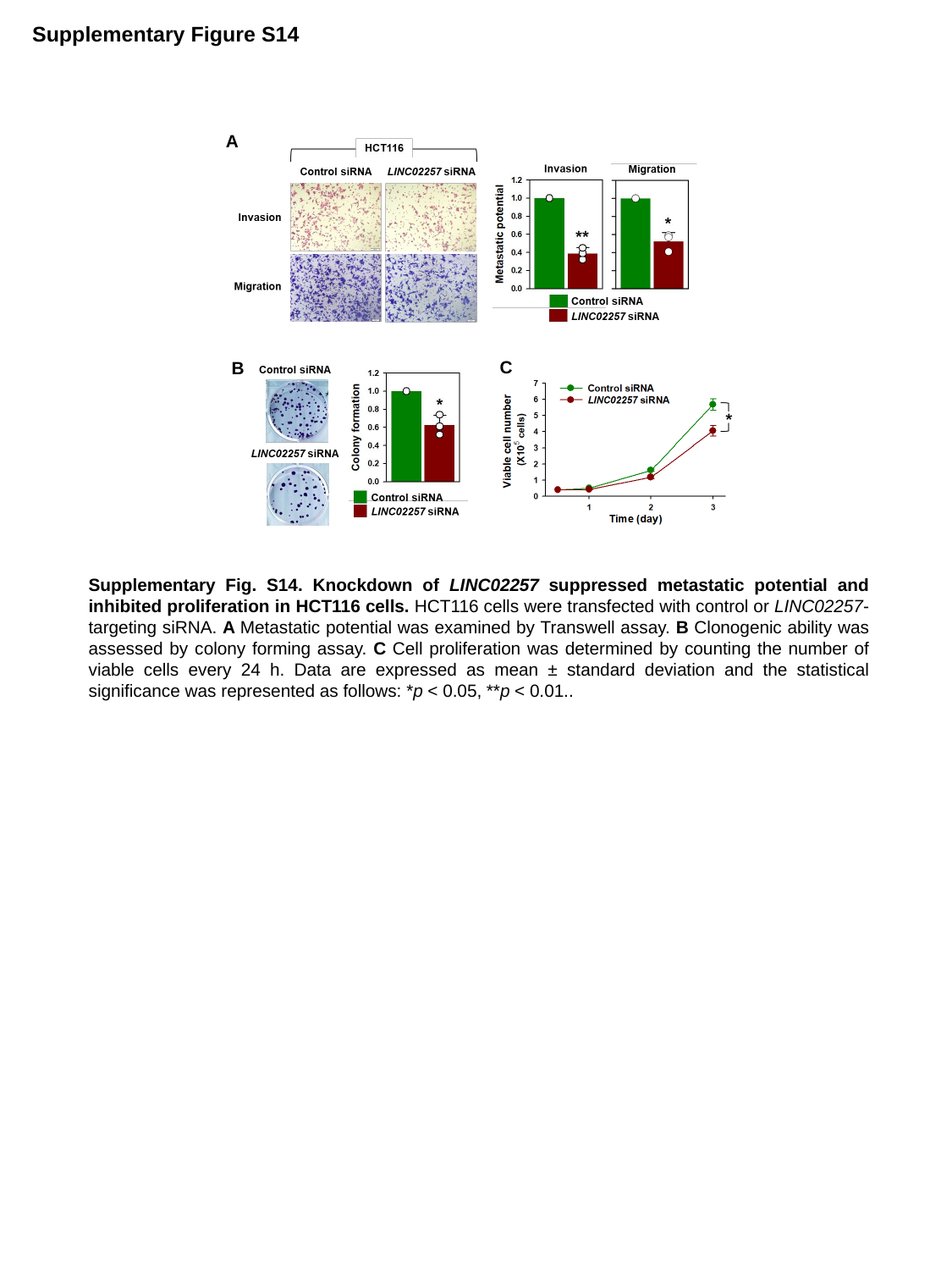

Supplementary Figure S14
A
C
B
Supplementary Fig. S14. Knockdown of LINC02257 suppressed metastatic potential and inhibited proliferation in HCT116 cells. HCT116 cells were transfected with control or LINC02257-targeting siRNA. A Metastatic potential was examined by Transwell assay. B Clonogenic ability was assessed by colony forming assay. C Cell proliferation was determined by counting the number of viable cells every 24 h. Data are expressed as mean ± standard deviation and the statistical significance was represented as follows: *p < 0.05, **p < 0.01..

## Slide 15
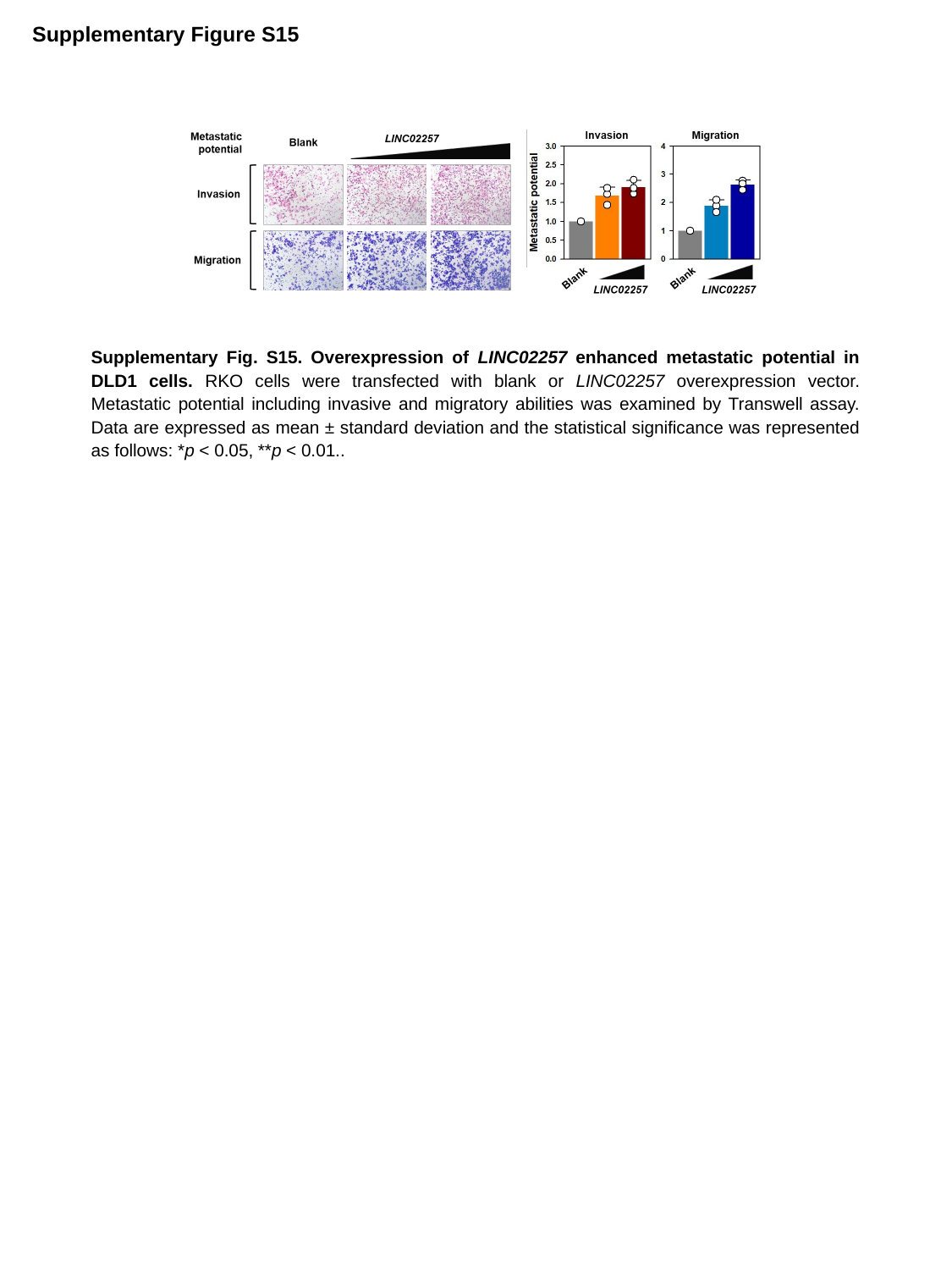

Supplementary Figure S15
Supplementary Fig. S15. Overexpression of LINC02257 enhanced metastatic potential in DLD1 cells. RKO cells were transfected with blank or LINC02257 overexpression vector. Metastatic potential including invasive and migratory abilities was examined by Transwell assay. Data are expressed as mean ± standard deviation and the statistical significance was represented as follows: *p < 0.05, **p < 0.01..

## Slide 16
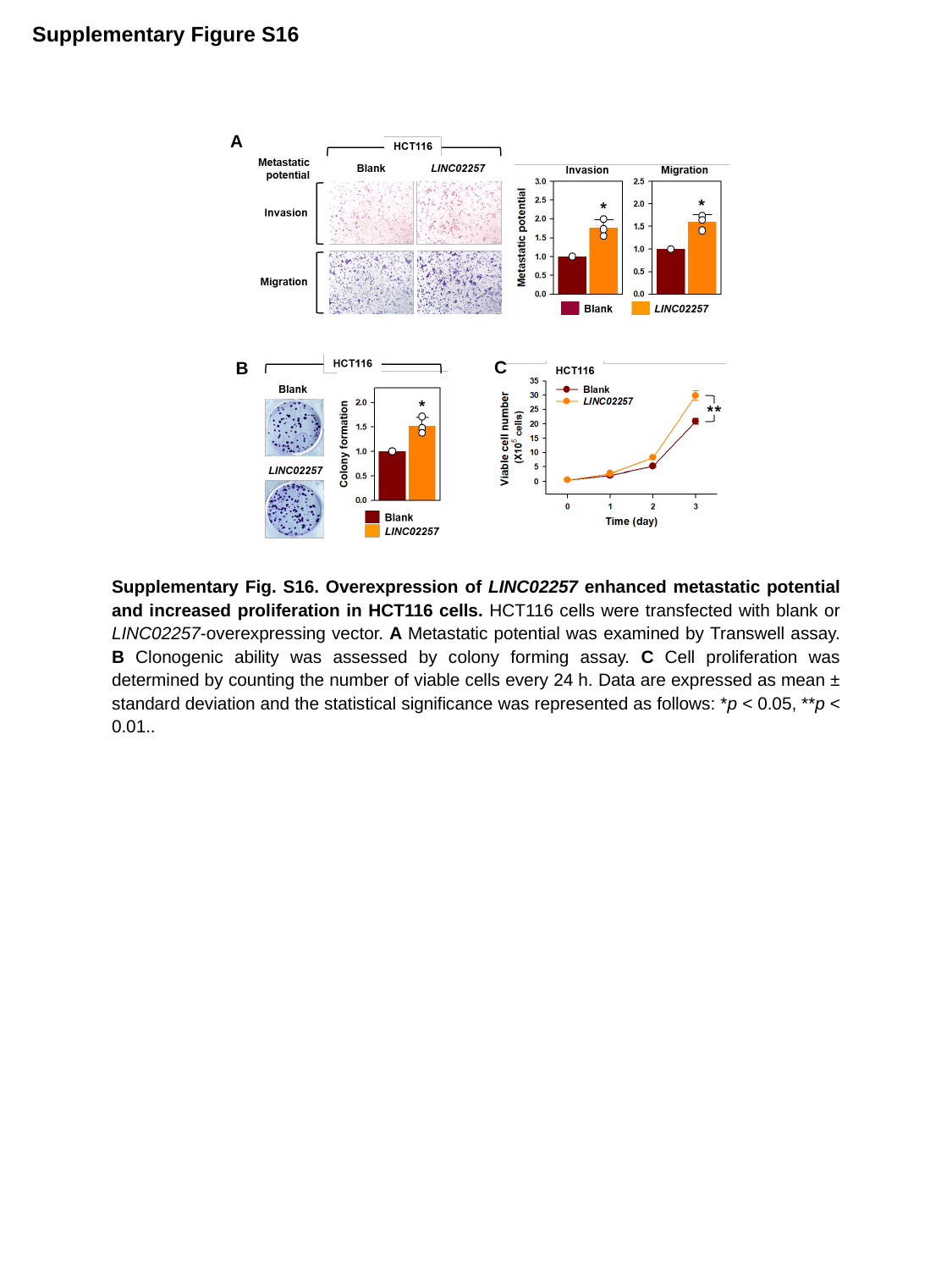

Supplementary Figure S16
A
C
B
Supplementary Fig. S16. Overexpression of LINC02257 enhanced metastatic potential and increased proliferation in HCT116 cells. HCT116 cells were transfected with blank or LINC02257-overexpressing vector. A Metastatic potential was examined by Transwell assay. B Clonogenic ability was assessed by colony forming assay. C Cell proliferation was determined by counting the number of viable cells every 24 h. Data are expressed as mean ± standard deviation and the statistical significance was represented as follows: *p < 0.05, **p < 0.01..

## Slide 17
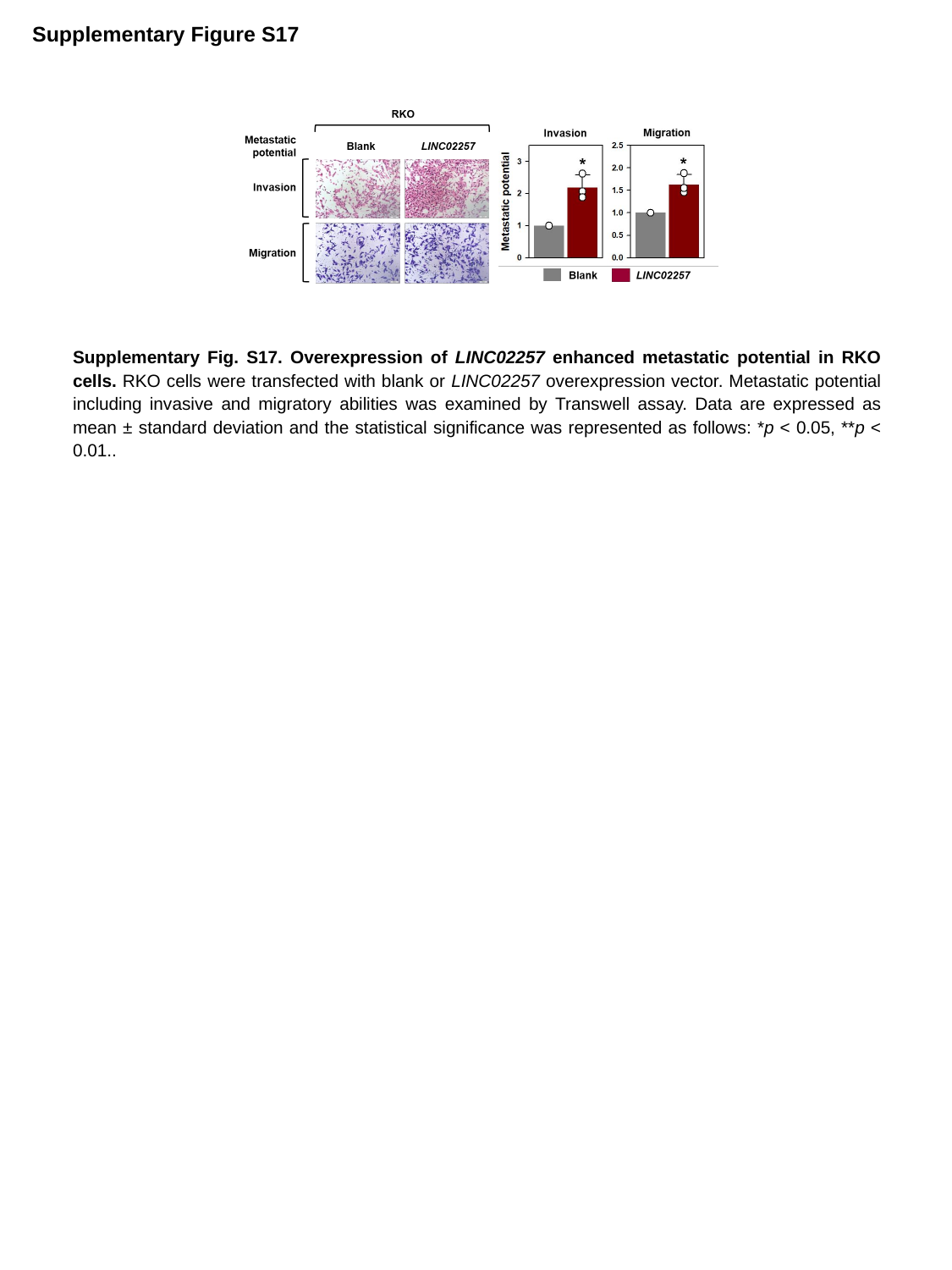

Supplementary Figure S17
Supplementary Fig. S17. Overexpression of LINC02257 enhanced metastatic potential in RKO cells. RKO cells were transfected with blank or LINC02257 overexpression vector. Metastatic potential including invasive and migratory abilities was examined by Transwell assay. Data are expressed as mean ± standard deviation and the statistical significance was represented as follows: *p < 0.05, **p < 0.01..

## Slide 18
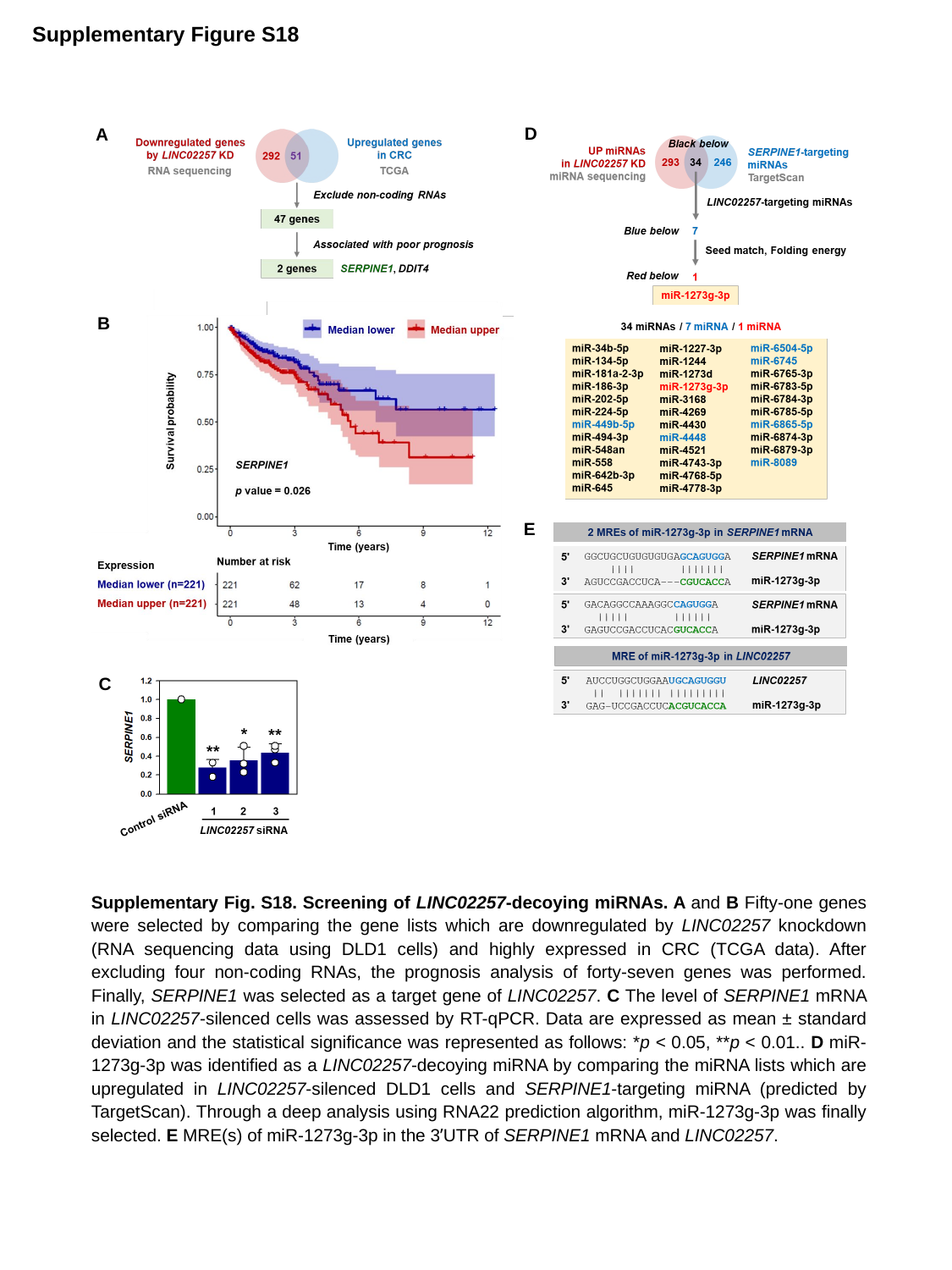

Supplementary Figure S18
D
A
B
E
C
Supplementary Fig. S18. Screening of LINC02257-decoying miRNAs. A and B Fifty-one genes were selected by comparing the gene lists which are downregulated by LINC02257 knockdown (RNA sequencing data using DLD1 cells) and highly expressed in CRC (TCGA data). After excluding four non-coding RNAs, the prognosis analysis of forty-seven genes was performed. Finally, SERPINE1 was selected as a target gene of LINC02257. C The level of SERPINE1 mRNA in LINC02257-silenced cells was assessed by RT-qPCR. Data are expressed as mean ± standard deviation and the statistical significance was represented as follows: *p < 0.05, **p < 0.01.. D miR-1273g-3p was identified as a LINC02257-decoying miRNA by comparing the miRNA lists which are upregulated in LINC02257-silenced DLD1 cells and SERPINE1-targeting miRNA (predicted by TargetScan). Through a deep analysis using RNA22 prediction algorithm, miR-1273g-3p was finally selected. E MRE(s) of miR-1273g-3p in the 3’UTR of SERPINE1 mRNA and LINC02257.

## Slide 19
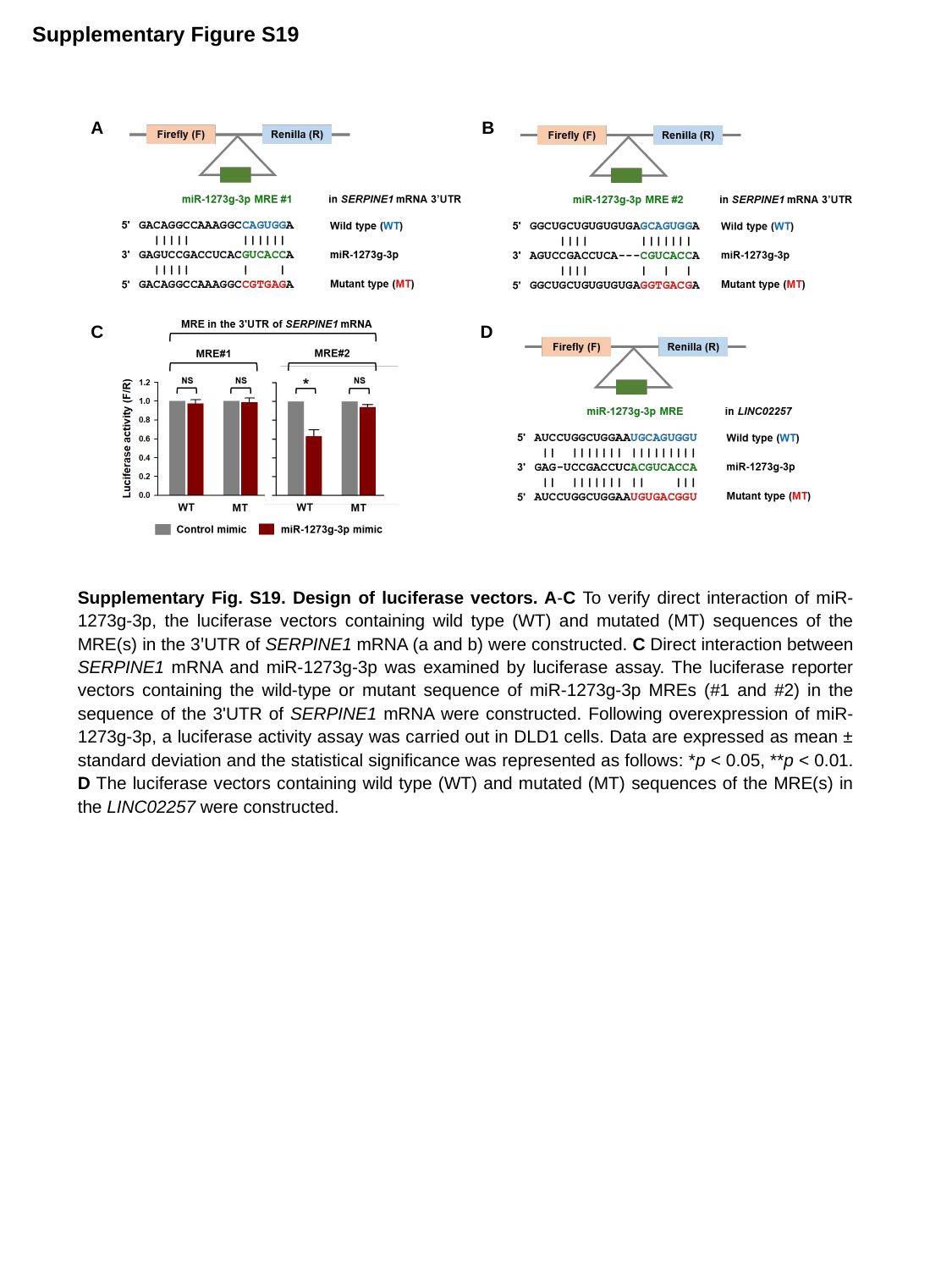

Supplementary Figure S19
A
B
C
D
Supplementary Fig. S19. Design of luciferase vectors. A-C To verify direct interaction of miR-1273g-3p, the luciferase vectors containing wild type (WT) and mutated (MT) sequences of the MRE(s) in the 3'UTR of SERPINE1 mRNA (a and b) were constructed. C Direct interaction between SERPINE1 mRNA and miR-1273g-3p was examined by luciferase assay. The luciferase reporter vectors containing the wild-type or mutant sequence of miR-1273g-3p MREs (#1 and #2) in the sequence of the 3'UTR of SERPINE1 mRNA were constructed. Following overexpression of miR-1273g-3p, a luciferase activity assay was carried out in DLD1 cells. Data are expressed as mean ± standard deviation and the statistical significance was represented as follows: *p < 0.05, **p < 0.01. D The luciferase vectors containing wild type (WT) and mutated (MT) sequences of the MRE(s) in the LINC02257 were constructed.

## Slide 20
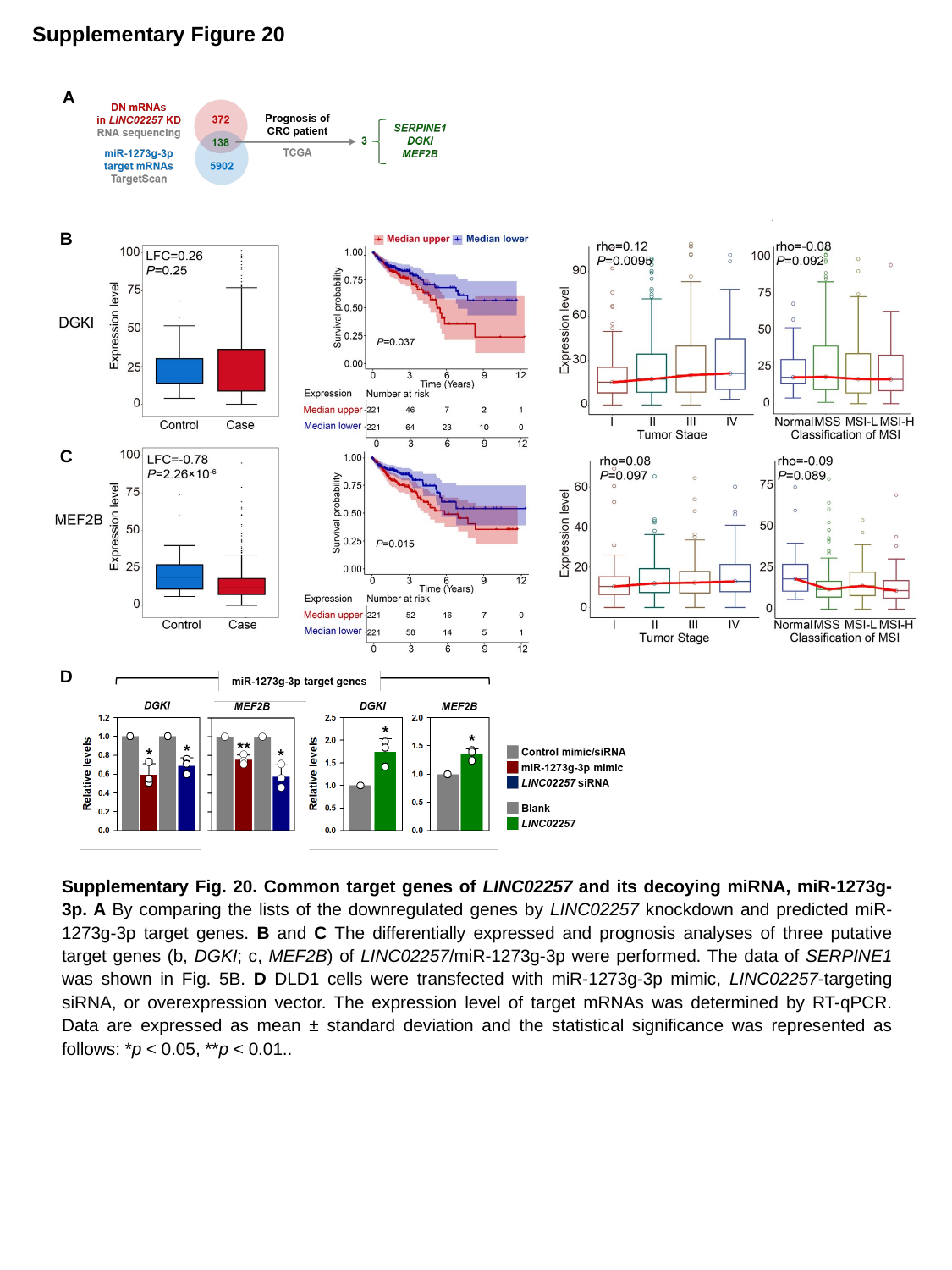

Supplementary Figure 20
A
B
C
D
Supplementary Fig. 20. Common target genes of LINC02257 and its decoying miRNA, miR-1273g-3p. A By comparing the lists of the downregulated genes by LINC02257 knockdown and predicted miR-1273g-3p target genes. B and C The differentially expressed and prognosis analyses of three putative target genes (b, DGKI; c, MEF2B) of LINC02257/miR-1273g-3p were performed. The data of SERPINE1 was shown in Fig. 5B. D DLD1 cells were transfected with miR-1273g-3p mimic, LINC02257-targeting siRNA, or overexpression vector. The expression level of target mRNAs was determined by RT-qPCR. Data are expressed as mean ± standard deviation and the statistical significance was represented as follows: *p < 0.05, **p < 0.01..

## Slide 21
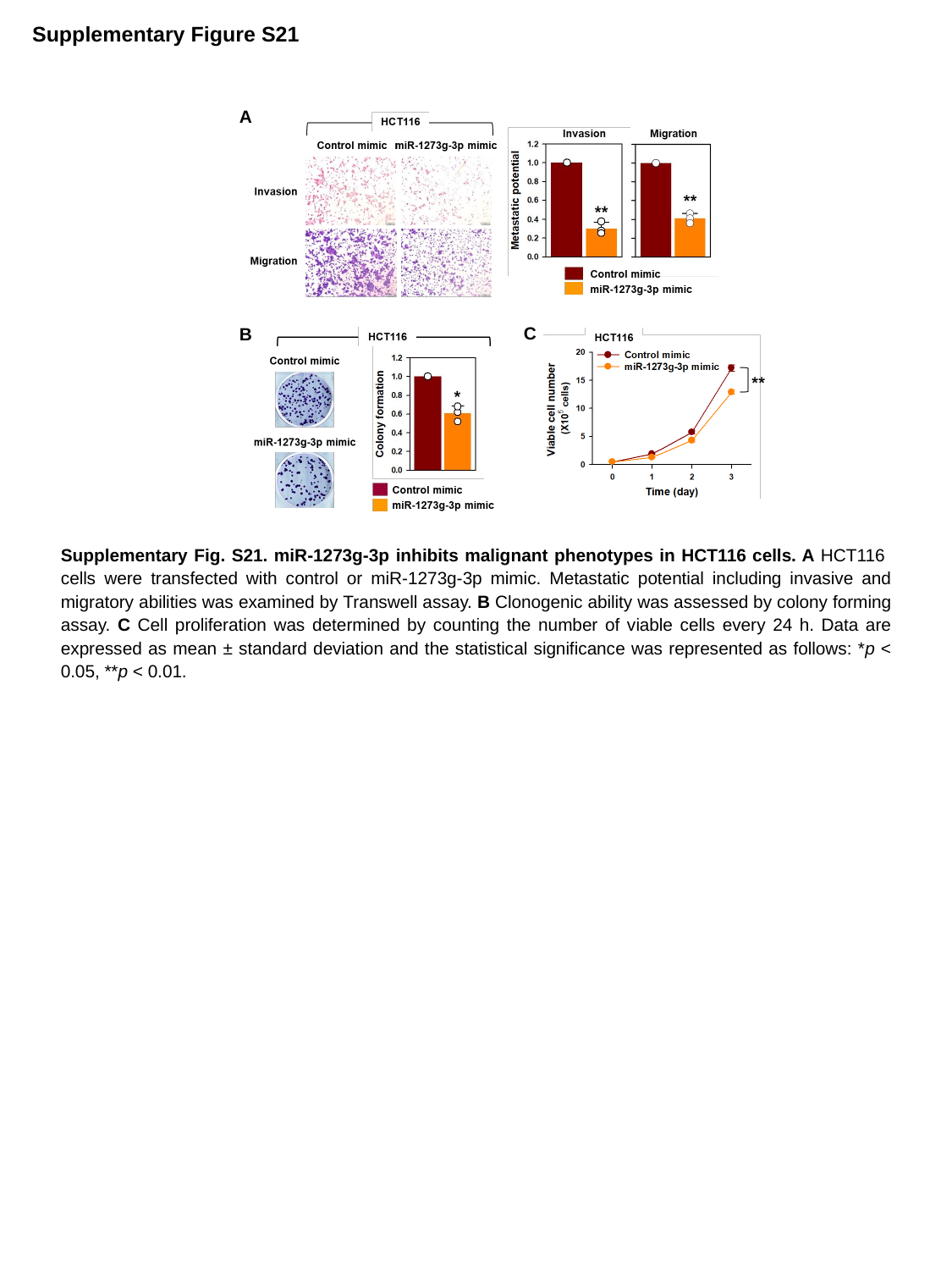

Supplementary Figure S21
A
C
B
Supplementary Fig. S21. miR-1273g-3p inhibits malignant phenotypes in HCT116 cells. A HCT116 cells were transfected with control or miR-1273g-3p mimic. Metastatic potential including invasive and migratory abilities was examined by Transwell assay. B Clonogenic ability was assessed by colony forming assay. C Cell proliferation was determined by counting the number of viable cells every 24 h. Data are expressed as mean ± standard deviation and the statistical significance was represented as follows: *p < 0.05, **p < 0.01.

## Slide 22
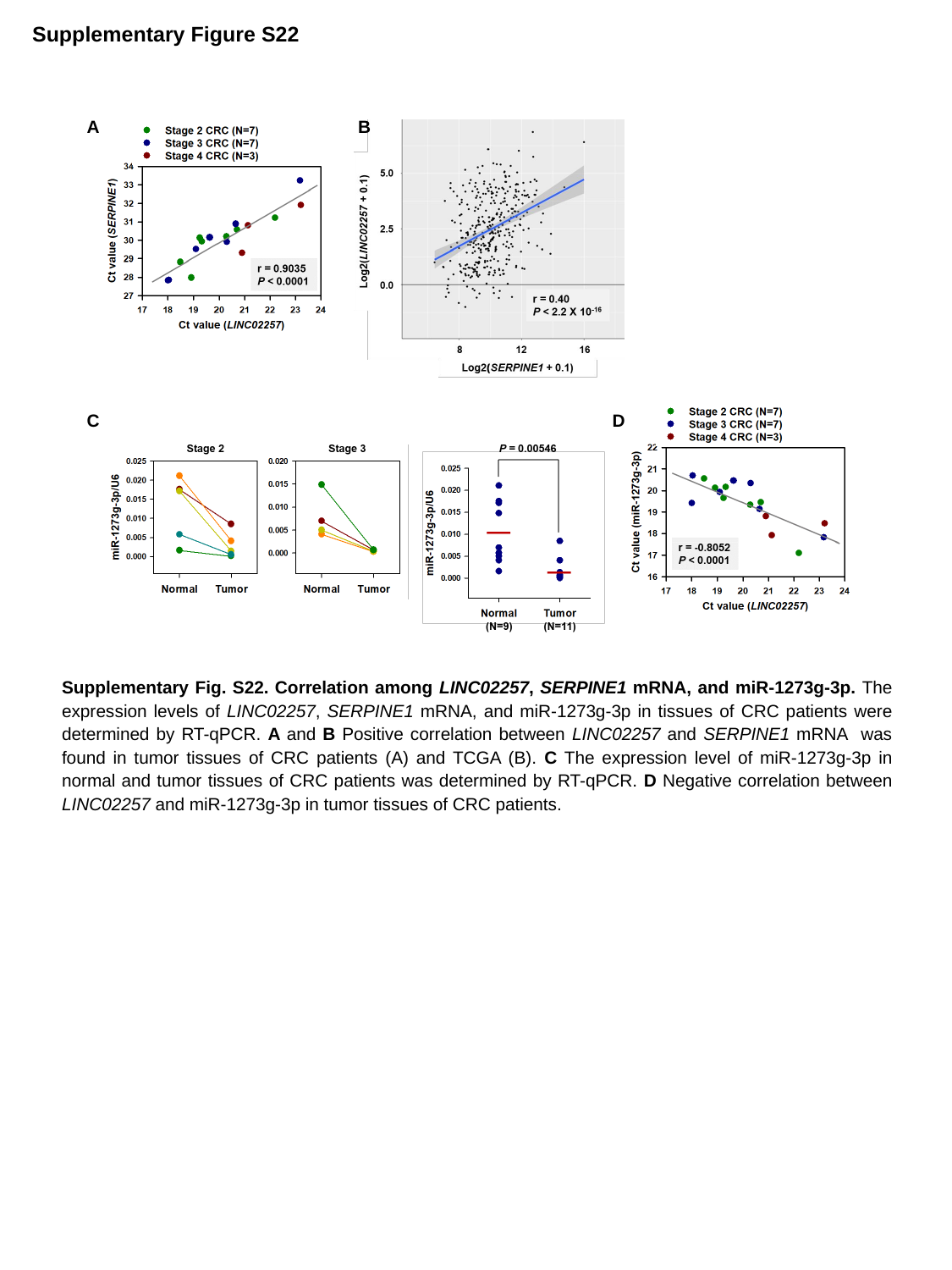

Supplementary Figure S22
B
A
C
D
Supplementary Fig. S22. Correlation among LINC02257, SERPINE1 mRNA, and miR-1273g-3p. The expression levels of LINC02257, SERPINE1 mRNA, and miR-1273g-3p in tissues of CRC patients were determined by RT-qPCR. A and B Positive correlation between LINC02257 and SERPINE1 mRNA was found in tumor tissues of CRC patients (A) and TCGA (B). C The expression level of miR-1273g-3p in normal and tumor tissues of CRC patients was determined by RT-qPCR. D Negative correlation between LINC02257 and miR-1273g-3p in tumor tissues of CRC patients.

## Slide 23
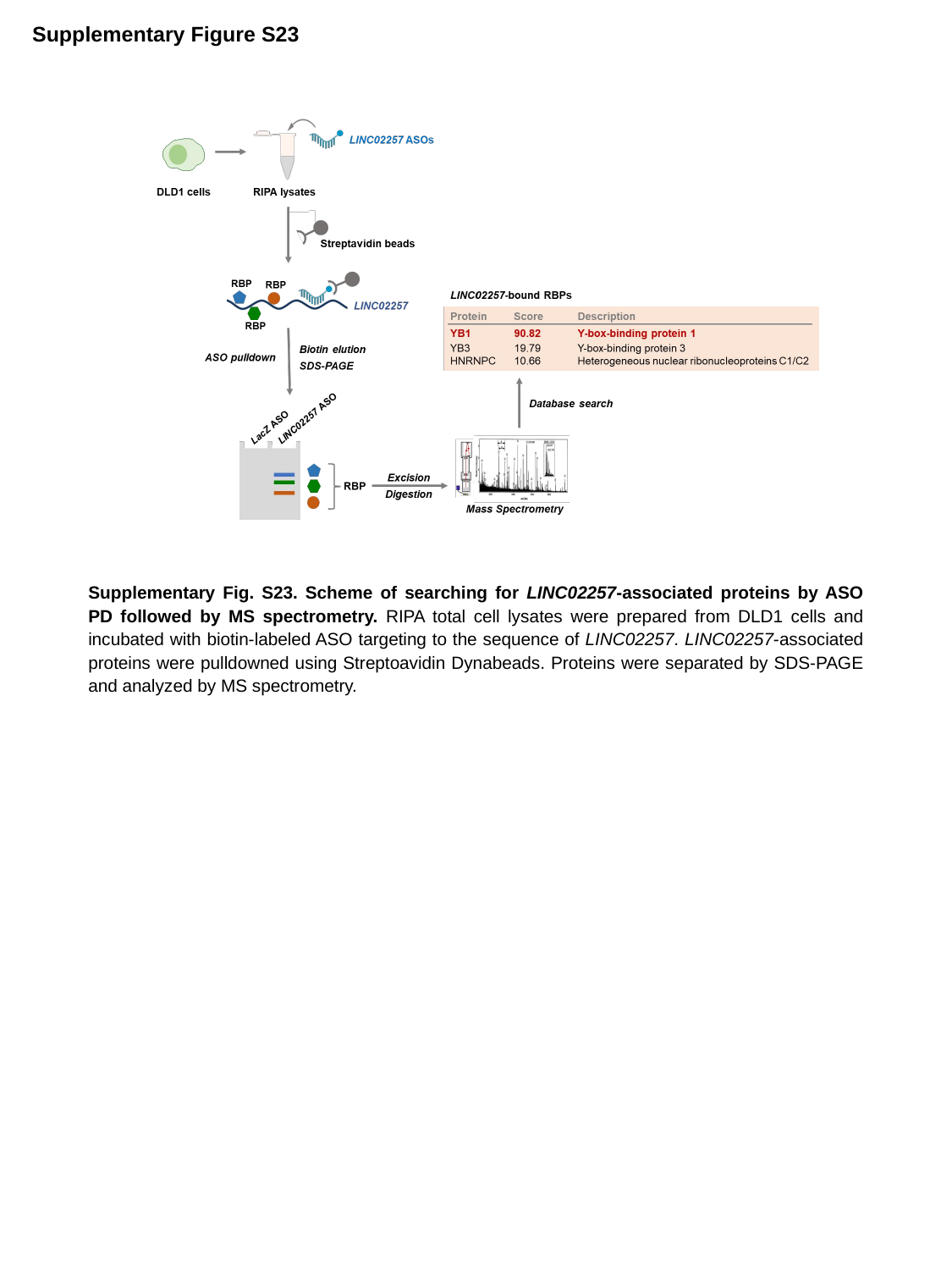

Supplementary Figure S23
Supplementary Fig. S23. Scheme of searching for LINC02257-associated proteins by ASO PD followed by MS spectrometry. RIPA total cell lysates were prepared from DLD1 cells and incubated with biotin-labeled ASO targeting to the sequence of LINC02257. LINC02257-associated proteins were pulldowned using Streptoavidin Dynabeads. Proteins were separated by SDS-PAGE and analyzed by MS spectrometry.

## Slide 24
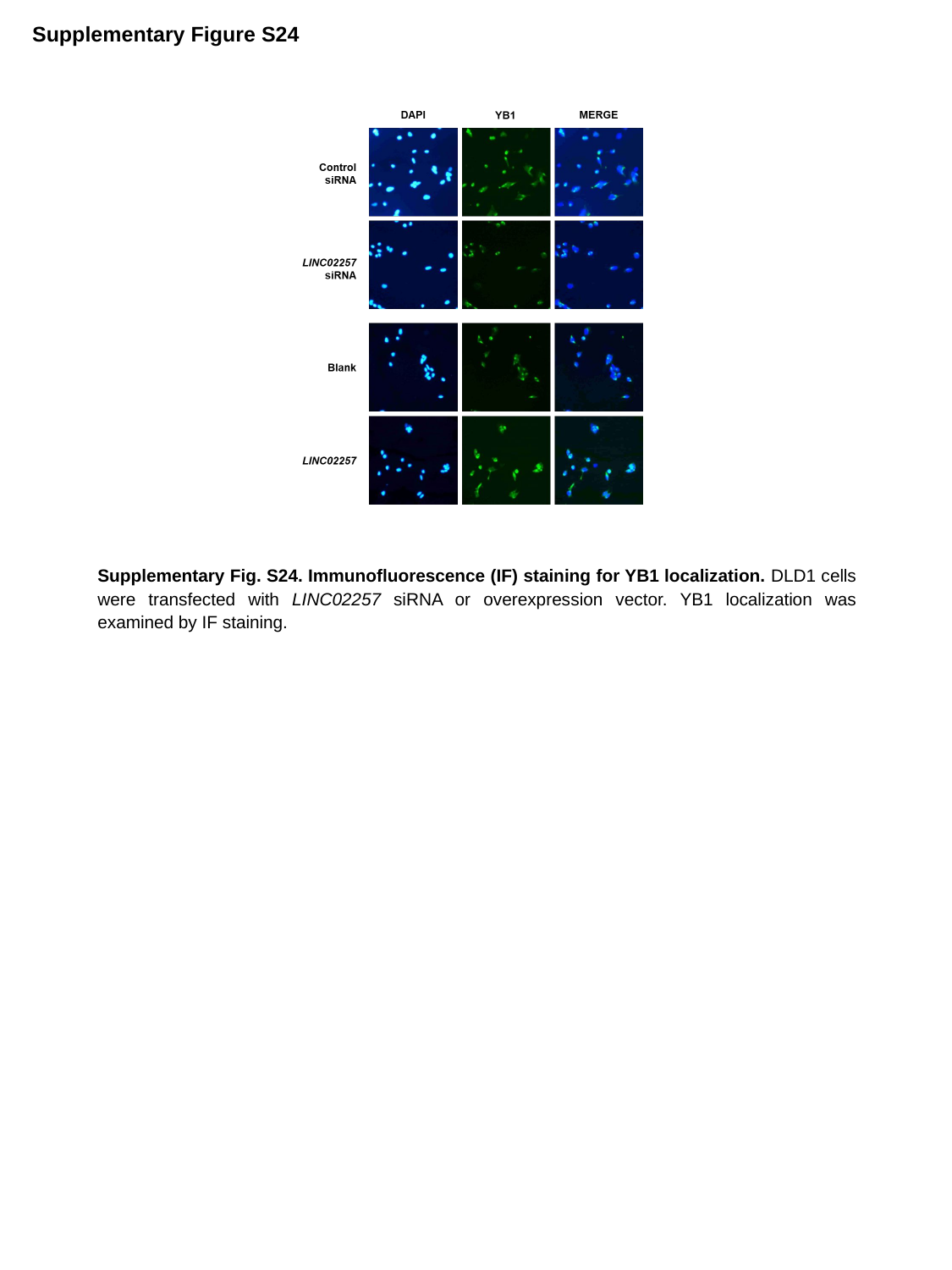

Supplementary Figure S24
Supplementary Fig. S24. Immunofluorescence (IF) staining for YB1 localization. DLD1 cells were transfected with LINC02257 siRNA or overexpression vector. YB1 localization was examined by IF staining.

## Slide 25
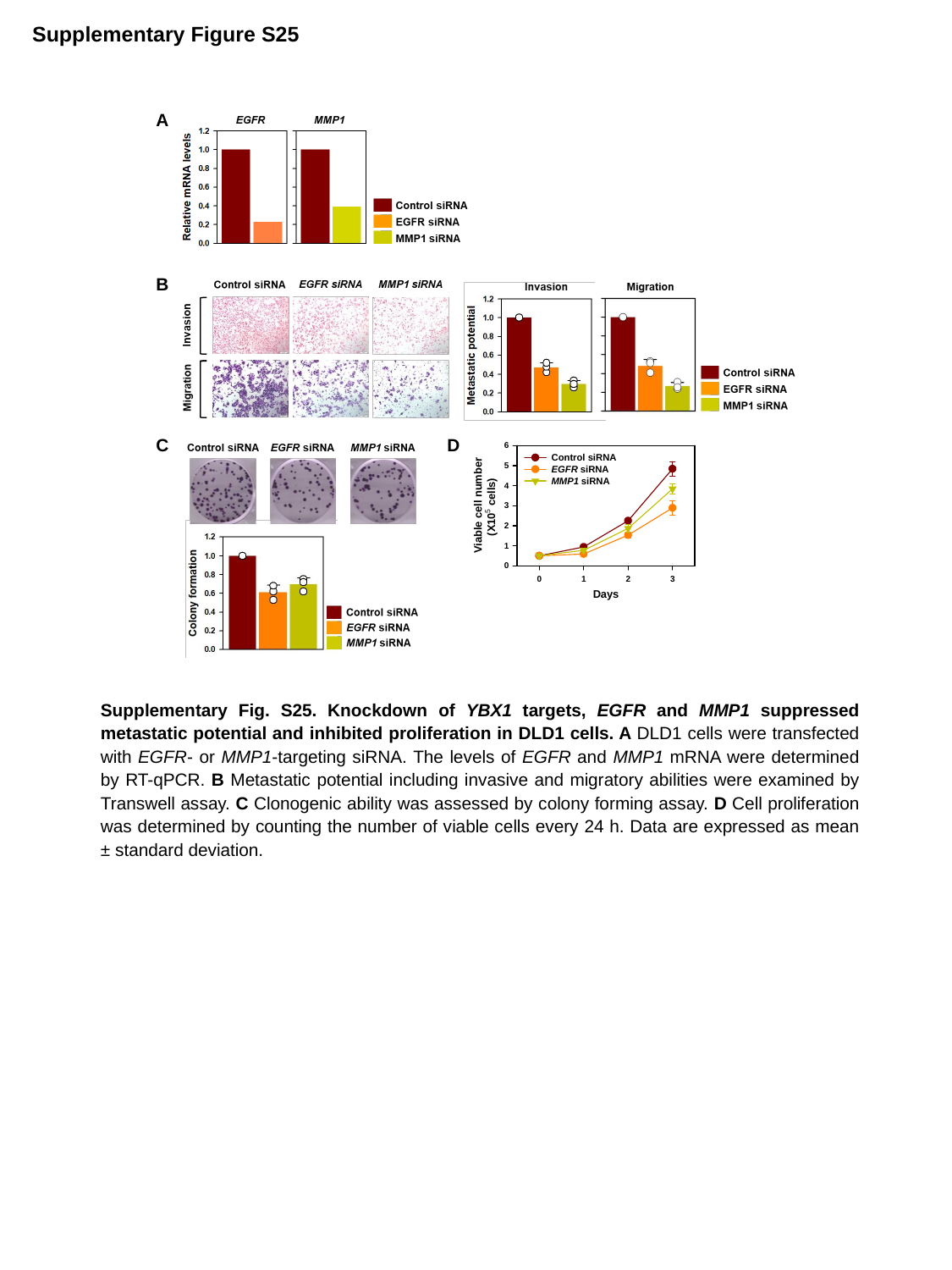

Supplementary Figure S25
A
B
C
D
Supplementary Fig. S25. Knockdown of YBX1 targets, EGFR and MMP1 suppressed metastatic potential and inhibited proliferation in DLD1 cells. A DLD1 cells were transfected with EGFR- or MMP1-targeting siRNA. The levels of EGFR and MMP1 mRNA were determined by RT-qPCR. B Metastatic potential including invasive and migratory abilities were examined by Transwell assay. C Clonogenic ability was assessed by colony forming assay. D Cell proliferation was determined by counting the number of viable cells every 24 h. Data are expressed as mean ± standard deviation.

## Slide 26
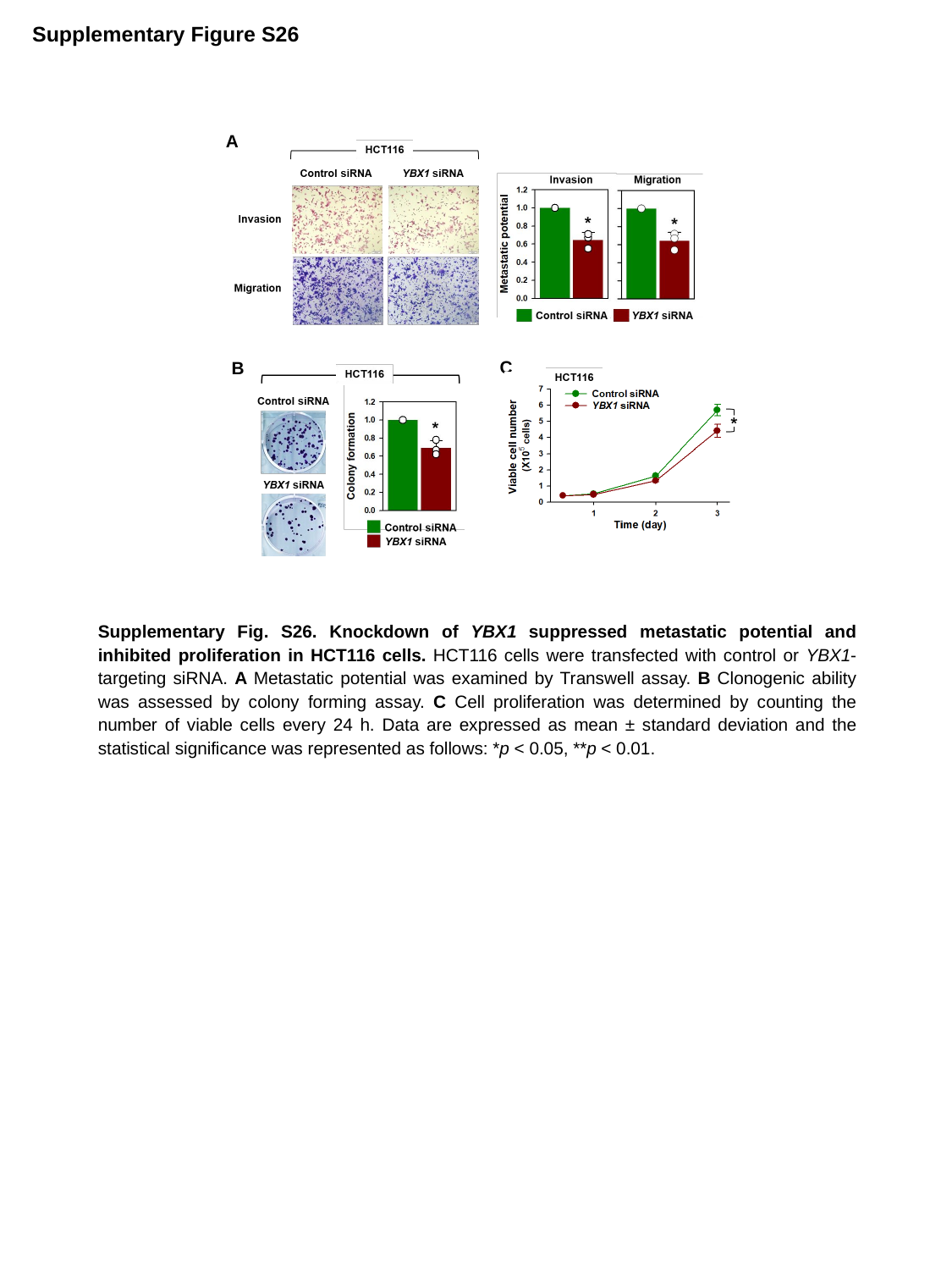

Supplementary Figure S26
A
C
B
Supplementary Fig. S26. Knockdown of YBX1 suppressed metastatic potential and inhibited proliferation in HCT116 cells. HCT116 cells were transfected with control or YBX1-targeting siRNA. A Metastatic potential was examined by Transwell assay. B Clonogenic ability was assessed by colony forming assay. C Cell proliferation was determined by counting the number of viable cells every 24 h. Data are expressed as mean ± standard deviation and the statistical significance was represented as follows: *p < 0.05, **p < 0.01.
