# Supplementary material for: LINC02257 regulates malignant phenotypes of colorectal cancer via interacting with miR-1273g-3p and YB1
Source: Cell Death Dis. 2024 Dec 18;15(12):895. doi: 10.1038/s41419-024-07259-4 (PMC11655847; doi:10.1038/s41419-024-07259-4)
Supplement: Supplementary file 4 — Uncropped WB images [file 41419_2024_7259_MOESM4_ESM.pptx]

## Slide 1
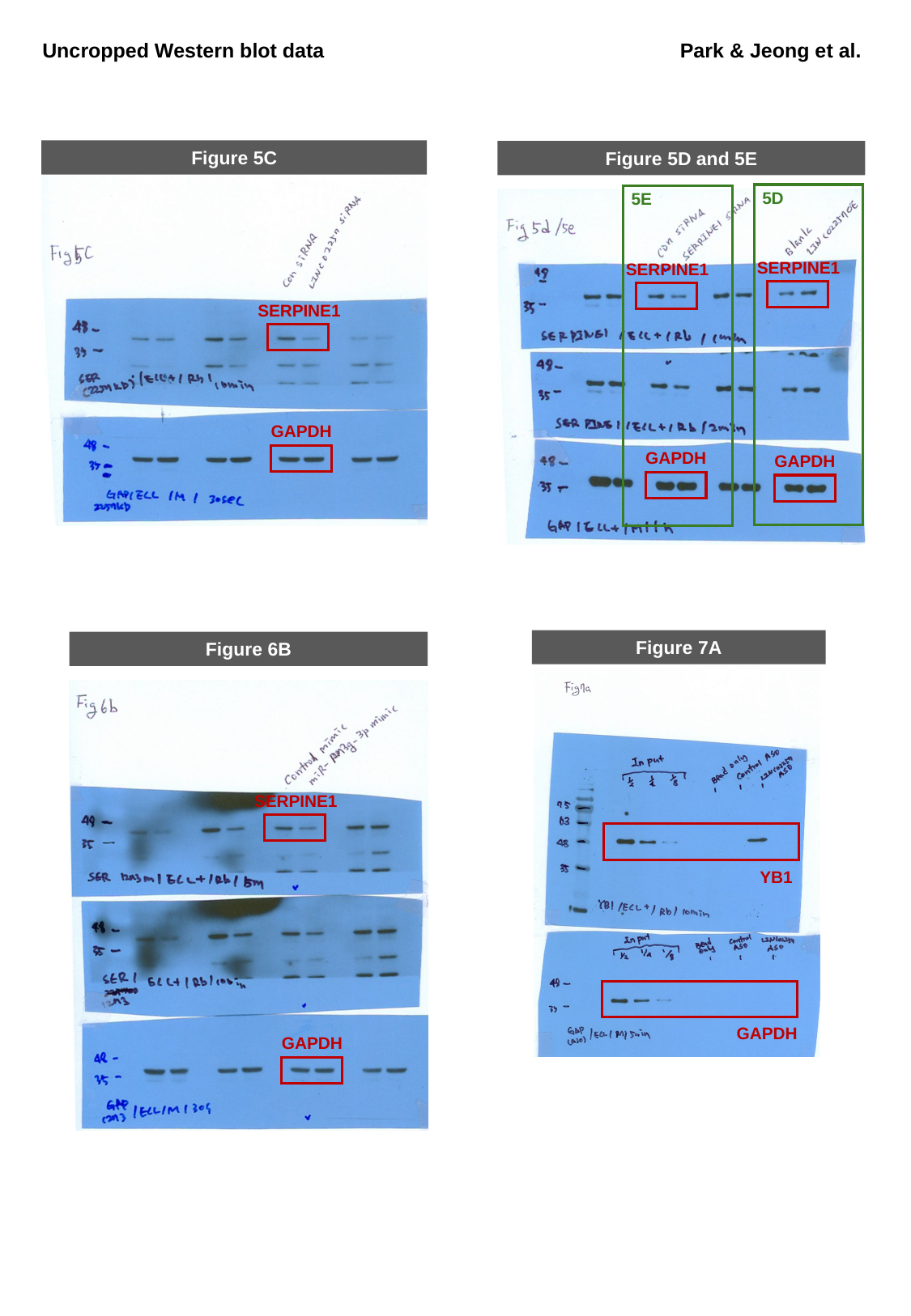

Uncropped Western blot data Park & Jeong et al.
Figure 5C
SERPINE1
GAPDH
Figure 5D and 5E
5D
5E
SERPINE1
SERPINE1
GAPDH
GAPDH
Figure 7A
YB1
GAPDH
Figure 6B
SERPINE1
GAPDH

## Slide 2
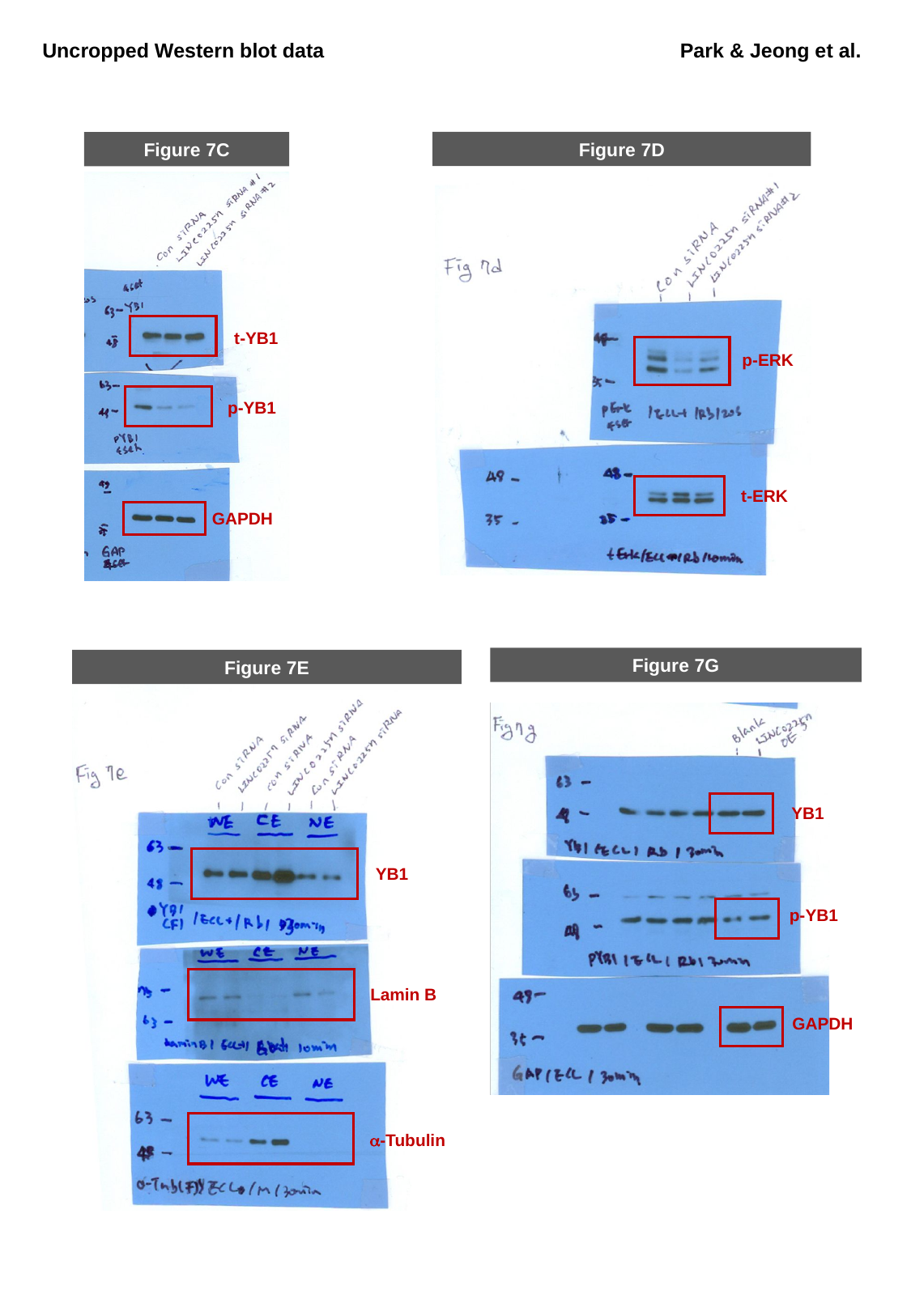

Uncropped Western blot data Park & Jeong et al.
Figure 7D
p-ERK
t-ERK
Figure 7C
t-YB1
p-YB1
GAPDH
Figure 7G
YB1
p-YB1
GAPDH
Figure 7E
YB1
Lamin B
a-Tubulin

## Slide 3
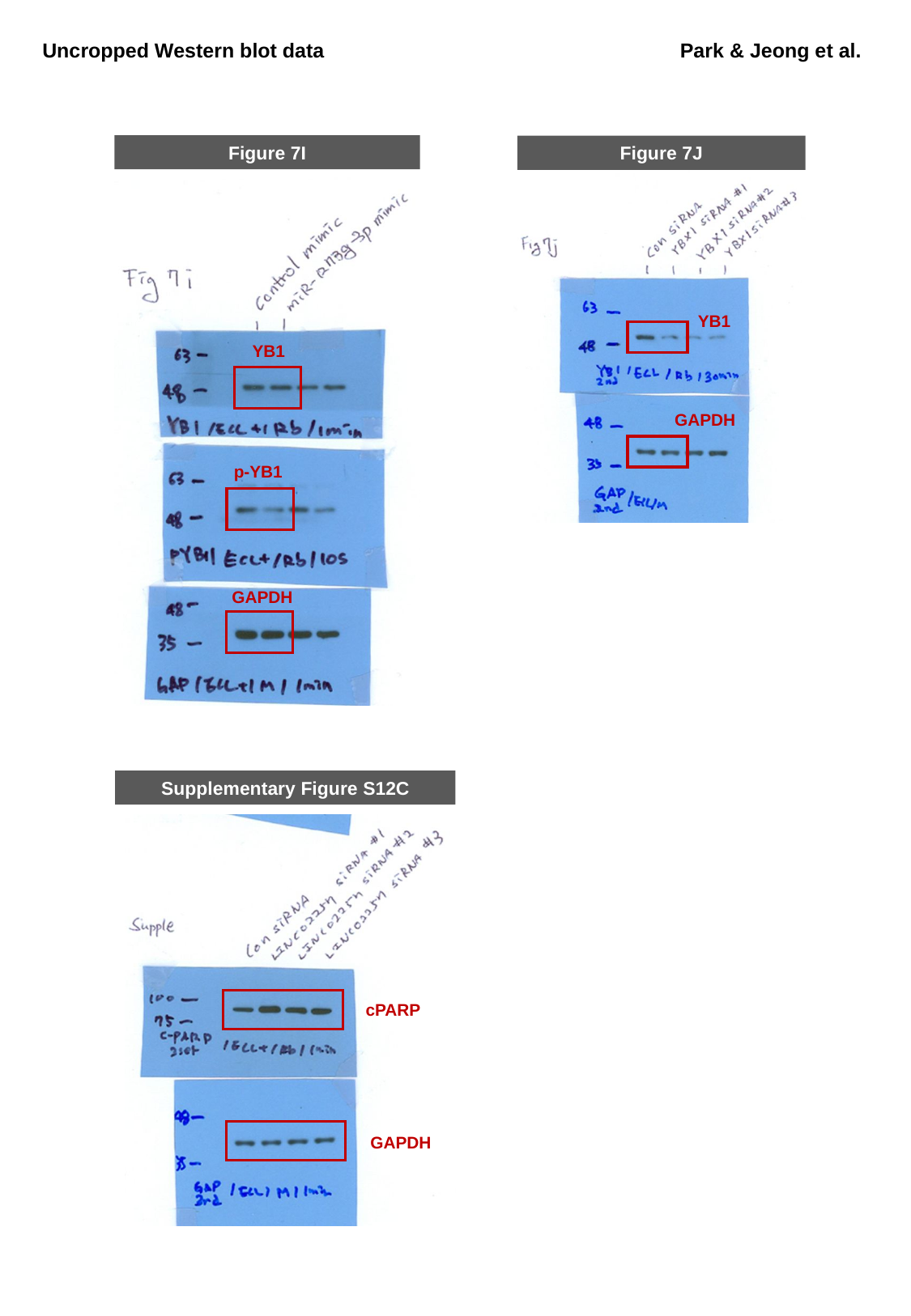

Uncropped Western blot data Park & Jeong et al.
Figure 7I
YB1
p-YB1
GAPDH
Figure 7J
YB1
GAPDH
Supplementary Figure S12C
cPARP
GAPDH
